# Nucleic acid drug vectors for diagnosis and treatment of brain diseases

**DOI:** 10.1038/s41392-022-01298-z

**Published:** 2023-01-17

**Authors:** Zhi-Guo Lu, Jie Shen, Jun Yang, Jing-Wen Wang, Rui-Chen Zhao, Tian-Lu Zhang, Jing Guo, Xin Zhang

**Affiliations:** 1 State Key Laboratory of Biochemical Engineering, Institute of Process Engineering, Chinese Academy of Sciences, Beijing, 100190 P.R. China; 2 University of Chinese Academy of Sciences, Beijing, 100049 P.R. China

**Keywords:** Gene delivery, Imaging, Nanobiotechnology

## Abstract

Nucleic acid drugs have the advantages of rich target selection, simple in design, good and enduring effect. They have been demonstrated to have irreplaceable superiority in brain disease treatment, while vectors are a decisive factor in therapeutic efficacy. Strict physiological barriers, such as degradation and clearance in circulation, blood-brain barrier, cellular uptake, endosome/lysosome barriers, release, obstruct the delivery of nucleic acid drugs to the brain by the vectors. Nucleic acid drugs against a single target are inefficient in treating brain diseases of complex pathogenesis. Differences between individual patients lead to severe uncertainties in brain disease treatment with nucleic acid drugs. In this Review, we briefly summarize the classification of nucleic acid drugs. Next, we discuss physiological barriers during drug delivery and universal coping strategies and introduce the application methods of these universal strategies to nucleic acid drug vectors. Subsequently, we explore nucleic acid drug-based multidrug regimens for the combination treatment of brain diseases and the construction of the corresponding vectors. In the following, we address the feasibility of patient stratification and personalized therapy through diagnostic information from medical imaging and the manner of introducing contrast agents into vectors. Finally, we take a perspective on the future feasibility and remaining challenges of vector-based integrated diagnosis and gene therapy for brain diseases.

## Introduction

The brain is the most complex organ in the human body in terms of structure and function and is the body’s central system that provides coordinated control of behaviors. The brain performs multiple functions such as sensory, motor control, arousal, homeostasis, motivation, learning, and memory. Therefore, brain diseases are diverse and have serious implications, such as brain tumors, neurodegenerative diseases (NDs), cerebrovascular diseases, brain injuries, psychiatric disorders, and infectious brain diseases. The complex brain structure and the body’s natural brain protection present two challenges in treating brain diseases: drug research and development against precise therapeutic targets and drug delivery to diseased regions.

Nucleic acid drugs with high specificity against the therapeutic target at the gene level are ideal medicines for treating brain diseases.^1–4^ However, the high density of negative charges and the instability caused by nucleases in vivo lead to the necessity of vector-dependent delivery of nucleic acid drugs in vivo.^5,6^ Conventional viral vectors have been ruled out due to safety risks in the brain.^7,8^ Therefore, the development of engineering non-viral vectors is the key to the application of nucleic acid drugs for gene therapy of brain diseases. The complex structure and diverse and vital functions of the brain require precise vector delivery. In addition, the strict physiological barriers during periphery-to-brain delivery also bring challenges and directions for vector development. Therefore, vector development relies on studying these physiological barriers, such as the immunosurveillance and protein adsorption in circulation, the blood-brain barrier (BBB), the cellular uptake barrier, the endosomal/lysosomal barrier, and the controlled drug release.^9–11^ Engineering vectors, such as polymeric nanoparticles (PNPs), lipid-based nanoparticles, inorganic nanoparticles, and extracellular vesicles (EVs), have been widely developed to deliver nucleic acid drugs to the brain. Regulating the vectors’ physicochemical properties and introducing functional groups, such as resistance to protein adsorption, evasion of immune surveillance, cell targeting, and microenvironmental sensitivity groups, into the vectors enhance their delivery performance to overcome physiological barriers.

It is unrealistic to develop only one type of vector for the efficient treatment of brain diseases in different patients due to individual patient differences.^11,12^ It is also unrealistic to efficiently treat different patients’ brain diseases with nucleic acid drugs targeting a single therapeutic target. Individual patient differences are mainly reflected in variable sensitivity to the same therapeutic target and diverse delivery properties for the same vector. In addition, single drugs are frequently ineffective in treating diseases with complex pathogenesis due to the limitations of drug types and targets. Therefore, the best strategy to overcome patient heterogeneity is utilizing patient information for accurate stratification, screening appropriate vectors and therapeutic targets, and providing precise and personalized treatment. Access to comprehensive and accurate diagnostic information in time is the key to precise and personalized treatment. It is feasible to endow medical imaging signals with these vectors (nanotheranostics).^12,13^ Medical imaging can diagnose the accumulation of vectors in brain lesions and the pharmacokinetics and pharmacodynamics of drugs in a non-invasive and real-time manner so that vectors and drugs can be screened for their suitability for the patient. The development of nanotheranostics is focused on gaining more comprehensive and accurate diagnostic information through medical imaging.

**Fig. 1:**
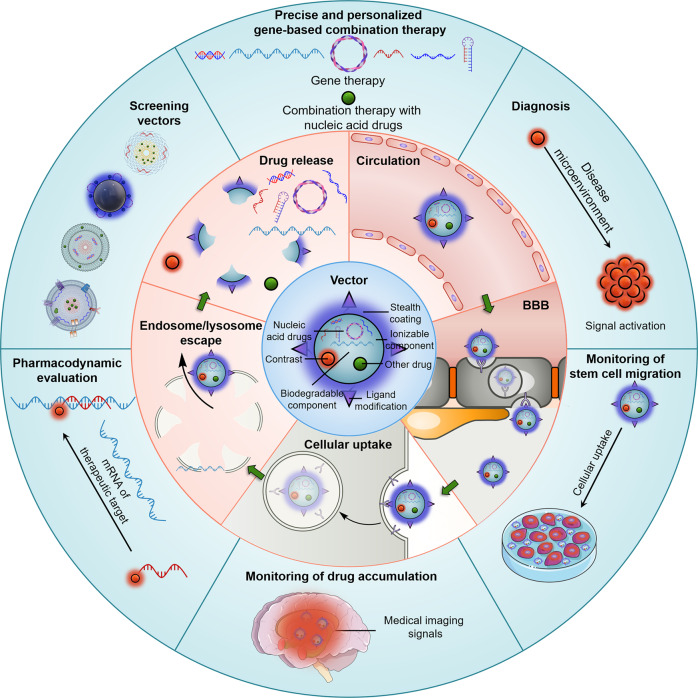
Mechanisms of nucleic acid drug-based combination therapy and medical imaging contrast-based diagnosis for precise and personalized treatment of brain diseases. The inner ring shows a conventional contrast-labeled nucleic acid drug vector. The middle ring demonstrates the biological barriers that nucleic acid drug vectors face for delivery to the brain. The outer ring illustrates how medical imaging-based diagnostics contribute to precise and personalized therapy

This Review focuses on advances in engineering vectors for brain disease diagnosis and gene therapy, with a highlight on overcoming physiological barriers to delivery through precise vector design and overcoming patient heterogeneity through accurate diagnostic information and rational therapeutic targeting (Fig. 1). In addition, this Review discusses physiological barriers to the delivery of nucleic acid drugs to the brain and generalized strategies for overcoming these barriers. In presenting nucleic acid drug vectors applied to treating brain diseases, we explore how these generalized strategies are achieved by designing and functionalizing vectors. The Review further explores the regimen of nucleic acid drug-contained multidrug combinations for brain disease treatment, especially the vector design for achieving optimal multidrug efficacy. Finally, this Review describes the research advances, potentials, and current limitations of integrating brain disease diagnosis and gene therapy through nanotheranostics for patient stratification and personalized precision therapy and discusses perspectives for the future development of nanotheranostics.

## Classification of nucleic acid drugs

The sequence-specific ability of nucleic acid drugs to treat diseases at the genetic level enables more precise and individualized disease treatment. The advantages of nucleic acid drugs over small-molecule drugs and protein-based drugs are irreplaceable. First, the nucleic acid drug sequences are designed based on the sequences of the target genes. Thus, through Watson-Crick base pairing, they can bind to the target genes with high specificity and act on the disease treatment targets at the gene level. By contrast, small-molecule drugs and protein-based drugs must recognize the complex spatial conformation of the target protein. Therefore, theoretically, nucleic acid drugs can act on any therapeutic target. Many therapeutic targets are inaccessible to small-molecule drugs and protein drugs due to their inability to access specific protein conformations. Besides, small molecule and protein drugs primarily target cell surface receptors and circulating proteins and therefore have limited therapeutic effects. In addition, due to the availability of target gene sequences, nucleic acid drugs based on the gene sequence have shorter research and development times and higher success rates. Moreover, nucleic acid drugs act at the therapeutic target’s genetic level. Nucleic acid drugs have a long half-life since they can theoretically act on the target gene multiple times.

Small interfering RNAs (siRNAs), microRNAs (miRNAs), and antisense oligonucleotides (ASOs) are typical nucleic acid drugs that inhibit target genes by pairing with the target RNA. Plasmid DNAs (pDNAs) and messenger RNAs (mRNAs), on the other hand, are commonly used to increase the expression of target genes. Clustered regularly interspaced short palindromic repeats (CRISPR)/Cas systems are more versatile and can increase, repress, and correct the expression of target genes. This section focuses on the therapeutic mechanisms and design of nucleic acid drugs such as siRNA, miRNA, ASO, mRNA, pDNA, and the CRISPR/Cas system and compares these nucleic acid drugs.

### siRNA

Through the RNA interference (RNAi) mechanism, siRNA inhibits the expression of target mRNAs in the cytoplasm of target cells, resulting in gene silencing effects. RNAi is a cellular process that represses gene expression in vivo by promoting mRNA degradation or inhibiting mRNA expression.^14^ In the cytoplasm, Dicer (a specialized ribonuclease (RNase) III-like enzyme) converts dsRNA to siRNAs (21~23 nt double-stranded RNA with 2 nucleotides 3’ overhang). siRNA interacts with the RNA-induced silencing complex (RISC). RISC’s nucleic acid endonuclease Argonaute 2 (AGO2) cleaves the sense strand of the siRNA. Through Watson-Crick base pairing, the intact antisense strand acts as a guide strand to recognize the target mRNA. RISC AGO2 continues to cleave mRNA, thereby silencing the target gene.^15,16^ Furthermore, after being delivered to the cytoplasm, short hairpin RNAs (shRNAs), a type of stem-loop RNA expressed in the nucleus, silence target genes via the same RNAi mechanism as synthetic siRNAs.^17^ In addition, Dicer can mediate the production of microRNAs in the cytoplasm. The miRNA gene is transcribed by RNA polymerase II into primary miRNA (pri-miRNA), which is then cleaved by Drosha into precursor miRNA (pre-miRNA). After Exportin 5 transports pre-miRNA to cells, Dicer converts it to miRNA.^18^ Consequently, miRNA is also a nucleic acid drug based on the RNA interference mechanism, as will be discussed below. Synthetic siRNA drugs, on the other hand, circumvent Dicer processing and bind directly to RISC, silencing target genes through the endogenous RNAi machinery.

Optimizing the sequence design of siRNAs is essential for enhancing the gene-silencing effect and minimizing off-target effects. siRNAs typically consist of 21 to 23 nucleotides. Although increasing the length of dsRNA can promote its gene-silencing effect,^19^ long dsRNAs (more than 30 nucleotides) activate protein kinase R (PKR) and further activate the interferon pathway, which leads to off-target mRNA degradation and apoptosis.^20^ Therefore, long dsRNAs are not considered in therapeutic applications. siRNA requires the antisense strand to function as the guide strand in order to recognize the mRNA target, while the sense strand is degraded. However, both the sense and antisense strands have the potential to serve as the mRNA’s guide strand. Therefore, the antisense strand will be degraded, and the sense strand will serve as the guide strand to recognize the mRNA if the loading direction is incorrect, which will have an off-target effect and prevent the siRNA from mediating the degradation of the target mRNA. Therefore, the guide strand of siRNA must correctly bind to RISC. Using the relatively unstable strand at the 5’ ends as the antisense strand and the more stable strand at the 5’ ends as the sense strand, the unstable antisense strand can selectively bind to RISC.^21,22^ In addition, the AGO2 of RISC prefers to bind strands with U or A at position one at the 5’ ends. Therefore, the 5’ ends of the guide strand should also have U or A.^23^

Unmodified siRNA is highly susceptible to degradation by nucleases in vivo, which is one of the most significant limitations of the clinical applications of siRNA. siRNA modification contributes to siRNA resistance to nuclease degradation. The position of siRNA modification is the first consideration. The 5’ phosphate, the 5’ proximal region, and the central positions of the guide strand are crucial for RNA interaction with RISC and AGO2; therefore, avoid chemically modifying these locations.^24,25^ In contrast, chemical modification of the 3’ proximal region and the 3’ overhang of the guide strand has no impact on the activity of siRNA. Moreover, chemical modifications of the entire sense strand are permitted.^26^ Ribose 2’-OH group modification, locked and unlocked nucleic acids, and phosphorothioate (PS) modification are common chemical modifications of siRNA. Ribose 2’-OH group modification consists of substituting the ribose 2’-OH group with other groups. Alternating 2’-O-methyl and 2’-fluoro substitutions can significantly improve the resistance of siRNA to enzymatic degradation, reduce the immune response, and have no effect on siRNA’s ability to silence genes.^27^ PS modification of the siRNA backbone increases the siRNA’s stability. However, PS modifications also exacerbate siRNA toxicity and reduce the RNAi effect of siRNAs. In addition, PS modifications are commonly used as backbone modifications in ASOs and will be described in detail in the ASO discussion. The Review by Lieberman et al. provides a more comprehensive and in-depth discussion of siRNA.^15^

### miRNA

miRNAs silence target genes via an RNAi mechanism similar to that of siRNAs but not identical. Following a series of cellular processes, miRNA genes are converted into miRNA (18~25 nt double-stranded RNA with 2 nucleotides 3’ overhang). After interacting with RISC, miRNA forms the miRISC complex. After miRNA double-strand opening, unlike siRNA, the sense strand is not degraded by AGO2 but is instead released. The remaining miRISC is directed to bind to mRNA by the miRNA antisense strand. In contrast to the antisense strand of siRNA, which must be entirely complementary to mRNA, the antisense strand of miRNA needs only be partially complementary to mRNA.^28^ A single miRNA sequence can therefore recognize multiple mRNA sequences and exert silent effects on multiple genes.^29^ In addition, after recognizing mRNAs, miRISC typically silences target genes via translational repression and degradation, and so on.^28,30^ In rare instances, miRISC is highly complementary to mRNA and mediates the cleavage of mRNA by AGO2 in a manner analogous to siRNA.^28^

A single miRNA can recognize and regulate the translation of multiple mRNAs. Over half of human genes encoding proteins contain at least one conserved miRNA binding site. In addition, these genes contain numerous miRNA-binding sites that are not conserved. Therefore, miRNAs can regulate the expression of the vast majority of proteins.^28^ miRNA dysfunction can lead to a variety of diseases, including cancer, cardiovascular disease, and neurodegenerative disease.^31–33^ Therefore, miRNAs can be used as biomarkers for the prediction, diagnosis, and prognosis of many diseases, in addition to being therapeutic agents. Through the function of endogenous miRNAs, synthetic miRNAs are currently used as therapeutic agents to treat diseases. Therefore, the sequences of miRNAs and endogenous miRNAs are typically identical. Although the antisense strand of miRNA plays a decisive role in RNAi, a double-stranded miRNA containing both the sense and antisense strands is significantly more potent than a therapeutic agent containing only the antisense strand of miRNA.^34,35^ Therefore, it is necessary to design double-stranded miRNAs. Since pri-miRNA is degraded in the nucleus to pre-miRNA. The subsequent cellular processes are conducted in the cytoplasm. Therefore, pre-miRNA and miRNA do not need to be transported to the nucleus in contrast to pri-miRNA. Moreover, compared to pre-miRNA, miRNA does not undergo Dicer processing. In addition, pre-miRNA contains 70~100 nucleotides, which is 3~5 times more than miRNA, adding to the difficulty of RNA synthesis and delivery. Therefore, double-stranded miRNA with the same sequence as endogenous miRNA is a more rational sequence design than pri-miRNA and pre-miRNA.

As with siRNA, the susceptibility of miRNA delivery in vivo to nuclease degradation is a bottleneck limiting the efficacy of miRNA. In addition, miRNAs may activate the innate immune system, resulting in severe side effects. Since the sequence of synthetic miRNAs is referenced to endogenous miRNA sequences, it is challenging to reduce side effects by optimizing miRNA sequences. Chemical modification is a prevalent strategy for overcoming in vivo side effects and nuclease-mediated degradation of microRNAs. Since the chemical modification of miRNA is essentially identical to that of siRNA, it will not be described in this section. Lam et al. comprehensively compared siRNA and miRNA in their Review.^18^

### ASO

ASOs are an oligonucleotide drug class with a length range of 8~50 bp that resemble single-stranded DNA. Zamecnik and Stephenson proposed using complementary sequences in ASO to knock out genes in 1978. They prepared an oligonucleotide complementary to the Rous sarcoma virus’s 35 S RNA and discovered that this oligonucleotide could prevent the virus from replicating.^36,37^ Theoretically, ASO can act with a high specificity on its receptor sequence, particularly on receptors resistant to conventional drugs.^38^ As research on RNA biosynthesis, processing, function, and degradation advances, the variety of receptor RNAs used in ASO therapies expands. In addition to mRNAs and ribosomal RNAs (rRNAs) that translate proteins, these receptor RNAs also include non-coding RNAs such as miRNAs, Piwi-interacting RNAs (piRNAs), and small nuclear RNAs (snRNAs).

The phosphodiester backbone of unmodified ASOs is extraordinarily susceptible to nuclease degradation in vivo. For ASOs to be utilized in disease therapy, their backbones must be modified to improve their stability. The most prevalent backbone modifications are PS linkages and modifications in phosphorodiamidate morpholino oligomer (PMO). The substitution of phosphate linkages for PS linkages can increase the nuclease resistance of ASOs. PS linkages can also improve the pharmacokinetic properties of ASOs by regulating their interaction with plasma proteins.^39,40^ ASOs can be made more stable by substituting morpholino groups for sugars and phosphorodiamidate linkages for phosphate linkages in PMO.^41^ In addition, conjugated ligand molecules on ASOs can increase the ASO uptake in diseased cells.^42,43^ The most representative ligand modification is N-acetylgalactosamine (GalNAc) conjugation at the 3’ or 5’ terminus of ASOs. By interacting with the asialoglycoprotein receptor, the GalNAc moiety promotes the accumulation of ASOs in the liver.^44,45^

The mechanisms of action of ASOs fall into two distinct categories. Various approved PS ASOs function through occupancy-mediated degradation of the target RNA, including RNase H1-mediated cleavage. RNase H1 enzymes are capable of degrading RNA-DNA-like duplexes specifically. ASOs form RNA-DNA complexes by complementary pairing with target mRNAs, thereby recruiting RNase H1 to cleave the target mRNAs.^46^ RNase H1 enzymes do not break non-target RNAs or ASOs that are not RNA.^47^ Consequently, ASOs can continue to degrade mRNA targets. Additionally, ASO can form double-stranded RNA-DNA complexes with other RNAs, such as miRNAs, through Watson-Crick pairing, thereby mediating RNAse H1-directed cleavage of the target miRNAs.^48,49^ ASOs also function through occupancy-only mechanisms. Some ASOs do not induce target mRNA degradation after pairing with the mRNAs but instead bind to the 5’ cap or polyadenylated tail of mRNAs, thereby affecting mRNA translation and stability.^50^ ASOs can also form complexes with pre-mRNA to influence the recruitment of splicing factors, thereby altering the regular splicing pattern of mRNA.^51^ The FDA-approved drug Nusinersen (Spinraza) is a typical example of a treatment for spinal muscular atrophy based on occupancy-only mechanisms.^52,53^

The comparison between ASO and siRNA is significant. Through a synthetic antisense strand, both ASO and siRNA identify and mediate the degradation of the target RNA. ASO is a single-stranded structure containing hydrophobic nucleobases and hydrophilic PS inter-nucleotide linkages. Consequently, ASO exists in multiple conformations in vivo. siRNA, on the other hand, is a rigid double-stranded structure with about twice the molecular weight of ASO. The sense strand of siRNA prevents the rapid degradation of its antisense strand, which functions as a guide. After ASO forms RNA-DNA-like duplexes with target RNAs via complementary base pairing, RNase H1 is recruited to cleave the RNA strands on the RNA-DNA-like duplexes. siRNA, on the other hand, cleaves the sense strand of siRNA and the target mRNA successively via AGO2 on RISC. Therefore, siRNA-mediated silencing of target genes is more complex than ASO. In addition, RNase H1 associated with ASO can cleave the target RNA rapidly. In contrast, AGO2 associated with siRNAs cleaves the target mRNA relatively slowly. However, AGO2 can bind to the antisense strand of siRNA for a period, thereby preparing other target mRNAs for degradation. In addition to mediating the cleavage of mRNAs, ASO has been designed to mediate the degradation of other non-coding RNAs involved in disease therapy, such as miRNAs. In contrast, siRNAs are still mainly used to treat diseases by mediating the degradation of mRNAs. Crooke et al. provided a comprehensive and in-depth discussion of advances in antisense technology and compared ASO and siRNA in their Review.^54^

### mRNA

mRNA is a long, single-stranded polynucleotide between 500 and 5000 nucleotides in length. It is composed of a 5’ cap, 5’ untranslated regions (UTR), an open reading frame (ORF), a 3’ UTR, and a 3’ poly(A) tail. After entering cells, the ORF region of the mRNA can express the encoded protein, while the other regions protect the mRNA and regulate its translation.^55^ The mRNA was discovered for the first time in 1961, but it was not successfully transfected in vivo after intramuscular injection in mice until 1990.^56^ Since then, mRNA has been investigated for use in viral vaccines, cancer immunotherapies, and protein replacement therapies, among other applications.^57^ mRNA can be produced through in vitro transcription (IVT). Unlike plasmid DNA, mRNA can be translated into the cytoplasm without entering the cell nucleus. Moreover, it does not integrate into the genome, reducing the risk of carcinogenesis.^58^

In order to achieve therapeutic effects, the mRNA must enter the target cells and express adequate levels of proteins. Due to its highly anionic nature and extreme sensitivity to enzymes, mRNA’s poor delivery and inefficient uptake in vivo prevent it from being used as an effective therapeutic agent. Various biomaterials, including lipids, lipid-like materials, polymers, and protein derivatives, have been developed for mRNA delivery in the present study.^2,59,60^ For instance, two coronavirus disease 2019 (COVID-19) vaccines (mRNA-1273 and BNT162b) that were approved in 2020 utilized lipid nanoparticles for mRNA delivery.^61,62^ Through the assembly process, the mRNA was encapsulated with ionizable lipid, PEGylated lipid, helper lipid, and cholesterol. This LNP system safeguarded the mRNA and transported it to the target cells for translation. Inspired by the mRNA vaccines for COVID-19, numerous mRNA delivery systems for viral prevention and therapy, cancer immunotherapy, and the treatment of other diseases have been developed. The Review by Huang et al. provides a more comprehensive and in-depth discussion of the challenge and prospect of mRNA therapeutics.^63^

### pDNA

pDNA is circular DNA, typically between 2000 and 20000 base pairs in length, with a relatively stable structure. As an efficient gene vector, pDNA is now extensively studied as a tool for DNA vaccines and pDNA gene therapy. DNA vaccines activate protective immunity by cloning a specific gene that encodes the target protein into pDNA, which then induces a cell-mediated immune response, activating protective immunity.^64^ pDNA-based gene therapy is the cloning of therapeutically-functional target genes into pDNA to treat genetically-based diseases.^65^

pDNA is typically extracted from recombinant Escherichia coli (E. coli) and used after RNA, proteins, and endotoxins are removed. Elements required for maintenance and propagation within bacteria and elements required for expression within mammals are the primary constituent sites of pDNA. Components of bacteria contain replication sources, antibiotic resistance genes, and other plasmid amplification markers. Mammalian expression elements consist of mammalian or viral enhancer/promoter sequences for gene expression; 5’UTR, including introns, reporter transgenes, target genes, and polyadenylation sequences.^66^ Typically, a specific universal plasmid vector serves as a foundation to which experimentally relevant components are added or modified. A DNA fragment encoding a target protein, for instance, can be cloned into a plasmid vector. Under specific conditions, pDNA is incorporated into the host chromosome and passed on to the offspring via chromosome replication and cell division, while the gene it encodes is translated into a protein. Additionally, miRNA gene fragments can be cloned into plasmid vectors in order to express miRNA via promoters and regulate the expression of related genes. In general, the smaller the pDNA size, the more effective the transfection. pDNA utilizes the cell’s transcriptional machinery to produce proteins and functions for a longer duration than protein complementation therapy. pDNA is more cost-effective, transportable, and stable than mRNA, but the larger size of pDNA increases the risk of introducing other components.^67^

### CRISPR/Cas system

CRISPR, a repetitive sequence within the genome of prokaryotes, was discovered in 1987 in E. coli. Jennifer and Emmanuelle discovered the mechanism of the CRISPR (Type II) system in 2012 and confirmed that only Cas9 nuclease and sgRNA are necessary for the system to function. Subsequently, Feng Zhang et al. rapidly applied CRISPR-Cas9 technology to mammalian cells, significantly exploring the application potential of CRISPR, the most potent gene-editing tool with functions including gene knockout, gene knock-in, gene repression or activation, and multiple gene editing.

CRISPR type II, the CRISPR-Cas9 system, is currently the most prevalently used system. The sgRNA is responsible for binding to the target DNA near the protospacer adjacent motif (PAM) and subsequently activating the HNH and RuvC structural domains of the Cas9 protease to shear the target DNA double-stranded. DNA is repaired by non-homologous end joining (NHEJ) or homology-directed repair (HDR) following shearing.^68^ NHEJ produces small insertions or deletions (indels) at the cleavage site, whereas HDR replaces the target allele with an alternative sequence.^69^ Cas9 protease and sgRNA can function in three different forms: 1) co-encoding into the same pDNA; 2) Cas9 mRNA and sgRNA; 3) Ribonucleoprotein complex (RNP) containing Cas9-sgRNA. pDNA is relatively stable and inexpensive, but the operation is slow and introduces additional components. Cas9 mRNA and sgRNA have the advantage of not requiring entry into the RNPs, allowing them to function rapidly and reduce off-target efficiency significantly. CRISPR-Cas9 can target genes to produce permanent knockdown,^70^ in contrast to the reversible gene downregulation produced by RNAi technology. Additionally, CRISPR can be utilized as a gene interference tool, known as CRISPR interference (CRISPRi). CRISPRi relies predominantly on the inactive dead Cas9 (dCas9) nuclease. Guided by sgRNA, the dCas9 nuclease can bind to the HNH and RuvC structural domains, but it is incapable of gene cleavage and serves only as a reversible repressor of target gene expression.^71^ CRISPRi differs from RNAi in terms of its mechanism of action; RNAi inhibits the expression of already-transcribed mRNAs, whereas CRISPRi directly inhibits transcription.

Cas12, Cas13, and Cas14 nucleases, in addition to Cas12, Cas13, and Cas14 nucleases, have been discovered as gene-editing tools as research advances. In addition to acting against dsDNA, Cas12 also produces cleavage of ssDNA.^72–74^ Cas13 nuclease cleaves ssRNA, and Cas14 nuclease cleaves ssDNA. These nucleases (Cas12 1200~1300 amino acids; Cas13 700~1000 amino acids; Cas14 500 amino acids) are smaller than Cas9 nucleases (1000~1700 amino acids), so they are more easily delivered and significantly expand the range of CRISPR applications. Compared to zinc finger nucleases (ZFNs) technology and transcription activator-like effector (TALEN) technology, CRISPR has dramatically simplified gene editing and accelerated the development of biotechnology and genetic engineering. However, the delivery of CRISPR may continue to pose the most significant barrier to its widespread application. The progress of CRISPR technology is discussed in detail and comprehensively in their Review by Koonin et al..^75^

In general, nucleic acid drugs have a more comprehensive range of therapeutic targets, greater target specificity, and more efficient and long-lasting therapeutic effects than traditional small molecule and protein drugs. Therefore, nucleic acid drugs have considerable potential for treating brain disorders. However, the strong negative charge and susceptibility to nuclease degradation of nucleic acid drugs limit their uptake by focal cells. Therefore, it is essential to design rational carriers to deliver nucleic acid drugs in vivo efficiently. Due to their different molecular structures and molecular weights, different types of nucleic acids necessitate diverse loading strategies. Small nucleic acid drugs, such as siRNAs, miRNAs, and ASOs, can be chemically modified to increase their stability and conjugated with ligand molecules to facilitate their accumulation in the foci. In contrast, nucleic acid drugs with relatively large molecular weights, such as mRNAs and pDNAs, are difficult to chemically modify and lend themselves to loading and in vivo delivery by vectors with large cavity structures, such as liposomes.

## Biological barriers to systemic delivery of nucleic acid drugs for brain diseases

**Fig. 2:**
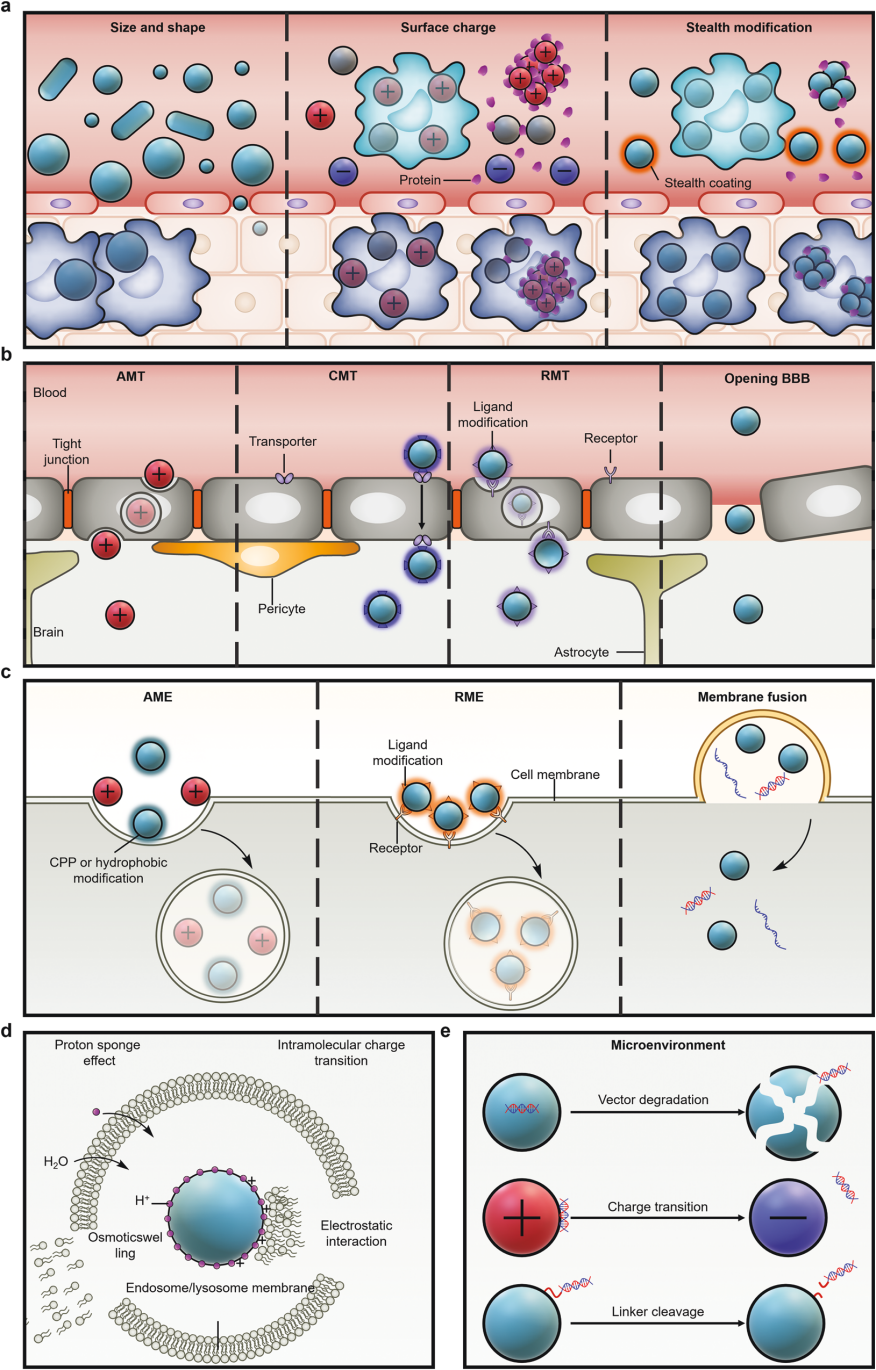
Schematic illustration of the biological barriers to systemic delivery of nucleic acid drugs for brain diseases and strategies to overcome these barriers. **a** Degradation and clearance in circulation. **b** BBB. **c** Cellular uptake. **d** Endosome/lysosome barriers. **e** Nucleic acid drug release

Nucleic acid drugs are susceptible to degradation by nucleases and are thus unstable. In addition, the high density of negative charges in nucleic acid drugs makes it difficult for them to be taken up by target cells. Unlike other drugs, the intrinsic nature of nucleic acid drugs necessitates delivery through vectors. The positive therapeutic effects of brain diseases rely heavily on the ability of the vectors to direct the nucleic acid drugs steadily to a specific site. This ability determines the therapeutic efficacy and side effects of the nucleic acid drugs and the appropriate doses of nucleic acid drugs needed to bring about effective responses. However, the delivery of nucleic acid drugs to brain diseases is a complex process. It requires vectors that can overcome multiple biological barriers. The barriers encountered by the vectors largely depend on the route of administration. Local administrations can bypass various biological barriers, including the BBB. However, they are often invasive and involve complex techniques, which limit their application to local administrations. Therefore, this section focuses on the biological barriers faced by systemic administrations (Fig. 2).

### Degradation and clearance in circulation

In the circulation, vascular physio-anatomy, blood flow, cells and biomolecules in the blood, and phagocytic cells influence the stability and clearance of vectors. Vectors are mainly cleared by the mononuclear phagocytic system (MPS)^76^ or reticuloendothelial system (RES).^77,78^ The interaction of vectors with MPS stimulates immune responses,^79^ which induces toxic effects such as inflammation or tissue damage.^80,81^ The physicochemical properties of the nanoparticles, such as size, shape, surface charge, surface modification, and bionic coating, determine the interaction of the vectors with the above microenvironment. Therefore, the regulation of the physicochemical properties of vectors is crucial for efficient gene therapy with low side effects for brain diseases.

The physicochemical properties of the vectors are important influencing factors for the distribution and clearance of drugs in various organs.^11^ Especially, the vectors’ size is closely related to the biodistribution of the drug, which is mainly determined by the vascular physio-anatomy.^82^ Although the capillary fenestraes of the glomerulus are 10~200 nm, the basal lamina only allows the clearance of vectors smaller than 5 nm to penetrate.^83,84^ In contrast, the capillary fenestrae of the hepatic sinusoids are 100~200 nm, which makes the liver the major clearance organ.^85^ 10~20 nm of vectors are rapidly filtered by the liver and cleared from the blood.^83^ Kupffer cells in the liver are responsible for removing nanoparticles above 200 nm.^82^ The capillaries of the spleen are discontinuous. Vectors larger than 200 nm are usually taken up by the spleen.^86^ In addition, vectors larger than 200 nm may be recognized and removed by the RES.^82^ In contrast, the rapidly building capillaries in the tumor region are mostly porous, thus facilitating the penetration of vectors in the tumor,^87^ which is one of the reasons for the high penetration of drugs in tumors. Therefore, the proper size helps prolong the delivery time of nucleic acid drugs by vectors in the blood circulation.

The shape also affects the circulation time of the vectors, which may be caused by blood flow-induced shear stress.^11^ However, the effect of the shape of the vectors on the blood circulation time is unclear. Discher et al. reported that filomicelles had a circulation time of one week in vivo. In contrast, spherical nanoparticles consisting of similar poly(ethylene glycol) (PEG)-based amphiphilic block copolymers were rapidly removed within two days.^88^ The researchers thought the strong hydrodynamic effects on filomicelles might contribute to the long circulation. Compared to spherical nanoparticles, hydrodynamic shears caused cylindrical filomicelles to flow, which makes filomicelles resistant to staying on the surface of macrophages and being taken up. Ghandehari et al. found that the circulation of gold nanorods was longer than that of gold nanospheres.^89^ The area under the curve (AUC) of gold nanorods reached 4.1 times the AUC of gold nanospheres. In addition, gold nanorods were mainly distributed in the liver and spleen and may be cleared through RES. In general, non-spherical vectors tend to possess a more extended circulation than spherical vectors.

Surface charge is an essential factor affecting vector clearance. Neutral and negatively surface-charged vectors have a more extended circulation. However, negatively surface-charged vectors are resistant to uptake by targeted cells. Cationic vectors adsorb large amounts of serum proteins during circulation, which mediates the aggregation and degradation of vectors and accelerates the removal of cationic vectors.^90^ In addition, electrostatic interactions between cationic vectors and red blood cells may cause severe hemagglutination and hemolysis.^91^ Therefore, nearly neutral vectors after loading nucleic acid drugs are preferable.

Rigidity is one of the essential mechanical property parameters of nanoparticles, which, to a certain extent, determines nanoparticle in vivo transport behavior. The rigidity can affect the internalization pathway of nanoparticles by cells. Nanoparticles with greater rigidity are more advantageous for cellular uptake, as cell membranes can more easily encapsulate hard nanoparticles than soft ones. However, soft nanoparticles have the propensity to undergo deformation during membrane penetration and rearrange their hydrophobic components, resulting in non-endocytic uptake.^92–94^ Jana et al. modified the surface of AuNPs with various polymers. They discovered that modification of cationic polymers caused nanoparticles to enter the cell via clathrin-mediated endocytosis and be engulfed by endosomes/lysosomes. The modification of non-ionic polymers facilitates the passage of nanoparticles through lipid-raft-mediated uptake, preventing their capture by endosomes/lysosomes and allowing their direct delivery to the cytoplasm.^95^ In addition, nanoparticle rigidity can influence their circulation, biodistribution, and tissue-targeting ability in vivo. Mitragotri et al. examined the cellular uptake, in vivo circulation, biodistribution, and tissue targeting ability of spherical nanoparticles of the same size (200 nm) but with differing rigidity. Compared to the stiffer nanoparticles, the softer nanoparticles (with an elastic modulus of 10 kPa) had a longer circulation time in vivo and more effective targeting in organs such as the spleen, lung, heart, and brain (with an elastic modulus of 3000 kPa). The cellular uptake of harder nanoparticles is greater than that of softer nanoparticles.^96^ Modulating the nanoparticles’ stiffness may therefore improve their in vivo circulation, increase their tissue targeting, and decrease their immune system uptake.^97^

Surface modifications can extend the vectors’ half-life. Stealth polymer coatings, such as PEG, can inhibit the non-specific protein adsorption of the vectors during circulation, thereby extending the half-life.^98,99^ However, PEG triggers the accelerated blood clearance (ABC) phenomenon.^100,101^ After the first dose, PEG stimulates the proliferation and differentiation of B cells responsible for antibody production and cell-mediated immune responses in the spleen, resulting in the generation of anti-PEG IgM.^102,103^ Subsequent systemic doses of PEGylated vectors were rapidly eliminated by Kupffer cells in collaboration with anti-PEG IgM and complement systems.^104–106^ Other polymers with stealth functions have been attempted to extend the half-life of vectors,^107^ such as polyvinyl,^108,109^ polaxamer,^110^ polyglycerol,^111^ poly(amino acid)s,^112,113^ poly(2-oxazoline),^114^ and polyzwitterions. In addition, the unique molecular structure of zwitterionic polymers exhibits excellent resistance to protein adsorption.^115–117^ Polyzwitterions have a pair of opposing charges on each side chain, which form a stable hydration layer through electrostatic-induced hydration.^118^ Compared to the hydration layer formed by hydrogen bonds near the PEG surface, the hydration layer formed by ionic solvation near the polyzwitterion surface is more stable and stays longer. Removing the hydration layer near the polyzwitterion surface thus requires overcoming a higher energy barrier, which is necessary for protein adsorption.^119^ Our laboratory first applied polyzwitterions to the delivery of siRNA in vivo.^120^ Polyzwitterions not only prolonged the half-life of siRNA but also did not stimulate IgM production in the spleen, thus avoiding the ABC phenomenon.^121,122^

In recent years, membranes derived from organisms have been involved in drug delivery in vivo. Zhang et al. coated traditional organic nanoparticles with erythrocyte membranes, thus bypassing the uptake of macrophages and systemic clearance and prolonging the half-life of drugs.^123^ Tasciotti et al. coated conventional inorganic nanoparticles with cellular membranes purified from leukocytes, thereby evading immune system recognition and clearance by reducing opsonization. These bionic vectors significantly extend drug circulation.^124^ Zhang et al. enclosed conventional nanoparticles in the plasma membrane of human platelets.^125^ Such platelet membrane-cloaked nanoparticles facilitated the drug to escape from the uptake of macrophage-like cells, inhibited the activation of the complement system, and reduced the risk of allergy. In addition, these platelet membrane-cloaked nanoparticles retained part of the platelet functions, such as selective adhesion to damaged blood vessels of humans and rodents and enhanced binding to pathogens that adhere to platelets.^125–127^

### BBB

Anatomically, the BBB is a neurovascular unit formed by endothelial cells, pericytes, astrocytes, and other supporting cells of the central nervous system (CNS).^128^ The BBB strictly limits the entry of molecules into the brain through tight junctions between endothelial cells, which protects the CNS while limiting the accumulation of therapeutic drugs in the brain.^129^ The efficient transport of nucleic acid drugs across the BBB to the brain is a primary challenge in gene therapy for brain diseases.^9^

In a healthy state, BBB endothelial cells have tight junctions, with pericytes and astrocytes covering endothelial cells closely, relatively few leukocyte adhesion molecules, and low cross-cellular permeability.^130^ Some diseases can cause BBB dysfunction, which affects the transport of ions, molecules, and cells between the blood and the brain. Ischemic tissue damage, angioedema, and cerebral hemorrhage may result from cerebral vascular lesions and BBB dysfunction following the onset of an ischemic stroke. Therefore, rapid perfusion is necessary to restore the permeability of the blood-brain barrier. Consequently, monitoring the status of BBB leakage in conjunction with MRI, CT, and single-photon emission computed tomography (SPECT) may also provide valuable information for guiding the subsequent treatment plan for brain diseases such as ischemic stroke and cerebral infarction. The BBB is also compromised in patients with brain tumors. Rapid tumor progression stimulates the secretion of vascular endothelial growth factor (VEGF), angiopoietin, and inflammatory cytokines, resulting in abnormal blood vessel growth that may interfere with the BBB’s normal connectivity.^131–134^ Therefore, numerous studies have focused on regulating vascular-related signaling pathways in brain tumor patients.^135,136^ Using miRNA-451 to target CAB39, Zhong et al. prevented the mammalian target of rapamycin (mTOR)/hypoxia-inducible factor-1α (HIF-1α)/VEGF pathway from inhibiting glioma cell growth and invasion.^137^ In some disease states, the integrity of the BBB may be altered, resulting in varying degrees of leakage. However, the BBB is a structure organized by numerous cellular interactions, and numerous factors govern its permeability and function. Crossing the BBB is still one of the most important factors to consider when designing delivery vehicles for nucleic acid drugs used to treat brain diseases.

Passive diffusion cannot cross the BBB for the majority of drugs. Carrier-mediated transport (CMT) is the transport of cargoes across the BBB by specific membrane carrier proteins in an inverse concentration gradient.^138^ CMT facilitates the passage of nucleic acid-based drugs across the BBB by improving brain targeting and uptake. Numerous transporters, including those for amino acids, glucose transporter proteins, neurotransmitters, vitamins, and fatty acids, are located on the BBB, and these transporters supply the brain with nutrients.^139^ Changing certain transporters or their analogs on nanoparticles is an effective method for increasing their BBB permeability. The BBB has a high binding affinity for the large neutral amino acids transporter 1 (LAT1). LAT1 can transport L-tyrosine, L-tryptophan, and L-histidine from the blood to the brain.^140,141^ The protein glucose transporter 1 (GLUT1) is a hexose transporter. The Na^+^ concentration difference drives the transport of D-glucose, L-ascorbic acid, and their derivatives from the blood to the brain via GLUT1.^142,143^ Shi et al. developed a galactose-modified amphiphilic polymeric micelle system that efficiently traversed the BBB by means of glycemic-controlled GLUT1-mediated transport. GLUT1 recirculation was also facilitated by inducing hypoglycemia in mice, which increased GLUT1 expression on the BBB tubular plasma membrane and facilitated the system’s passage across the BBB.^144^

Administration routes can influence drug accumulation in the brain by bypassing the BBB. Although stereotactically guided local administration can deliver drugs almost entirely to the brain lesion, the operation of this invasive administration is complex and cause damage to the CNS. So, local administration is not elaborated on here. Nasal drug delivery is another administration route that bypasses the BBB.^145,146^ The nose is the only location where the brain is in contact with the outside world.^147^ Intranasal administration allows for the direct delivery of drugs to the brain along the olfactory and trigeminal pathways.^148,149^ Transnasal delivery of drugs to the brain must cross the mucosal layer by trans- or paracellular transport.^146^ Chitosan modification enhances the adhesion of the vector to the nasal mucosa, thus allowing more drugs to cross the nose into the brain by increasing the retention time in the mucosa.^150^ In addition, chitosan also serves as a Protein Kinase C inhibitor to temporarily open the tight junctions between epithelial cells and improve the permeability of the mucosa, facilitating the vectors to cross the mucosal layer into the brain.^151,152^ Cell-penetrating peptides (CPPs) are synthesized from cationic amino acids such as arginine or lysine by condensation reactions that enhance cell permeability through electrostatic interactions with cell membranes. CPPs enhance the permeability of nasal epithelial cells, thereby facilitating the delivery of drugs to the brain via the nasal cavity via trans-cellular transport.^153,154^ Receptor-mediated transcytosis (RMT) is also a strategy to enhance cell permeability. L-fucose is widely distributed on the olfactory epithelium of the nasal mucosa. Lectin recognizes and specifically binds L-fucose, enhancing nose-to-brain efficiency via RMT. *Aleuria aurantia* lectin modification facilitated dendrigraft polymer nanoparticles nasal delivery into the brain.^155^ Odorranalectin, the smallest peptide with lectin-like activity, also enhances the efficiency of nose-to-brain transport.^156^ Increasing the maximum single dose without decreasing patient compliance and minimizing nasal mucosal damage should be the priority for future research on nose-to-brain delivery.

Temporarily opening the BBB by physical or chemical approaches can facilitate the delivery of drugs from the periphery to the brain. Focused ultrasound coupled with the intravenous administration of microbubbles (FUS-MB) is a strategy that has been extensively studied to open the local BBB temporarily in recent years.^157^ The drug delivery window created by FUS-MB is about 1~4 h and is recovered after about 24 hours.^158^ FUS-MB can control the drug accumulation position in the brain through the localization of focused ultrasound. Magnetic resonance imaging (MRI) provides precise localization of the FUS-MB. MRI-guided FUS-MB has been used to precisely open the BBB in the tumor^159^ and striatal^160^ regions, which is applied to treating glioma and Parkinson’s disease (PD), respectively. Focused ultrasound increases the local temperature, mechanical effects caused by FUS-MB damage blood vessels, and the opening of the BBB allows the transport of toxic substances to the CNS. So, the primary consideration of FUS-MB is to minimize brain damage by optimizing the FUS parameters.^161^ FUS-MB can control the drug accumulation position in the brain through the localization of focused ultrasound. The ultrashort pulsed laser can also locally open the BBB by a principle like FUS-MB without disturbing the vascular integrity.^162^ Although whole brain hyperthermia can also improve BBB permeability, this crude approach should not be advocated.^163^ Hyperosmotic solutions, such as mannitol,^164^ cause cell contraction by creating an osmotic pressure in the brain microvasculature endothelial cells. The subsequent widening of the tight junctions between endothelial cells increases the permeability of the BBB.^165^ The drug delivery window created by hyperosmotic solutions is about 40 min, and the osmotic pressure returns to normal after 8 hours.^166^ Biological substances (zonula occludens toxin, histamine, bradykinin, Cereport, LipoBridge, and VEGF) and chemical substances (oleic acid, lysophosphatidic acid, and sodium dodecyl sulfate) can also temporarily open the BBB.^167^

Brain microvascular endothelial cell membranes express a variety of receptor transporters such as lipoprotein receptor-related protein 1 (LPR1),^168^ transferrin (Tf),^169^ nicotinic acetylcholine receptor (nAchR),^170^ and low-density lipoprotein receptor (LDLR).^171^ Ligand modifications on the nanoparticle surface can facilitate drug crossing of the BBB via RMT.^172,173^ Most of these ligands are sequence-specific peptides, which is attributed to sequence-specific peptides being synthesized at a low cost to obtain large-scale products. In addition, peptides have reactive groups such as amino, carboxyl, and sulfhydryl groups that can be chemically modified on the surface of nanoparticles in mild reactions. Modification of cysteine on peptides of specific sequences followed by covalent attachment to maleimide-modified nanoparticles by the Michael addition reaction has developed a standard method for vector-modified peptides. Specific-sequence peptides such as T7 (HAIYPRH),^174^ T12 (CGGGTHRPPMWSPVWP),^175^ TBP (GGGHKYLRW),^176^ and B6 (GHKAKGPRK)^177^ have been developed to increase the accumulation of drugs in the brain by specifically binding Tf on the BBB. Specific-sequence peptides such as peptide-22 (Ac–(cMPRLRGC)c–NH_2_)^178^ and unnamed peptides (Acp-(CMPRLRGC)c-NH_2_)^179^ facilitate drug transport from the periphery to the brain by targeting the LDLR. LPR1 is highly expressed on both BBB and glioma cells and is thus the first choice for treating glioblastoma (GBM).^180^ Angiopep-2 (Ang, TFFYGGSRGKRNNFKTEEYC) is the most representative peptide targeting LPR1. Ang, derived from the Kunitz domain of aprotinin,^181^ facilitates drug crossing the BBB and targeting glioma cells and has been widely used in treating GBM.^182–185^ nAchR is a receptor transporter highly expressed in both the BBB and neurons and is thus the preferred choice for CNS diseases such as NDs. RVG29 (YTIWMPENPRPGTPCDIFTNSRGKRASNG), a rabies virus glycoprotein (RVG)-derived peptide containing 29 amino acids,^186^ is the most representative ligand peptide for nAchR.^187–189^ RVG29 modification enables nanoparticles to efficiently cross the BBB and recognize neurons, applying to various CNS disorders, such as major depressive disorder (MDD),^190^ Alzheimer’s disease (AD),^191,192^ PD,^193,194^ Huntington’s disease (HD)^195^ and cerebral ischemia (CI).^196^

Adsorptive-mediated transcytosis (AMT) is another strategy to facilitate drug crossing of the BBB without opening the BBB. CPPs rich in cationic amino acids such as arginine or lysine can enhance BBB permeability by electrostatic adsorption with endothelial cells. The first trans-activator of transcription type CPP was TAT (YGRKKRRQRRR), a cationic peptide derived from human immunodeficiency virus-1 (HIV-1).^197^ Low molecular weight protamine (LMWP, VSRRRRRRGGRRRR), a kind of CPPs, can also improve the permeability of nanoparticles in the BBB.^198^ However, CPPs theoretically enhance the permeability of a wide diversity of cells. Modifying CPPs may promote drug uptake by inappropriate cells after systemic administration, producing side effects. In addition, the positive charges from CPPs may also accelerate the clearance of nanoparticles in circulation. Therefore, applying CPPs in systemic drug delivery should be considered with caution.

The natural ability of certain cells, organelles, and viruses to straddle the BBB has inspired the development of vectors in two aspects. On the one hand, engineered cellular components are employed as vectors, are genetically engineered, modified, or participate in constructing vectors, thereby accessing part of their functions. On the other hand, the physicochemical properties, such as morphology and components of cells, organelles, and viruses, are adopted in vector research and development to impart some of their functions to vectors. During brain inflammation, immune cells such as macrophages and monocytes are recruited through the BBB.^161^ It has been demonstrated that macrophages^199,200^ and neutrophils,^201,202^ acting as “Trojan horses,” can deliver drugs to the brain via the BBB. In addition, macrophages transfected with glial cell line-derived neurotrophic factor (GDNF) can migrate to the brain after systemic administration and almost completely restore motor function in PD mice.^203^ The drug-loaded nanoparticles can be coated by macrophage plasma membranes^204^ or neutrophil-macrophage hybrid membranes,^205^ which facilitates crossing the BBB for treating GBM. Although macrophages facilitate the treatment of brain diseases, their potential CNS toxicity should be considered with caution. Rabies virus is a typical neurotropic virus.^206^ Among the five kinds of proteins that cover the rabies virus, RVG is thought to be the ligand-protein that assists the rabies virus in entering the brain via the BBB.^206^ In addition, the rabies virus has a bullet-like shape with one flat end and one rounded end, with an average diameter of about 75 nm (45~100 nm), an average length of about 180 nm (100~430 nm), and an aspect ratio of approximately 2.4.^207^ This unique shape probably contributes to the uptake of the rabies virus with BBB endothelial cells and neurons. Youn et al. mimic rabies virus size, shape, and surface glycoprotein modification in the preparation of silica-coated gold nanorods, which play a crucial role in facilitating the penetration of nanoparticles into the brain from the periphery and their specific uptake by neurons.^208^ Metal-organic frameworks that adapt the size, bullet shape, and RVG coating of rabies virus were proved to have consistent effects with rabies virus-inspired gold nanorods.^209^

Nature-inspired vectors have shown promise in straddling the BBB but are still mainly in the “fetch-to-use” research stage. Do drugs affect the function and activity of the cells that serve as vectors? Extracting cellular components, such as plasma membranes, obtains complex systems. Are the molecules of these intricate systems well-defined and under control? Do vectors derived from organisms induce adverse effects such as immune response and inflammation? Exist strict criteria for identifying such organism-derived vectors? Have they the capacity for mass production? Exploring the mechanisms by which these cells and viruses cross the BBB may be a more appropriate strategy to guide nature-inspired vector development. Notably, the high permeability of these cells and viruses across the BBB is likely to be mediated by multiple factors synergistically, which will require a concerted effort by researchers in multiple fields, such as cell biology, microbiology, and biomechanics. But this promises to provide a paradigm for nature-inspired drug vector development for brain diseases and extends beyond nature. An ideal nature-inspired vehicle would have no nature itself but exhibit nature’s wisdom throughout.

### Cellular uptake

Nucleic acid drugs exert their therapeutic effects intracellularly. However, the high density of negative charges in the nucleic acid drugs themselves does not allow them to be taken up by the cells. Therefore, the efficient transport of nucleic acid drugs into the cells is an essential function of the vectors. In addition, precise targeting of the diseased cells is necessary for the vector, which allows for efficient therapeutic effects with less dose and reduces side effects. Therefore, vectors address two critical issues in cellular uptake: precise targeting and efficient uptake. The main routes of cellular uptake are phagocytosis, macropinocytosis, clathrin-mediated endocytosis (CME), caveolae-dependent endocytosis, clathrin/caveolae-independent endocytosis, and passive uptake. The molecular mechanisms of these uptake routes can be referred to Reviews by Rothen-Rutishauser et al.,^210^ Mahmoudi et al.,^211^ and Parton et al.^212^ and will not be described in detail here.

The physicochemical properties of the vectors, such as size, shape, surface charge, and roughness, affect cellular uptake. The vectors’ size mainly affects cellular uptake through uptake routes and rates. Large vectors (>500 nm) are typically only taken up by phagocytes via phagocytosis and micropinocytosis routes.^213^ Among vectors smaller than 200 nm, small vectors (around 60 nm) tend to be taken up via the caveolae-dependent endocytosis pathway. The larger vectors (about 120 nm) prefer to be taken up through the CME route.^210,214,215^ The cellular uptake route also changes with increasing size after the vectors are aggregated.^216^ The size of the vectors affects the rate of cellular uptake. However, it should be clear that the regularity of the effect of vector size on the rate of cellular uptake is uncertain. Larger sizes may either accelerate^217^ or slow down^218^ cellular uptake. Large and small-size vectors may also stimulate each other’s cellular uptake.^219^ Therefore, it is also relevant to the screen for the optimal vector size, but it should also be clear that this optimal size applies only to the vector and cells used in the screen. Vácha et al. compared the cellular uptake between spherical particles and spherocylinder (cylinder with hemispherical caps at both ends) particles with the same diameter and ligand coating by molecular dynamics simulation.^220^ Under non-ATP-driven conditions (passive endocytosis), the endocytosis efficiency of spherocylinder particles was higher than that of spherical particles. In addition, passive endocytosis of particles with sharp edges was inhibited. The authors explained the simulation results by the membrane elasticity theory. Spherocylinder particles with smaller mean curvature were favored to be wrapped by the cell membrane. The maximum local curvature influenced the limiting step in entering the cell by separating the membrane molecule-encapsulated particle from the cell membrane. Two hemispherical caps cause the same maximum local curvature between spherocylinder particles and spherical particles. So, there was no difference in separating these two particles with different shapes. Therefore, with the advantage of cell membrane encapsulation, spherocylinder particles showed better endocytosis efficiency. However, the molecular dynamics simulation model was overly simplified compared to the natural process of cellular endocytosis. Endocytosis results in A375 human melanoma cells by Tang et al. were consistent with the simulation,^221^ whereas the results in Hela by Chan et al. were inconsistent with the simulation.^222^ The positive surface charges facilitate cellular uptake, while the negative surface charges inhibit cellular uptake. However, as mentioned previously, cationic vectors’ clearance in circulation and toxicity should be carefully considered during systemic administration. Topography, such as roughness, also affects endocytosis efficiency. Roughness (localized protrusion or depression) affects the surface effect, determining the intensity of the interaction between the vectors and cell membranes. The roughness facilitates cellular uptake through adsorption-mediated endocytosis (AME) by increasing the contact area with cell membranes and reducing the repulsive forces with cell membranes, such as electrostatic and hydrophilic.^223^ In addition, roughness also increases the specific surface area of particles, which is also beneficial for endocytosis. Notably, roughness may also enhance protein adsorption of nanoparticles in circulation, which accelerates the clearance of vectors.^224^

Electrostatic interactions of CPPs with anionic proteoglycans or phospholipids on the cell membrane mediate cellular uptake via direct translocation or endocytosis.^225^ It is important to note that CPPs do not interact exclusively with specific proteoglycans or phospholipids. So, the cellular uptake promoted by CPPs is non-specific.^226,227^ CPPs exhibit advantages in the cellular uptake in vitro, especially in low permeability cells, and are thus applied to in vitro drug-induced cells for cellular therapy, such as low permeability neural stem cells (NSCs). NSCs aggregate into spheres when cultured in vitro. Therefore, penetrating the drug into the NSCs inside the cell spheres is challenging. Our laboratory has modified the CPP containing only four amino acids (KRKR) on the surface of siRNA/chem vectors to facilitate the in vitro uptake of drugs by NSC spheres. siRNA/chem-induced NSCs were then employed for the cellular treatment of AD.^228^

Specific receptors on cell membranes provide a pathway for vectors targeting diseased cells. Ligand-modified vectors are promising for precisely delivering drugs to diseased cells via receptor-mediated endocytosis (RME), thereby reducing doses and side effects. These ligands are mainly classified as small molecules and peptides. Folic acid, a typical ligand with a small molecule, is applied to treating brain tumors by binding specifically to the highly expressed folate receptor in the tumor cell membrane.^229–231^ In addition, mazindol^232^ and sertraline^233^ bind specifically to the dopamine transporter (DAT) and the serotonin transporter (SERT) on the cell membrane surface of dopaminergic neurons and 5-hydroxytryptamine (5-HT) neurons, respectively, thereby being employed in PD and depression, respectively. Mannose-modified vectors can bind to mannose receptors on microglia, thus assisting in treating NDs.^234,235^ We have already described the peptides Ang and RVG29. In addition, DAG (CDAGRKQKC) specifically binds to connective tissue growth factor (CTGF), which is highly expressed in the brain of AD patients and promotes the cellular uptake of the vectors in AD lesions.^236^ Tet1 (HLNILSTLWKYR) has a high affinity for trisialoganglioside (G_T1b_) on neurons, enabling the treatment of NDs.^237,238^ The cyclic RGD sequence (cRGD), which is widely used in the treatment of tumors, binds specifically to α_v_β_3_ and α_v_β_5_ integrins on the endothelial cells of angiogenic tumor vessels to facilitate vector targeting of brain tumors.^239–241^

Nature also inspires the design of vectors. Exosomes act as vectors of intercellular molecular information, delivering cargo molecules involved in physiological and pathological processes from parent cells to recipient cells.^242^ Therefore, exosomes possess a natural cellular uptake advantage for recipient cells. Batrakova et al. loaded macrophage-derived exosomes with catalase. The catalase-loaded exosomes accumulated efficiently in neurons and microglia of the brain and exhibited effective neuroprotective effects.^243^ Rameshwar et al. demonstrated that microvesicles mediated intracellular communication between mesenchymal stem cells (MSCs) and GBM cells. MSCs specifically delivered anti-miR-9 to GBM via exosomes, reversing multidrug transporter expression and thereby sensitizing GBM to temozolomide (TMZ).^244^ The biomimetic camouflage of leukocytes may recognize and bind to the tumor endothelium in an active and non-destructive manner, thus avoiding the uncertainty of tumor treatment caused by the individual variability of traditional enhanced permeation and retention (EPR) effects.^124^ We mentioned that neurons specifically and efficiently took up rabies virus-mimicking nanoparticles. In addition, the nanoscale surface roughness of neurotropic viruses such as the Japanese encephalitis virus, West Nile virus, and measles virus improves non-specific binding for cellular uptake.^245–248^ Our laboratory facilitated BBB penetration and neuronal uptake of the vectors by modifying plenty of small particles (3~5 nm) on the surface of large spherical particles (~40 nm) to mimic virus surface topography.^249^

### Endosome/lysosome barriers

Abundant nucleases in the endosome/lysosome degrade nucleic acid drugs. Therefore, overcoming the endosome/lysosome barriers is essential for nucleic acid drugs. There are three main ways to overcome the endosome/lysosome barrier: passively bursting the endosome/lysosome, actively inducing endosome/lysosome degradation, and bypassing the endosome/lysosome pathway during endocytosis.

Bursting the endosome/lysosome by the proton sponge effect is the most common endosome/lysosome escape strategy. Polymers containing groups such as primary, secondary, and tertiary amines can adsorb hydrogen protons, thereby significantly increasing the osmotic pressure between the inside and outside of the endosome/lysosome. The endosome/lysosome then swells until it ruptures by absorbing water, thus releasing the vectors. Polyethyleneimine (PEI), with a high concentration of primary, secondary, and tertiary amines, is the most typical polymer with a proton sponge effect and is widely used to deliver nucleic acid drugs.^250–252^ Nevertheless, the toxicity of PEI is a matter that must be considered.^253^ In addition, cationic polymers such as poly(2-(dimethylamino)ethyl methacrylate) (PDMAEMA) with tertiary amines,^254,255^ polylysine with primary amines,^192,256^ poly(amidoamine)s (PAAs) with secondary and tertiary amines,^257,258^ poly (L-histidine) (PHis) with imidazole group,^259,260^ poly(arginine) with primary and secondary amines,^261^ and poly(β-amino esters) (PBAE)^262,263^ with tertiary amines have also been applied for loading nucleic acid drugs and promoting endosome/lysosome escape via the proton sponge effect. RVG29-9R (YTIWMPENPRPGTPCDIFTNSRGKRASNGGGGRRRRRRRRR), a peptide with the addition of nonamer containing nine arginine residues to the carboxyl terminus of RVG29, can load nucleic acid drugs and facilitate endosome/lysosome escape via the proton sponge effect without reducing the function of RVG29.^188,264^

Vectors can also actively interact with endosome/lysosome to induce their degradation. Our laboratory synthesized lipids DSPE to initiate the zwitterionic monomer carboxybetaine methacrylate (CB-MA) polymerization. The phospholipid-modified polyzwitterions were named DSPE-PCB. DSPE-PCB had pH-sensitive intramolecular charge transition properties. DSPE-PCB then participated in the construction of liposomes and siRNA encapsulation. Under acidic conditions in the endosome/lysosome, the carboxylate radical in DSPE-PCB adsorbed large amounts of H^+^, which switched DSPE-PCB from neutral to positively charged. The cationic DSPE-PCB in siRNA-loaded liposomes interacted electrostatically with negatively charged lipids on the endosome/lysosome membrane, which mediated the co-degradation of liposomes and endosome/lysosome.^120–122^ The amphiphilic polymer TBD-PEG-N_3_ was synthesized by Liu et al. by modifying the hydrophobic photosensitizer TBD with PEG. TBD-PEG-N_3_ subsequently self-assembled into PNPs and conjugated with DBCO-modified ASOs by click chemistry. The photosensitizer TBD is designed using methoxy-substituted tetraphenylethylene as the electron donor, benzothiadiazole as the auxiliary acceptor, and a dicyanovinyl group as the real electron acceptor. The photosensitizer produced large amounts of ^1^O_2_ under light irradiation, which disrupted the endosome/lysosome structure.^265^ Interestingly, Liang et al. prepared a proton-driven transformable nanovaccine. The authors coupled a pyrene-conjugated d-peptide (PDP) to a functional polymer. The PDP-conjugated functional polymer p(OEGMA-DMAEMA)-*b*-p((MAVE)-(MAVE-PDP)) was self-assembled with the antigenic peptide into a spherical nanovaccine. Under physiological conditions (pH 7.4), the nanovaccines were spherical with a diameter of approximately 100 nm. After internalization, the acidic environment of the late endosome (pH 5.6) stimulated the release of PDP. The released PDP was re-assembled into sheet-like particles of 5000~8000 nm, thus disrupting the structure of the endosome.^266^

Cellular uptake pathways that bypass the endosome/lysosome route have recently attracted much attention. Two kinds of exosome/polymer hybrid nanoparticles have been prepared in our laboratory for nucleic acid-chemo drug loading and PD treatment. We demonstrated that exosome encapsulation could mediate the uptake of vectors via membrane fusion, thus bypassing the endosome/lysosome route.^194,267^ Synaptic vesicles are lipid bilayer structures with a 40~100 nm diameter. Synaptic vesicles release neurotransmitters and hormones via fusion with the plasma membrane.^268,269^ Soluble N-ethylmaleimide-sensitive factor attachment protein receptors (SNAREs) are vital proteins that mediate the fusion of synaptic vesicles with the plasma membrane.^270,271^ Our laboratory mimicked the membrane fusion function of Synaptic vesicles by inserting a transmembrane segment syb (TMS-syb), derived from SNAREs of synaptic vesicles, into the artificial lipid bilayer. The results showed that TMS-syb facilitated the neuron uptake of the vector via membrane fusion, thereby avoiding the endosome/lysosome routes.^272^ Tasciotti et al. coated plasma membranes purified from leukocytes on nanoporous silicon particles. The leukocyte-derived plasma membrane coating mediates the vector’s contact directly with the cytoplasm via membrane fusion, thus bypassing the endosome/lysosome pathway.^124^ You et al. found that pardaxin (HGFFALIPKIISSPLFKTLLSAVGGSAVGSALSSGGQE), a peptide consisting of 33 amino acids, was able to mediate the cellular uptake of the vector through the endoplasmic reticulum pathway rather than the endosome/lysosome pathway.^273–275^ After uptake by caveolin-mediated endocytosis, pardaxin-modified liposomes moved along the cytoskeleton and were targeted to the endoplasmic reticulum.^276^ This intracellular transport process was similar to that of the simian virus,^277,278^ which not only avoided the degradation of nucleic acid drugs by nucleases in the endosome/lysosome but also facilitated the access of the pDNA or CRISPR/Cas9 system to the nucleus for gene therapy.^276,279,280^

### Release of nucleic acid drugs

The cytoplasm or the cell nucleus is the site where nucleic acid drugs work. Most vectors are too large to enter the nuclei. Besides, nucleases in the endosome/lysosome degrade prematurely released nucleic acid drugs. In addition, the potential side effects of nucleic acid drugs can be reduced if they are released in the cytoplasm of the diseased cells. Therefore, the cytoplasmic release of nucleic acid drugs must be considered in the vector design.^281^ The direct release of nucleic acid drugs into the cytoplasm via membrane fusion is straightforward and efficient. The mechanism of different vectors releasing nucleic acid drugs through membrane fusion is similar, so it will not be discussed here.

The acidic microenvironment in the endosome/lysosome can serve as an endogenous stimulus to promote the release of nucleic acid drugs. Our laboratory-prepared polyzwitterion-based liposomes could mediate the co-degradation of liposomes and endosomes in the endosome/lysosome through the interaction of pH-triggered liposome membranes with endosome/lysosome membranes, resulting in the release of nucleic acid drugs while the endosome/lysosome escaped.^120–122^ Dong et al. synthesized PCBP-(DPAx-co-DMAEMAy)-PG, tri-block pH-sensitive polycations, assembled them into micelles, and electrostatically adsorbed siRNA. Under acidic conditions, DPA switched from hydrophobic to hydrophilic due to protonation, enabling micelle degradation and the release of siRNA. Interestingly, the initial pH-triggered disassembly point can be tuned by changing the DPA/DMAEMA ratio, thus expanding the applications of the vectors.^282^ Shuai et al. synthesized pH-sensitive amphiphilic block cationic copolymers mPEG-bPEI-PAsp(DIP-BZA) and self-assembled them into micelles. Similar to the work of Dong et al. under acidic conditions, the protonation-mediated hydrophobic-to-hydrophilic transition of PAsp(DIP) led to the degradation of the micelles, which released siRNA.^283^

Glutathione (GSH) is specifically overexpressed in the cytoplasm of brain tumors.^284^ GSH can facilitate the release of nucleic acid drugs by mediating the breakage of disulfide bonds through reductive effects. Dai et al. synthesized GSH-sensitive amphiphilic block copolymers, PEG-*b*-PMPMC-g-PTX (PMP). In PMP, the hydrophobic block was formed by the polymers covalently linking hydrophobic paclitaxel (PTX) through disulfide bonds. PMP, anionic siRNA, and cationic Py-TPE were self-assembled into micelles (Py-TPE/siRNA@PMP) by electrostatic and hydrophobic interactions. GSH in the tumor cytoplasm stimulated the PTX release by cleaving the disulfide bonds, transforming the PMP’s hydrophobic blocks into hydrophilic ones. The micelles then degraded and released siRNA.^285^ Sun et al. prepared disulfide cross-linked cationic dextrin nanogels (DNGs) and adsorbed siRNA. GSH degraded the DNGs by cleaving the disulfide bond, thereby releasing siRNA.^286^ Ding et al. pioneeringly designed a DNA lock to encapsulate siRNA and doxorubicin (DOX). The DNA lock contained disulfide bonds as its lock cylinder. GSH was the key to opening this DNA lock by cleaving the disulfide bonds, which mediated the release of siRNA and DOX.^287^ Interestingly, Shen et al. synthesized a GSH-sensitive polymer PADDAC without disulfide bonds. PADDAC contained quaternary amines and a *p*-dinitrophenyl moiety. Quaternary amines contributed positive charges to PADDAC. The *p*-Dinitrophenyl moiety provides PADDAC with GSH-sensitive properties. The thiol of GSH attacked the *p*-dinitrophenyl moiety, which cleaved the ether bond of PADDAC, caused the formation of phenol anions on PADDAC, and triggered the elimination of *p*-quinone methides. PADDAC was thus transformed into PDEAEA. PDEAEA hydrolyzed to an anionic polyacrylic acid via self-catalysis. The DNA was released by the conversion from electrostatic adsorption to electrostatic repulsion.^288^ It should be noted that the *p*-dinitrophenyl moiety is sensitive only to thiols on GSH but not other thiols.^289,290^ Shi et al. synthesized Cas9/sgRNA nanocapsules with a GSH-responsive release function by in situ free-radical polymerization. GSH cleaves disulfide bonds, resulting in nanocapsule degradation and high-performance PLK1 gene editing.^291^

Various intracellular enzymes are also ideal endogenous stimuli for drug release. Esterase is a hydrolytic enzyme that is widely present in the cytosol. Esterases usually mediate the release of chemical drugs by catalyzing the hydrolysis of ester bonds. Grinstaff et al. synthesized a series of amphiphiles containing benzyl esters at their ends and used them to prepare lipoplexes loaded with DNA or siRNA. Intracellular esterases could mediate the release of DNA or siRNA by catalyzing the hydrolysis of benzyl esters.^292^ Shen et al. synthesized cationic polymers containing quaternary amines with *p*-acetyloxybenzyl groups (PQDEA). The cationic PQDEA formed nanoparticles with the anionic DNA by electrostatic interaction. PQDEA was susceptible to hydrolysis to PDEA with tertiary amines by esterases. PDEA was further self-catalytically hydrolyzed to anionic polyacrylic acid, which released DNA by electrostatic repulsion.^293^ Cathepsin B is highly expressed in the endosome/lysosome.^294^ Our laboratory synthesized amphiphilic block cationic copolymers, T-PA-G-SA. Hydrophobic drug salsalate was linked to the polymer via a sequence-specific peptide (GFLG). T-PA-G-SA participated in the assembly of cationic PNPs and loaded siRNA. High expression of cathepsin B in endosome/lysosome catalyzed the hydrolysis of GFLG, releasing the hydrophobic drug salsalate and mediating the degradation of PNPs, thereby releasing siRNA.^238^ Proteases can catalyze the hydrolysis of amide bonds. Dopamine-modified cationic polylysine was synthesized in our laboratory and modified on the surface of superparamagnetic iron oxide nanoparticles (SPIONs). The hybrid complexes loaded CRISPR plasmids by electrostatic adsorption. The protease reduced the positive surface charges on the hybrid complexes by catalyzing the hydrolysis of the polylysine, which released the CRISPR plasmids.^192^

Reactive oxygen species (ROS) are elevated in tumors and NDs and thus have been used to stimulate the release of drugs. Shen et al. synthesized positive-to-negative charge-reversal polymers with phenylboronic acid (B-PDEAEA). The cationic B-PDEAEA and anionic DNA were self-assembled into nanoparticles by electrostatic adsorption. Phenylboronic acid on B-PDEAEA was susceptible to degradation by ROS, which converted B-PDEAEA to PDEAEA. PDEAEA self-catalytically hydrolyzed to anionic polyacrylic acid, releasing anionic DNA by electrostatic repulsion.^295^ Our laboratory synthesized a ROS-responsive cationic polymer PCB-Se-Se-Sim. We first synthesized polyzwitterions PCB. Subsequently, hydrophobic simvastatin was covalently attached to some of the blocks of the PCB via diselenide bonds. The simvastatin-modified blocks were cationic hydrophobic blocks, and the blocks without simvastatin were zwitterionic hydrophilic blocks. The cationic PCB-Se-Se-Sim was self-assembled as micelles by hydrophobic interaction and electrostatically adsorbed ASOs. ROS cleaved the diselenide bonds, degraded the micelles, and released the ASOs.^296^

## Engineering carriers loaded with a single nucleic acid drug for brain disease treatment

**Fig. 3:**
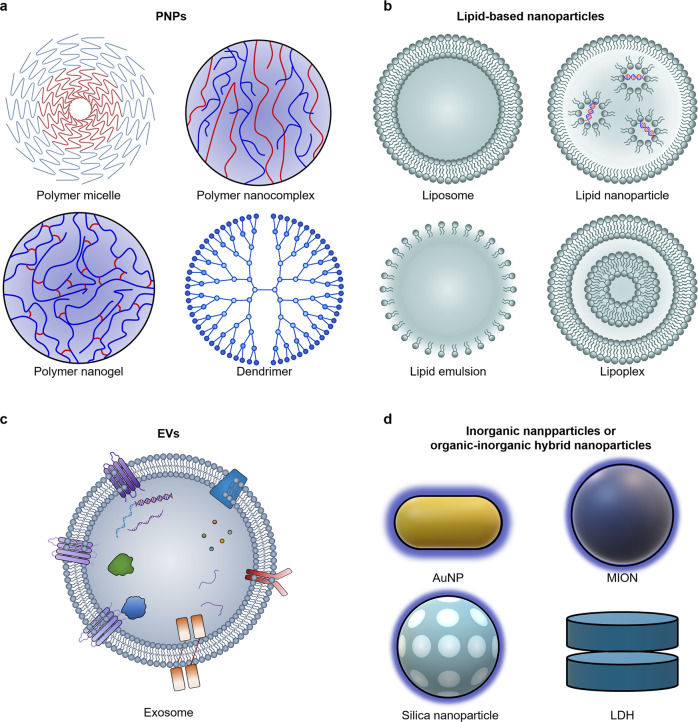
Schematic illustration of classes of nucleic acid drug vectors. PNPs have the advantages of low synthesis difficulty, high chemical structure tunability, simultaneous multifunctionality, and the disadvantages of the tendency to agglomerate and potential toxicity. Lipid-based nanoparticles have the advantage of simple preparation, structural tunability, and interaction with bio-membranes and the disadvantage of low drug loading efficiency and tendency to be cleared by the liver. EVs are natural vectors for cell-to-cell delivery of biomolecules and combine certain therapeutic properties. However, EVs are difficult to isolate, extract, drug load, and modify with low yields and suffer from heterogeneity. Inorganic nanoparticle-based nucleic acid drug vectors have the advantages of homogeneous particle size, shape, surface potentials, highly tunable size and shape, and possible medical imaging signals and the disadvantages of toxicity and solubility. Nucleic acid drug conjugates (not shown) have the advantage of almost absolute homogeneity, high stability, low non-drug component content, and less restricted administration routes and the disadvantage of less functionality and low endosome/lysosome escape efficiency

Several vector types have been developed for brain delivery of nucleic acid drugs and disease therapy, such as PNPs, lipid-based nanoparticles, lipid/polymer hybrid nanoparticles (LPNPs), conjugated nucleic acid drugs, inorganic nanoparticles, organic-inorganic hybrid nanoparticles, and EVs (Fig. 3). We will present these vectors’ design, preparation, and modification in the construction process to overcome the physiological barriers in the delivery and release of nucleic acid drugs. We also present the types and targets of nucleic acid drugs for different brain disease pathologies.

### PNPs

PNPs, broadly speaking, are nanoparticles formed by interactions such as electrostatic, hydrophobic, and/or hydrogen bonds between polymers and nucleic acid drugs. The polymers in PNPs can be one or multiple. The properties of the polymers themselves have a more significant impact on the performance of PNPs than the preparation methods of PNPs. Polymers have three prominent advantages: low synthesis difficulty, high chemical structural tunability, and accessibility to multifunctional properties. Firstly, various polymers can be synthesized by well-established methods such as radical polymerization, reversible addition-fragmentation chain transfer polymerization (RAFT), atom transfer radical polymerization (ATRP), Michael addition reaction, and ring-opening polymerization. Secondly, the polymers’ repeat unit, main chain structure, block type, and degree of polymerization can control their properties. Furthermore, polymers can be imparted with multiple functions by modifying the end groups and/or repeating units of polymers with suitable functional molecules and/or covalently linking multiple polymers through suitable linkers, which is exceptionally profitable for vectors to overcome various physiological barriers. Therefore, we would focus on the control and functionalization of different polymers to explain how PNPs load nucleic acid drugs and overcome the multiple physiological obstacles during their delivery processes.

Cationic polymers form PNPs by electrostatically adsorbing anionic nucleic acid drugs, and PEI is a representative cationic polymer. PEI with a large number of primary, secondary, and tertiary amines has a high density of positive charges, thus enabling efficient loading of nucleic acid drugs and endosome/lysosome escape through the proton sponge effect. Lin et al. enhanced the uptake and endosome escape of ASOs in glioma cells by electrostatic adsorption of anti-miR-125b ASOs by PEI.^297^ miR-125b expression was upregulated in GBM cells and exerted oncogenic effects in GBM cells by promoting cell proliferation and inhibiting apoptosis.^298^ These PNPs inhibited the proliferation of glioma cells by downregulating miR-125b. The α-synuclein gene (SNCA) has been proven to be a key responsible gene in many PD genetic studies. Overexpression of SNCA leads to the formation of α-synuclein aggregates in dopaminergic neurons, which degenerate substantia nigra (SN) dopaminergic neurons and symptoms of PD.^299,300^ Richter et al. employed PEI to electrostatically adsorb siSNCA. After stereotaxic-guided intracerebroventricular (ICV) injection, siRNA-loaded PNPs downregulated α-synuclein expression and improved PD symptoms.^301^ Chen et al. found that polylysine-modified PEI (PLys-PEI) significantly enhanced the transfection efficiency of nucleic acid drugs and reduced the toxicity of PNPs compared to PEI. The authors loaded VEGF pDNA with PLys-PEI. After stereotaxic-guided brain injection, VEGF pDNA-loaded PNPs were efficiently transfected in neurons, exhibiting the potential of PD gene therapy.^302^

The excessive positive charges of PEI accelerate the clearance of the vectors in circulation, which limits the systemic administration of PEI-based PNPs. Price et al. then modified PEG on PEI (PEG-PEI) to improve the stealth performance of the vectors in circulation and thus not be cleared. By temporarily opening the BBB via MRI-guided FUS (MRgFUS), the intravenously injected vectors delivered pDNA to the brain and achieved efficient transfection.^303^ Garg et al. enhanced vector accumulation and penetration in brain tumors by transforming the shape of PEI-based PNPs.^304^ The authors polymerized PEI and dixylitol diacrylate (dXYdA) monomers by the Michael addition reaction (PdXYP). The PdXYP then formed spherical PNPs with siRNA. Subsequently, dXYdA served as a cross-linker to aggregate spherical PNPs into chained PNPs by the Michael addition reaction. Intravenous injection of chained PNPs enhanced siRNA accumulation and penetration in brain tumors compared to spherical PNPs. Anti-serine hydroxymethyltransferase 1 (SHMT1) siRNA downregulated the de novo DNA biosynthesis pathway in tumor cells,^305^ induced GBM apoptosis,^306^ and significantly reduced tumor size in GBM model mice. Cho et al. modified RVG29 and mannitol on PEI to enhance the targeting and permeability of PNPs to the BBB and neurons, respectively.^170^ PNPs loaded with siRNA significantly downregulated the expression of β-site amyloid precursor protein cleaving enzyme 1 (BACE1). BACE1 is the primary and rate-limiting enzyme for amyloid-β (Aβ) production.^307,308^ Abnormal accumulation of Aβ, especially its 42-amino acid isoform (Aβ42), is a prominent pathological feature of AD.^309–311^ Therefore, these PNPs have the potential to treat AD.

The excessive positive charges of PEI also prevent the release of nucleic acid drugs that are adsorbed on PEI-based PNPs. Lee et al. overcame the challenge of nucleic acid drug release by introducing disulfide bonds in PEI-based PNPs. They thiolated the PEI, cross-linked the thiolated PEI by disulfide bonds, and modified the cross-linked PEI with RVG29. Subsequently, the cross-linked PEI was loaded with miR-124a and administered via the tail vein. The intracellular reduction environment, such as GSH, could mediate the degradation of PNPs and the release of miRNAs by cleaving disulfide bonds.^312^ miR-124a is a neuron-specific miRNA with a potential function in promoting neurogenesis, and its expression level gradually increases during neuronal development.^313–315^ Therefore, these PNPs have the potential for gene therapy of CNS diseases. Jiang et al. also cross-linked branched oligoethylenimine (OEI) via disulfide bonds to achieve GSH-stimulated degradation of PNPs. Interestingly, the authors designed a cyclic polypeptide iNGR (CRNGRGPDC, with cysteine residues at both ends linked by disulfide bonds to form a cyclic polypeptide), and modified it on the cross-linked OEI.^316^ iNGR had three features: a vascular homing motif, a protease recognition site, and an R/KXXR/K tissue penetration motif (CendR motif). The iNGR-guided vectors accumulated in the tumor vasculature by CD13^317,318^ and were recognized and cleaved by proteases on the vascular surface,^319^ exposing the R/KXXR/K motif (RNGR) inside the C-terminus. The CendR motif promotes vector uptake by GBM cells via cell surface neuropilin-1 (NRP-1) receptor-mediated endocytosis.^320^

The toxicity of PEI limits its application in nucleic acid drug delivery. Fluorinated PEI enables effective gene transfection at extremely low N/P ratios, thereby reducing the toxicity of the vectors. Zhang et al. synthesized fluorinated PEI by modifying heptafluorobutyric anhydride on PEI and prepared a core-shell structure of polymer nanoparticles loaded with pDNA. pDNA was loaded by fluorinated PEI as the core of PNPs (PF/pDNA), followed by electrostatic adsorption of the polymer P2-PEG-HSA-AA as the shell (PHSA@PF/pDNA).^321^ P2 (CGRILARGEINFK) is a peptide that binds specifically to neural cell adhesion molecules (NCAM), which are highly expressed in glial cells and BBB endothelial cells.^322–324^ The modification of human serum albumin (HSA) enhanced vector stability and was resistant to serum protein adsorption in circulation, thus avoiding clearance. *cis*-Aconitic anhydride (AA), which can provide negative charges with HSA, facilitated the adsorption of P2-PEG-HSA-AA on the PF/pDNA surface (PHSA@PF/pDNA). AA was degraded via amide bond breakage under acidic endosome/lysosome conditions, reducing the electrostatic interaction between the shell and core of PHSA@PF/pDNA, and thus exposing PF/pDNA. Therefore, HSA modification overcame the physiological barriers in circulation. P2 modification overcame the physiological barriers in the BBB and cellular uptake. AA modification overcame the physiological obstacles to endosome/lysosome escape and drug release. pDNA is an efficiently expressed triggering receptor on myeloid cells 2 (TREM2) in brain microglial cells. TREM2, an immunoglobulin superfamily transmembrane receptor expressed only by microglia in the brain, regulates phagocytosis, inhibits inflammatory signaling, and is essential in maintaining microglia proliferation and survival.^325–327^ Overexpression of TREM2 promotes microglia phagocytosis and suppresses neuroinflammation.^328–330^ Therefore, PHSA@PF/pDNA has therapeutic potential in NDs such as AD.

PDMAEMA with tertiary amine is also commonly used as a cationic polymer for loading nucleic acid drugs. PDMAEMA is generally synthesized by free radical polymerization of the DMAEMA monomer. Zhan et al. synthesized the amphiphilic block copolymer PEG-PDMAEMA. Subsequently, the authors modified the peptides CGN (d-CGNHPHLAKYNGT) and QSH (d-SHYRHISPAQV) on PEG-PDMAEMA, respectively. CGN exhibited a higher affinity for BBB endothelial cells and facilitated the vector crossing of the BBB. QSH specifically binds Aβ1~42 and thus targets AD neurons. CGN-PEG-PDMAEMA, QSH-PEG-PDMAEMA, and siBACE1 formed PNPs through electrostatic interaction. PEG promoted the prolonged circulation of the vectors. CGN and QSH assisted the vectors in crossing the BBB and targeting AD neurons. Tertiary amines of PDMAEMA assisted the vectors in escaping from the endosome/lysosome through the proton sponge effect.^331^ Zhan et al. replaced QSH with Tet1 (HLNILSTLWKYR), another peptide targeting neurons, and achieved similar results.^332^

PEI and PDMAEMA, despite their excellent nucleic acid drug loading and endosome/lysosome escape abilities, are both toxic due to their resistance to degradation. Therefore, degradable cationic polymers have been developed. PBAE is one of the representatives. Most of the PBAE contains a large number of ester bonds in the backbone, so they can be hydrolyzed into small molecules by esterases in vivo and then metabolized, thus being less toxic. Green et al. prepared a series of PBAEs and investigated the effects of their molecular weight and hydrophobicity on the transfection of siRNA, linear DNA, and circular DNA, respectively. Briefly, longer PBAEs tend to have higher transfection efficiency of DNA. siRNA requires PBAEs to have the ability to release drugs as soon as they enter the cytoplasm. These PBAE-based PNPs exhibited the potential for GBM therapy.^333^ Hanes et al. delivered PBAE-loaded pDNA to the brain by intracranial perfusion and observed transfection of pDNA.^334^ Hinojosa et al. then delivered bone morphogenetic protein 4 (BMP4) pDNA to human adipose-derived mesenchymal stem cells (hAMSCs) by PBAE.^335^ With the benefits of MSCs’ low immunogenicity and tumor tropism,^336^ hAMSCs bypassed the BBB and migrated toward GBM after intranasal administration. hAMSCs-expressed BMP4 decreased the tumorigenicity of brain tumor initiating cells.^337,338^ Green et al. introduced disulfide bonds in the backbone of PBAE, enabling PBAE to be reduced to small molecules by GSH, which facilitated the release of siRNA from the vectors in GBM cells.^339,340^

Poly(amino acid)s are another class of degradable cationic polymers. Poly(amino acid)s are peptide-like polymers formed by amino acid monomers through amide bonds and can be hydrolyzed by proteases in vivo. Vicent et al. synthesized a series of poly-L-ornithine (PLO)-based polymers and loaded anti-death receptor 6 (DR6) siRNAs.^341^ These PNPs can escape from the endosome/lysosome and release siDR6 via a primary amine-mediated proton sponge effect, thereby downregulating DR6 expression. The silencing of DR6 promotes remyelination and axon regeneration, which is expected to treat CNS disease.^342^ Kataoka et al. synthesized GLU-PEG-PLys(MPA/IM) by co-modifying 3-mercaptopropyl amidine (MPA) and 2-thiolaneimine (IM) on the side chains of PEG-PLys and modifying glucose (GLU) on the end groups of PEG-PLys.^343^ GLU-PEG-PLys (MPA/IM) has three types of side chains: with amine, with MPA, and with IM. the side chains without MPA and IM provided positive charges to adsorb ASOs. The side chains with MPA provided hydrophobic interaction. The side chains with IM cross-linked the polymers through disulfide bonds. GLU assisted vectors across the BBB via active translocation of GLUT1 from the apical to the basal side of the BBB endothelium. Side chains with amine-assisted endosome escape via a proton sponge effect. GSH degraded the vectors by cleaving disulfide bonds, which promoted the release of anti-metastasis-associated lung adenocarcinoma transcript 1 (MALAT1) ASOs and downregulated MALAT1 expression.

Anionic poly(amino acid)s have also participated in constructing nucleic acid drug vectors. Cheng et al. used a CPP with a linear and rigid rod-like structure, PVBLG-8, to electrostatically adsorb siRNA. To attenuate its rigidity, the authors used the anionic poly(L-glutamic acid) (PLG) with a flexible backbone as a physical cross-linker and stabilizer to generate sufficient molecular tangles with PVBLG-8 by electrostatic interaction, which encapsulated the siRNA in a polymeric network (PVBLG-8/siRNA/PLG). The surface of PVBLG-8/siRNA/PLG was further coated with PLG (PVBLG-8/siRNA/PLG@PLG) to prevent clearance by anion-mediated resistance to protein adsorption in circulation.^344^ PLG was removed by protonation-mediated charge decrease in the GBM acidic microenvironment. Exposed PVBLG-8/siRNA then efficiently penetrated tumor cells and released anti-endothelial growth factor receptor (EGFR) siRNA. EGFR is a growth factor receptor overexpressed in tumor cells and can induce tumor cell differentiation and proliferation by binding to its ligand.^345^ The released EGFR inhibits the growth of GBM. Itaka et al. established a platform based on poly(aspartic acid) (PAsp). The authors synthesized PAsp(DET) by modifying diethylenetriamine (DET), a small molecule with primary and secondary amines, on the side chain of PAsp. The unique two-step protonation property of PAsp(DET) allows the side chain to undergo gauche to anti-conformational during the pH drop from 7.4 to 5.0 transition, which mediates degradation of the endosome/lysosome membrane and escape of nucleic acid drugs.^346,347^ PEG-PAsp(DET) delivered Neprilysin (NEP) mRNA to neurons after stereotaxic-guided ICV.^348^ NEP is a protease degrades Aβ and can degrade Aβ monomers and oligomers, effectively regulating AD’s early initiation and progression.^349–351^ This is the first study to validate the potential of exogenous mRNA for treating brain diseases. Subsequently, they used PEG-PAsp(DET) to deliver brain-derived neurotrophic factor (BDNF) mRNA to the brain for spinal cord injury (SCI)^352^ and ischemic neuronal death,^353^ respectively. BDNF is considered a potential candidate target for neuroprotection and regeneration and has a protective effect on neuronal survival after injury.^354–356^ Miyata et al. synthesized PAsp (DET/p) by modifying DET along with hydrophobic molecules (p). They controlled the octanol-water partition coefficient (log P) by modifying different p motifs to improve vector stability. PAsp(DET/p) delivered Cas9 mRNA to the brain, which is expected to enable gene editing of neural cells in the brain.^357^ Kataoka et al. then achieved the first RNA-based CRISPR/Cas9-induced genome editing in mouse brain parenchyma cells using PEG-PAsp(DET) delivery of Cas9 mRNA and sgRNA.^358^

Chitosan is a degradable cationic biopolymer widely used in medicine with excellent biocompatibility. The primary amine on the chitosan repeating unit provides positive charges and the proton sponge effect, which contributes to nucleic acid drug loading and endosome/lysosome escape. Woensel et al. cross-linked chitosan via sodium tripolyphosphate and electrostatically loaded anti-Galectine-1 (Gal-1) siRNA,^359^ an immunosuppressive protein^360^ that promoted tumor migration^361^ and angiogenesis.^362,363^ Chitosan facilitated the penetration of the vectors from the nasal cavity into the brain and downregulated the expression of Gal-1. Prakash et al. modified TAT on PEGylated chitosan and loaded it with siataxin-1, thus improving the cell permeability of the vectors and promising to treat spinocerebellar ataxia (SCA) over-expressing ataxin protein.^364^ Pego et al. obtained the neuronal targeting capability of the vectors by grafting a carboxylic fragment of the tetanus neurotoxin onto thiolated trimethyl chitosan (TMCSH-HC).^365^ The authors loaded BDNF pDNA by TMCSH-HC. The pDNA-loaded PNPs promoted damaged nerve recovery by preventing neurodegeneration and enhancing nerve regeneration, which provided a strategy for peripheral nerve injury treatment. Zhu et al. coupled cRGD-modified PEG with HA2 (GLFGAIAGFIENGWEGMIDGWYG)-modified chitosan via a hydrazone bond (cRGD-PEG-Hz-CS-HA2). The cRGD-PEG-Hz-CS-HA2 and octyl-Lys-9R were self-assembled into PNPs and adsorbed siVEGF.^366^ The HA2 was exposed in endosomal/lysosomal by pH-sensitive hydrazone bond breakage. HA2 was a pH-sensitive fusion peptide^367^ that facilitated the escape of siVEGF to the cytoplasm by fusing with the endosomal membrane through an irreversible conformational change,^368–370^ which treated GBM by downregulation of VEGF expression.

Other polysaccharide-like biomolecules have participated in the brain delivery of nucleic acid drugs. Yang et al. applied hyaluronic acid (HA) to improve nasal mucosal permeability and tumor targeting.^371^ They modified cholesterol on DP7 (VQWRIRVAVIRK) to provide a hydrophobic force (DP7-C). DP7-C electrostatically adsorbed siRNA and then encapsulated by HA (HA/DP7-C/siRNA). The HA adhesion ability improved the retention time in the nasal cavity, thus facilitating HA/DP7-C/siRNA penetration from the nasal cavity into the brain. HA further targeted tumor cells by specific binding to CD44 that overexpressed on the tumor surface.^372,373^ Anti-polo-like kinase 1 (PLK1) siRNA or anti-VEGF siRNA then treated GBM by downregulating PLK1 and VEGF, respectively. Kost et al. modified quaternary ammonium on pectic galactan.^374^ Quaternary ammonium provided positive charges for the electrostatic adsorption of pDNA. Galactose on pectic galactan could bind to Galectin-3 (Gal-3) on reactive gliosis specifically^375–378^ to mediate the targeted uptake of pDNA by reactive gliosis, which provided a strategy for the treatment of neuroinflammation. Sun et al. synthesized the pH-sensitive cationic dextran SpAcDex by modifying spermine at the acetalationoxidized dextran repeat unit. SpAcDex electrostatically adsorbed anti-miRNA-21 ASO and then modified B1 receptor ligand (B1L).^379^ B1L modification increased translocation and aggregation to tumor sites by binding to G-protein-coupled receptors expressed in tumor vessels and cells. miRNA-21 promotes tumor proliferation by inhibiting programmed cell death protein 4 (PDCD 4) and phosphatase tensin homolog (PTEN).^380,381^ The released ASO treated GBM by downregulating miRNA-21. Baigude et al. synthesized mannose-modified curdlan to load sip65.^382^ Mannose modification could promote the accumulation of sip65 in microglia through specific binding to mannose receptors.^383^ Stroke leads to NF-κB activation, which initiates pro-inflammatory cytokines and other potential neurotoxins that trigger neuroinflammation.^384–387^ sip65 could effectively inhibit the expression of NF-κB-regulated downstream genes by downregulating p65 inhibition, suppressing the neuroinflammatory response, attenuating edema formation, and reducing brain infarct volume.

The intelligent stability of the vector facilitates the long-term delivery of nucleic acid drugs in vivo and the efficient transfection of diseased cells. Shi et al. proposed a “triple-interactions” strategy and established a PEG-*b*-PGu-based nucleic acid drug delivery platform. Gu was guanidinium, which provided PNPs with charge interaction and hydrogen bonding interaction through the guanidinium-phosphate salt bridge. The authors co-polymerized phenylboronic ester on PEG-*b*-PGu (PEG-*b*-P(Gu/Hb)). The phenylboronic ester then provided hydrophobic interactions for the PNPs. These three interactions stabilized the PNPs. Modifying Ang on PNPs facilitated the vectors crossing the BBB and accumulating in tumors. High ROS in the tumors degraded the phenylboronic ester, thus eliminating hydrophobic interactions. Exposure to carboxyl groups further reduced charge interaction. The ROS-mediated destabilization of PNPs accelerated the release of siVEGFR2 and siPLK1 for GBM treatment.^388^ The authors replaced phenylboronic ester with tetrafluoropropyl methacrylate (PEG-*b*-P(GuF)) for siBACE1 delivery and AD treatment, demonstrating the universality of the “triple-interactions” strategy based on PEG-*b*-PGu in the treatment of brain disease.^144^

Dendrimers are a class of highly structurally controlled dendritic polymers constructed from branched repeat units.^389^ Dendrimers generally contain three parts: internal core, repeating units, and surface functionalization modifications.^390^ Poly(amidoamine) (PAMAM) dendrimers are the most common class of dendrimers employed to load nucleic acid drugs. Lee et al. used e-PAM-R, a degradable PAMAM dendrimer, loaded with siRNA against high mobility group box 1 (HMGB1) for neuroprotection in the postischemic brain.^391^ Excitotoxicity-induced cerebral ischemia promotes the release of HMGB1, which then induces microglia activation and neuron apoptosis.^392^ After intranasal administration, e-PAM-R delivered siHMGB1 to neurons and glial cells in brain regions such as the hypothalamus, amygdala, cerebral cortex, and striatum and suppressed post-ischemic rat brain infarct volume by down-regulating HMGB1 expression. Motoyama et al. conjugated cyclodextrin to PAMAM dendrimers to enhance the transfection of nucleic acid drugs. 6-*O*-*α*-(4-*O*-*α*-d-glucuronyl)-d-glucosyl-*β*-CyD (GUG-*β*-CyD)-conjugated PAMAM dendrimers (GUG-*β*-CDE(G3)) were able to promote nucleic acid drug escape from endosomes/lysosomes through the interaction of GUG-*β*-CyD molecules with endosomal/lysosomal membrane molecules. They used GUG-*β*-CDE (G3) for Cas9 RNP loading and delivery in vivo. After a single intraventricular administration, the GUG*-β*-CDE (G3) enhanced the gene editing activity of Cas9 RNP in the whole brain.^393^ Muñoz-Fernández et al. synthesized a fluorescein group-modified cationic carbosilane dendrimer (2G-(SNMe_3_I)_11_-FITC) for in vivo delivery of siRNA. After administration via retro-orbital venous plexus, 2G-(SNMe_3_I)_11_-FITC promoted siNef to cross the BBB and transfect HIV-1 infected cells, thereby reducing HIV-1 infectivity by downregulating Nef expression.^394^

The long RNA single-stranded structure of mRNA makes it more susceptible to being degraded by nucleases during delivery than other nucleic acid drugs. Therefore, improving the stability of mRNA before release is the major challenge in mRNA delivery. The mRNA condensation status in PNPs determines the stability of mRNA.^395–397^ Uchida et al. proposed a molecular hybridization strategy to improve mRNA stability by enhancing mRNA condensation in polymeric nanoparticles. They designed an RNA oligonucleotide linker with two complementary sequence arms to the mRNA. The mRNA nanoassemblies were prepared by hybridizing this linker with two mRNAs. The mRNA nanoassemblies prevented RNase attack by steric hindrance, contributing to a 100-fold increase in mRNA stability with the same translational activity.^398^ To further address the obstacle that anionic mRNA nanoassemblies were cleared in vivo and resistant to cellular uptake, Uchida et al. coated the nanoassemblies’ surface with cationic stealth coating (PEG-PLys), thus providing a strategy for stable mRNA delivery to the brain.^399^

**Fig. 4:**
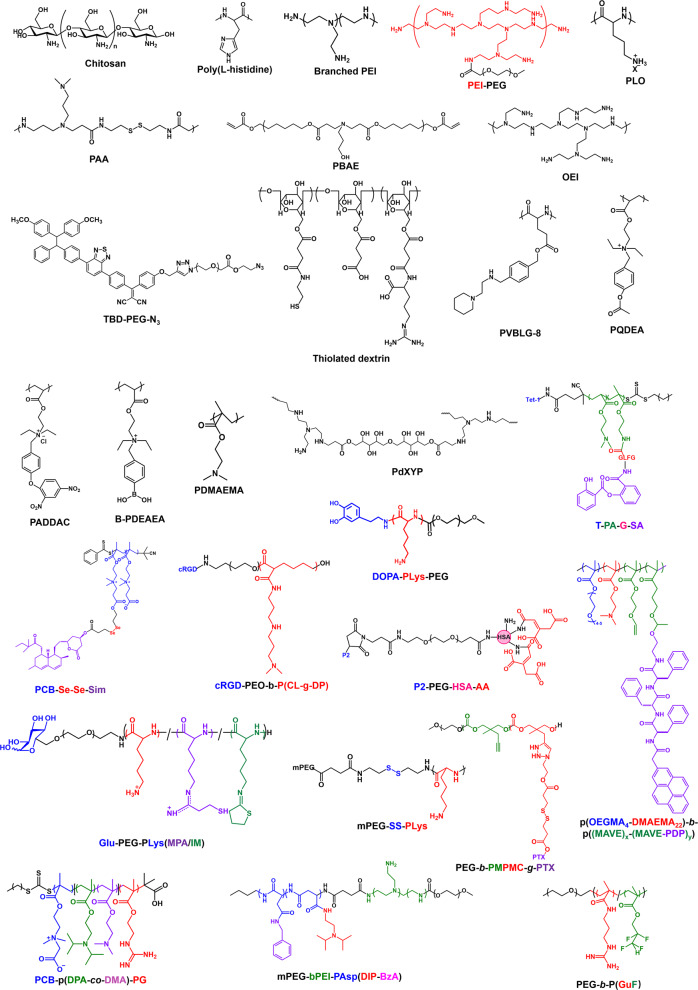
Chemical structures of polymers and polymer derivatives used for nucleic acid drug delivery. Chitosan; pHis, poly (L-histidine); branched PEI, polyethyleneimine; PEI-PEG, polyethyleneimine-*g*-poly(ethylene glycol); PLO, poly-L-ornithine; PAA, poly(amidoamine); PBAE, poly(β-amino esters); OEI, oligoethylenimine; TBD-PEG-N_3_, 1,1,2,2-tetraphenylethene-benzo[c][1,2,5] thiadiazole-2-(diphenyl methylene) malononitrile-polyethylene glycol-azide; Thiolated dextrin; PVBLG-8, poly(γ-(4-(((2-(piperidin-1-yl)ethyl)amino)methyl)benzyl-L-glutamate); PQDEA, poly(N-(2-(acryloyloxy)ethyl)-N-(*p*-acetyloxyphenyl)-N,N-diethylammonium chloride); PADDAC, poly(N-(2-(acryloyloxy)ethyl)-N-(*p*-(2,4-dinitrophenoxy)benzyl)-N,N-diethylammonium chloride); B-PDEAEA, poly((2-acryloyl)ethyl(*p*-boronic acid benzyl)diethylammonium bromide); PDMAEMA, poly(dimethylaminoethyl methacrylate); PdXYP, polydixylitol-polyethyleneimine; T-PA-G-SA, Tet-1-poly(2-(dimethylamino)ethyl acrylate)-*b*-poly(2-aminoethyl methacrylate-GFLG-salsalate); PCB-Se-Se-Sim, polycarboxybetaine-*co*-poly(polycarboxybetaine-Se-Se-simvastatin); cRGD-PEO-*b*-P(CL-*g*-DP), cRGD-poly(ethylene oxide)-*b*-poly(ɛ-caprolactone-*g*-N,N-dimethyldipropylenetriamine); P2-PEG-HSA-AA, poly(ethylene glycol)-human serum albumin-*cis*-aconitic anhydride; DOPA-PLys-PEG, dopamine-polylysine-*b*-poly(ethylene glycol); Glu-PEG-PLys(MPA/IM), glucosyl-poly(ethylene glycol)-*b*-poly(L-lysine modified with 3-mercaptopropyl amidine and 2-thiolaneimine); mPEG-SS-PLys, poly(ethylene glycol)-*b*-poly(L-lysine) bearing a disulfide linkage; PEG-*b*-PMPMC-*g*-PTX, poly(ethylene glycol)-*b*-poly(5-mthyl-5-propargyl-1,3-dioxan-2-one)-*g*-paclitaxel; p(OEGMA-DMAEMA)-*b*-p((MAVE)-(MAVE-PDP)), poly(oligo(ethylene glycol) monomethyl ether methacrylate-dimethylaminoethyl methacrylate)-*b*-poly((2-(vinyloxy)ethyl methacrylate)-((2 R)-N-((2 R,5 R)-5-benzyl-11,17-dimethyl-3,6,16-trioxo-1-phenyl-10,12-dioxa-4,7-diazaoctadec-17-en-2-yl)-3-phenyl-2-(2-(pyren-2-yl)acetamido)propanamide)); PCB-P(DPA-co-DMA)-PG, Poly(carboxybetaine)-*b*-poly(dimethylaminoethyl methacrylate-*co*-diisopropylethyl methacrylate)-*b*-poly(aminoethyl methacrylate); mPEG-bPEI-PAsp(DIP-BzA), monomethoxy-poly(ethylene glycol)-*b*-branched polyethyleneimine-*b*-poly(N-(N′,N′-diisopropylaminoethyl)-co-benzylamino)aspartamide; PEG-*b*-P(GuF), poly(ethylene glycol)-*b*-poly((N-(3-methacrylamidopropyl) guanidinium-co-2,2,3,3-tetrafluoropropyl methacrylate)

Overall, PNPs are powerful nucleic acid drug vector candidates. The performance and functionality of PNPs are primarily determined by the polymers. The polymers that we mentioned were shown in Fig. 4. The polymers’ design, synthesis, and functionalized modification are modular, meaning that polymers, monomers, and other small molecules with specific functions can be combined on a polymer as desired. The abundant monomer candidates and polymerization forms contribute to abundant polymer platforms. Modifying polymers’ end groups and repeating units enhances their properties and enriches their functions. The end groups mainly modify with ligand molecules to facilitate PNPs to overcome the BBB cellular uptake barriers. The repeat units are mainly modified with microenvironment-sensitive molecules to improve the performances of PNPs for drug loading and release and endosome/lysosome escape. Cationic polymers are still preferred for preparing PNPs due to the electrostatic adsorption of anionic nucleic acid drugs. However, their potential toxicity and short half-life in circulation have prompted researchers to focus on other interactions for stabilizing PNPs (covalent bonding, hydrogen bonding, and hydrophobic interactions) and degradation of PNPs in vivo. Biopolymers and biomolecule-like polymers are continuously developed as nucleic acid drug vectors. Anionic polymers or charge-tunable polymers have also participated in the preparation of PNPs. The research and development of PNPs at this stage should focus on their stability, long circulation, and biocompatibility to enable clinical applications. The researches on PNP-based nucleic acid drug vectors have been listed in Table 1.

**Table 1:** PNP-based nanoparticles for nucleic acid drug delivery

Polymer(s)	Disease	Nucleic acid drug	Therapeutic target	crossing BBB	cellular uptake	endosome/ lysosome escape	Drug release	Ref (s)
PEI	GBM	ASOs	miR-125b		AME	Proton sponge effect		^297^
PEI	PD	siRNA	SNCA	ICV injection	AME	Proton sponge effect		^301^
PLys-PEI	PD	pDNA	VEGF	stereotaxic-guided brain injection	AME	Proton sponge effect		^302^
PEG-PEI		pDNA		MRgFUS	AME	Proton sponge effect		^303^
PdXYP	GBM	siRNA	SHMT1	Penetration of nanochains		Proton sponge effect		^305,306^
PEI	AD	siRNA	BACE1	RVG29 and mannitol mediated	RVG29 and mannitol mediated	Proton sponge effect		^170^
PEI-SS- RVG29		miRNA	miR-124a	RVG29 mediated	RVG29 mediated	Proton sponge effect	GSH Disulfide bonds break	^312^
OEI		pDNA		iNGR mediated	iNGR mediated	Proton sponge effect	GSH Disulfide bonds break; cleaved by proteases	^316^
PHSA@PF/pDNA	AD	pDNA	TREM2	P2 mediated	P2 mediated	Proton sponge effect	pH Amide bond breakage	^321^
PEG-PDMAEMA	AD	siRNA	BACE1	CGN and QSH mediated	CGN mediated	Proton sponge effect		^331^
PDMAEMA	AD	siRNA	BACE1	Tet1 mediated	Tet1 mediated	Proton sponge effect		^332^
PBAE	GBM	siRNA, linear DNA, and circular DNA				Proton sponge effect	Esterase hydrolyze	^333^
PBAE		pDNA		Intracranial perfusion		Proton sponge effect		^334^
PBAE	GBM	pDNA	BMP4	Nasal injection		Proton sponge effect		^335,337,338^
PBAE	GBM	siRNA				Proton sponge effect	GSH Disulfide bonds break	^339,340^
PLO		siRNA	DR6			Proton sponge effect		^341^
GLU-PEG-PLys(MPA/IM)		ASO	MALAT1	GLU mediated	GLU mediated	Proton sponge effect	GSH Disulfide bonds break	^343^
PVBLG-8	GBM	siRNA	EGFR	CPP mediated	Cell penetrate α-helical peptide mediated	Proton sponge effect	pH Protonation-mediated charge decrease	^344^
PAsp(DET)	AD	mRNA	NEP	Stereotaxic guided ICV		Proton sponge effect	pH Gauche to anti-conformational	^346–348^
PEG-PAsp (DET)	Spinal cord injury (SCI), Ischemic neuronal death	mRNA	BDNF	In situ injection		Proton sponge effect	pH Gauche to anti-conformational	^352,353^
PAsp (DET/p)		Cas9 mRNA		In situ injection		Proton sponge effect		^357^
PEG-PAsp (DET)		Cas9 mRNA and sgRNA		In situ injection		Proton sponge effect		^358^
Chitosan	GBM	siRNA	Gal-1	Nasal injection		Proton sponge effect		^359^
PEGylated chitosan	SCA	siRNA	Ataxin-1	TAT mediated	TAT mediated	Proton sponge effect		^364^
TMCSH-HC	Peripheral nerve injury	pDNA	BDNF	Tetanus neurotoxin mediated	Tetanus neurotoxin mediated	Proton sponge effect		^365^
cRGD-PEG-Hz-CS-HA2	GBM	siRNA	VEGF	cRGD mediated	cRGD mediated	Proton sponge effect	pH Fusing with the endosomal membrane through an irreversible conformational change	^366^
HA/DP7-C/siRNA	GBM	siRNA	PLK1, VEGF	HA adhesion ability	HA mediated			^371–373^
Pectic galactan	Neuroinflammation	pDNA		In situ injection				^374^
SpAcDex	GBM	ASO	anti-miRNA-21	B1L mediated			pH	^379^
Curdlan	Stroke	siRNA	p65	Mannose mediated		Proton sponge effect		^382^
PEG-*b*-P(Gu/Hb)	GBM	siRNA	VEGFR2, PLK1	Ang mediated	Ang mediated	Proton sponge effect	ROS Phenylboronic ester degrade	^388^
PEG-*b*-P(GuF)	AD	siRNA	BACE1	Glut1 mediated	Glut1 mediated	Proton sponge effect		^144^
PEG-PLys		mRNA		In situ injection		Proton sponge effect		^399^

### Lipid-based nanoparticles

Lipid-based nanoparticles are nano-sized vesicles self-assembled by lipids. The membranes of lipid-based nanoparticles are structurally similar to biological membranes, such as cell membranes, EV membranes, and endosomal membranes, and thus possess an excellent affinity for biological membranes. Moreover, the formulation of lipid-based nanoparticles is precise and tunable, contributing to their almost batch-independent properties. Furthermore, lipid-based nanoparticles generally contain both hydrophilic and hydrophobic regions with tunable surface charges, allowing them to load hydrophilic and hydrophobic drugs with different charges and even load various drugs with different physicochemical properties in the same nanoparticle. These advantages make lipid-based nanoparticles the most approved class of vectors by the FDA. Patisiran, employing lipid-based nanoparticles as vectors, is the first new drug approved by the FDA based on RNA interference mechanisms for treating hereditary transthyretin-mediated (hATTR) amyloidosis.^400–402^ However, lipid-based nanoparticles are rapidly absorbed by RES and thus accumulate in the liver and spleen, which decreases the utilization of nucleic acid drugs and increases the burden on the liver and spleen. In addition, the escape ability of lipid-based nanoparticles from endosomes/lysosomes also severely affects the transfection efficiency of nucleic acid drugs. These are the key concerns currently focused on the development of lipid-based nanoparticles.

Cationic lipids contain a head group with permanent positive charges mainly provided by quaternary amines, such as DOTAP, DPDAP, and DOSPA, which are widely used for loading nucleic acid drugs.^2,403^ These cationic lipids are involved in nanoparticle self-assembly and loading nucleic acid drugs through electrostatic interactions. Delivering drugs to the brain is the primary challenge for vectors. Amiji et al. prepared DOTAP-based cationic nanoparticles and delivered mRNA^404^ and siRNA^405^ to the brain via intranasal administration. Teixeira et al. prepared cationic lipid-based nanoparticles to treat mucopolysaccharidosis type I (MPS I).^406^ α-L-iduronidase (IDUA) is a lysosomal enzyme responsible for the degradation of glycosaminoglycans (GAG) heparan and dermatan sulfate.^407^ DOPE, DOTAP, MCT, and DSPE-PEG2000 self-assembled to deliver IDUA pDNA to the brain via intranasal administration for treating MPS I by expressing IDUA. Braganhol et al. constructed cationic lipid-based nanoparticles loaded with siRNA for treating GBM after intranasal administration.^408^ CD73 promotes tumor growth, neovascularization, translocation, evasion of immune surveillance, and chemoresistance through AMP hydrolysis to produce adenosine.^409–411^ In addition, CD73 is involved in cell migration and invasion processes.^412–414^ So they selected siCD73 to inhibit tumor proliferation and migration. Temporary opening of BBB by FUS-MBs has also been used to promote the accumulation of lipid-based nanoparticles in the brain. Yeh et al. prepared vectors by DPPC, DPTAP, and DSPE-PEG2000 and delivered luciferase pDNA^415^ and prestin pDNA^416^ to the brain by FUS-MBs, respectively. Prestin, a naturally occurring ultrasound-responsive protein, enhances ultrasound sensitivity, thereby strengthening ultrasound’s ability to neuro-regulate deep tissue cells noninvasively in vivo, thus providing a new strategy for treating neurological disorders.^417,418^ Wang et al. prepared anionic nanoparticles by DSPE-PEG2000, DPPC. Interestingly, siRNA was attached to DSPE-PEG2000 on nanoparticles by biotin-avidin systems as linkers. After intravenous administration, the vectors accumulated in the brain with FUS-MBs’ assistance.^419^ Adding ligand molecules to the vector can facilitate drug delivery across the BBB through RMT. Chang et al. prepared self-assembled vectors from DOTAP and DOPE to load and deliver anti-superoxide dismutase 1 (SOD1) siRNA.^420^ They promoted vector crossing of the BBB by coating the vector surface with a single-chain fragment from the variable region of an anti-human single-chain antibody fragment to the transferrin receptor (TfRscFv). The siRNA accumulated in the brain could relieve neuroinflammation by downregulating SOD1 expression. Liu et al. prepared liposomes from DOTAP, DOPC, DSPE-PEG2000, and cholesterol.^421^ They modified Ang on the DSPE-PEG2000 of liposomes to promote anti-Golgi phosphoprotein3 (GOLPH3) siRNA across the BBB and accumulation in the GBM. Lima et al. modified CTX (MCMPCFTTDHQMARKCDDCCGGKGRGKCYGPQCLCR) on the surface of liposomes in the same approach, which contributing to the uptake of anti-miR-21 ASO and sisurvivin by glioma cells and downregulating miR-21 and survivin expression, respectively.^422^

Despite their apparent advantages in loading nucleic acid drugs, cationic lipids are susceptible to clearance in circulation, thus limiting their use in systemic administration. In addition, the toxicity of quaternary ammonium in cationic lipids must be seriously considered. In response, neutral or anionic lipids have also begun to be used for nucleic acid drug delivery. To address the challenge of anionic vectors resisting uptake by cells, Tagalakis et al. modified K16 CPP (KKKKKKKKKKKKKKKKKKKK) on anionic lipid-based nanoparticles, thereby improving the cell permeability of the vectors.^423^ The K16-modified anionic nanoparticles are biocompatible since K16 neutralizes the negative charge of the vector mainly through amino groups and is susceptible to degradation by proteases. Xu et al. prepared complexes of anionic siRNA with cationic protamine and then encapsulated the complexes in the core of anionic lipid-based nanoparticles.^424^ In addition, they modified APT_EDB_ (CSSPIQGSWTWENGK(C)WTWGIIRLEQ) on the surface of the vectors to target glioma cell-overexpressed extra-domain B (EDB) of fibronectin.^425–428^ Cyclophilin A (CypA), an immunophilin family protein, is a crucial determinant of protein folding,^429^ epithelial to mesenchymal transition, and cancer metastasis^430,431^ and is involved in maintaining glioma cell stemness through the Wnt/β-catenin signaling pathway.^432^ siRNA accumulated in tumors and treated GBM by downregulating CypA expression. Guan et al. adopted a similar strategy to enhance the accumulation of anionic lipid-based nanoparticles in the brain.^433^ siRNA was adsorbed on cationic Au nanoparticles (AuNPs) and then encapsulated in anionic liposomes modified with docosahexaenoic acid (DHA) and nerve growth factor (NGF). DHA and NGF facilitated vectors to cross the BBB and targeted uptake by neurons, respectively.^434^

Both cationic and anionic lipids exhibit poor endosome escape, which is thought to be the main factor for the ineffective transfection of nucleic acid drugs. Ionizable lipids have been employed to overcome this challenge. Ionizable lipids contain ionizable head groups that make them neutral at physiological pH and protonation-mediated positively charged under acidic environments, which gives ionizable lipids the following advantages: artificially low pH to enhance electrostatic interactions between the vectors and nucleic acid drugs during preparation; neutral vectors avoiding protein adsorption-mediated vector clearance in circulation and improving biocompatibility by avoiding interactions with blood cells and immune cells; escaping from acidic endosomes/lysosomes via the proton sponge effect.^2,59,403,435^ Heimberger et al. were the first to deliver miRNAs via LNPs for immunotherapy of tumors.^436^ They employed ATX, DSPC, and DMG-PEG2000 self-assembly to load and deliver miR-124, a key regulator of the signal transducer and activator of the transcription 3 (STAT3) pathway. The miR-124 stimulates anti-tumor immune responses by targeting the STAT3 signaling pathway, enabling the treatment of GBM.^437–440^ Peer et al. prepared ionizable lipid-based nanoparticles for siPLK1 delivery.^441^ PLK1, a serine/threonine-protein kinase overexpressed in tumors, is an early trigger of the G2/M transition in the cell cycle and plays an essential role in the malignant transformation of tumors.^442,443^ Modification of HA promoted the uptake of siPLK1 by glioma cells and GBM treatment after local administration. Almeida et al. constructed ionizable lipid-based nanoparticles to deliver siataxin-3 for the treatment of Machado-Joseph disease (MJD),^264^ which is caused by an excessive duplication of the CAG tract in the coding region of the ATXN3/MJD1 gene.^444^ Neuronal intranuclear inclusions caused by mutant ataxin-3 accumulation are pathological features of MJD.^445^ The ionizable nanoparticles modified by RVG29-9R efficiently cross the BBB and were internalized by neurons for the treatment of MJD through downregulating ataxin-3. Interestingly, Yu et al. modified nitroimidazole on the hydrophobic tails of ionizable lipids, thus conferring pH/hypoxia dual sensitivity to the lipids.^446^ In circulation, the nitroimidazole-modified ionizable nanoparticles are neutral, ensuring a long half-life and low toxicity. In the hypoxic GBM microenvironment, nitroimidazole converts to cationic aminoimidazole and imparts positive charges to the nanoparticles,^447,448^ thus accelerating the vectors’ cellular uptake. In acidic and hypoxic endosome/lysosome, tertiary amines and nitroimidazole are further protonated and transformed, thus facilitating the escape of nucleic acid drugs through the proton sponge effect and electrostatic interactions.

Lipids are the most critical determinants for lipid-based nanoparticle performance, such as the head group’s ionizable properties and the hydrophobic tail’s degradable properties. New lipids have been designed and synthesized. In particular, a series of lipids with similar structures and functions have been synthesized and screened for nucleic acid drug delivery. Peer et al. synthesized a series of ionizable lipids with unsaturated hydrophobic tails.^449^ These ionizable and unsaturated lipids were named X-ULFA in this Review. Their unsaturated tails with linoleic fatty acid were fixed, and their head groups with tertiary amines were variable. X-ULFA, DSPC, DMG-PEG, DSPE-PEG, and cholesterol self-assembled and loaded Cas9/sgRNA complexes against PLK1 (sgPLK1-cLNPs). A single dose of sgPLK1-cLNPs edited approximately 70% of the PLK1 gene and prolonged the median survival of GBM mice by about 50% after local administration.

Xu et al. synthesized a series of bioreducible ionizable lipid molecules (X-O14B) by the Michael addition reaction for enhanced endosome/lysosome escape and reduction-responsive drug release.^450^ X-O14B has fixed 14-carbon hydrophobic tails with bioreducible disulfide bonds and variable head groups with tertiary amines. X-O14B, DOPE, C_16_-PEG2000-ceramide, and cholesterol were self-assembled to load anionic Cas9/sgRNA complexes. These CRISPR/Cas9 system-loaded nanoparticles exhibited excellent gene editing efficacy after local administration in the dorsomedial hypothalamic nucleus, mediodorsal thalamic nucleus, and bed nucleus of the stria terminalis, respectively. Subsequently, they synthesized other bioreducible hydrophobic tails with disulfide bonds and cholesteryl (OCholB).^451^ A series of cholesteryl-based biodegradable ionizable lipids (X-OCholB) were synthesized to deliver mRNA by introducing various ionizable head groups with tertiary amines onto OCholB via the Michael addition reaction. These mRNA-loaded nanoparticles were able to transfect part of the neurons in stratum oriens of Field CA1, CA2, and CA3 in hippocampal formation AmmonQ’s horn after local administration in the lateral ventricle.

Akita et al. also synthesized a series of bioreducible ionizable lipid-like molecules.^452^ These SS-cleavable proton-activated lipid-like materials were named ssPalm. Each ssPalm contained three structural units: a pair of hydrophobic tails for forming bilayers, a pair of tertiary amine groups for protonation, and a disulfide bond for degradation in the cytoplasm. By modifying different molecules at the ends of this pair of hydrophobic tails, they synthesized a series of ssPalm. DOPE, ssPalm, DMG-PEG2000, and cholesterol self-assembled into vectors and loaded mRNA. These mRNA-loaded nanoparticles were efficiently transfected into neurons and astrocytes after local administration. Subsequently, they synthesized a series of oleic acid-scaffold self-degradable lipid-like materials (ssPalmO-X) by introducing vinyl and ester groups based on ssPalm.^453^ ssPalmO-X could self-degrade via hydrolysis accelerated by intra-particle enrichment of the reactant (HyPER). Firstly, GSH cleaved the disulfide bond, thus decomposing ssPalmO-X into two separated thiol-containing tails. Subsequently, the thiol concentration gradually increased with the continuous reduction of the disulfide bond. Subsequently, the thiol concentration gradually increased with the constant reduction of the disulfide bond. Finally, the enriched thiols attack the unstable ester bonds, accelerating the degradation of the hydrophobic tails. DOPC, ssPalmO-Phe, DMG-PEG2000, and cholesterol self-assemble and load mRNA.^454^ FUS-MBs facilitated the accumulation of mRNA-loaded nanoparticles in the brain after systemic administration. ssPalmO-Phe assisted mRNA escaping from the endosome/lysosome and rapid release in the cytoplasm, thus contributing to the mRNA-induced protein expression in glial cells.

**Fig. 5:**
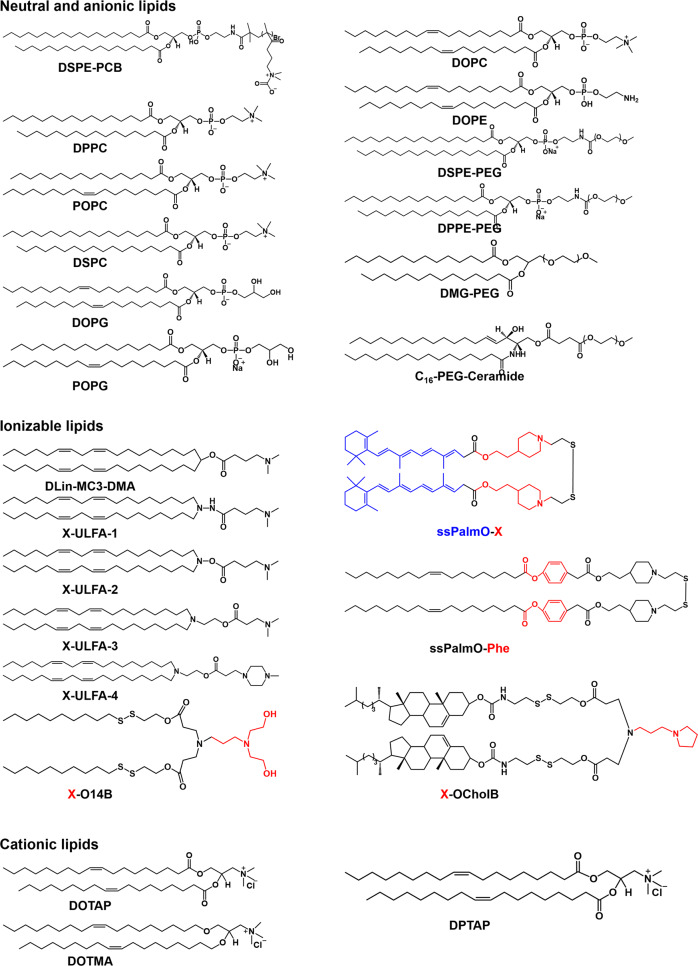
Chemical structures of lipids and lipid derivatives used for nucleic acid drug delivery. DSPE-PCB, 1,2-distearoyl-sn-glycero-3-phosphoethanolamine-polycarboxybetaine; DSPE-PEG, 1,2-distearoyl-sn-glycero-3-phosphoethanolamine-poly(ethylene glycol); DPPC, 1,2-dipalmitoyl-sn-glycero-3-phosphocholine; DOPC, 1,2-dioleoyl-sn-glycero-3-phosphocholine; POPC, 1-palmitoyl-2-oleoyl-glycero-3-phosphocholine; DOPE, 1,2-dioleoyl-sn-glycero-3-phosphoethanolamine; DSPC, 1,2-distearoyl-sn-glycero-3-phosphocholine; DPPE-PEG, 1,2-dipalmitoyl-sn-glycero-3-phosphoethanolamine-poly(ethylene glycol); DOPG, 1,2-dioleoyl-sn-glycero-3-phospho-(1’-rac-glycerol); DMG-PEG, 1,2-dimyristoyl-rac-glycero-3-methoxy-poly(ethylene glycol); POPG, 1-palmitoyl-2-oleoyl-sn-glycero-3-phospho-(1’-rac-glycerol) (sodium salt); DLin-MC3-DMA, (6*Z*,9*Z*,28*Z*,31*Z*)-heptatriaconta-6,9,28,31-tetraen-19-yl 4-(dimethylamino)butanoate; C_16_-PEG2000-Ceramide, N-palmitoyl-sphingosine-1-(succinyl(methoxy(polyethylene glycol)2000)); X-ULFA-1, 4-(dimethylamino)-N’,N’-di((9*Z*,12*Z*)-octadeca-9,12-dien-1-yl)butanehydrazide; X-ULFA-2, 4-((di((9*Z*,12*Z*)-octadeca-9,12-dien-1-yl)amino)oxy)-N,N-dimethyl-4-oxobutan-1-amine; X-ULFA-3, 2-(di((9*Z*,12*Z*)-octadeca-9,12-dien-1-yl)amino)ethyl 3-(dimethylamino)propanoate; X-ULFA-4, 2-(di((9*Z*,12*Z*)-octadeca-9,12-dien-1-yl)amino)ethyl 3-(4-methylpiperazin-1-yl)propanoate; X-O14B, bis(2-(decyldisulfaneyl)ethyl) 3,3’-((3-(bis(2-hydroxyethyl)amino)propyl)azanediyl)dipropionate; ssPalmO-X, ((disulfanediylbis(propane-3,1-diyl))bis(piperidine-1,4-diyl))bis(ethane-2,1-diyl) (3*E*,3’*E*,5*E*,5’*E*,7*E*,7’*E*,9*E*,9’*E*)-bis(4,8-dimethyl-10-(2,6,6-trimethylcyclohex-1-en-1-yl)deca-3,5,7,9-tetraenoate); ssPalmO-Phe, ((((((disulfanediylbis(ethane-2,1-diyl))bis(piperidine-1,4-diyl))bis(ethane-2,1-diyl))bis(oxy))bis(2-oxoethane-2,1-diyl))bis(4,1-phenylene))bis(methylene) dioleate; X-OCholB, bis(2-((2-((((17-isopentyl-10,13-dimethyl-2,3,4,7,8,9,10,11,12,13,14,15,16,17-tetradecahydro-1*H*-cyclopenta[*a*]phenanthren-3-yl)oxy)carbonyl)amino)ethyl)disulfaneyl)ethyl) 3,3’-((3-(pyrrolidin-1-yl)propyl)azanediyl)dipropionate; DOTAP, 1,2-dioleoyl-3-trimethylammonium-propane (chloride salt); DPTAP, 1,2-dipalmitoyl-3-trimethylammonium-propane (chloride salt); DOTMA, 1,2-di-O-octadecenyl-3-trimethylammonium propane (chloride salt)

Overall, the physicochemical properties of lipid-based nanoparticles, such as size, shape, structure, surface charge, and lipid structure, affect the half-life and biodistribution of the vectors in circulation. Ionizable lipids are preferred over cationic, neutral, and anionic lipids. The degradable properties of lipids influence endosome/lysosome escape and drug release. In addition, introducing stealth polymers or ligand molecules in lipid-based nanoparticles is also a strategy to prolong half-life, reduce toxicity, and enhance accumulation in lesions. Notably, the synthesis and screening of novel lipids by designing lipid head groups and hydrophobic tails to adapt different types of nucleic acid drugs and diseases are more promising research strategies currently. The lipids that mentioned in this Review were shown in Fig. 5. The researches of lipid-based nanoparticles as nucleic acid drug vectors have been summarized in Table 2.

**Table 2:** Lipid-based nanoparticles for nucleic acid drug delivery

Composition(s)	Disease	Nucleic acid drug	Therapeutic target	crossing BBB	cellular uptake	endosome/lysosome escape	Drug release	Ref (s)
DOTAP, DPPC, Cholesterol		mRNA		Nasal injection				^404^
DOTAP	Neuroinflammation	siRNA	TNFα	Nasal injection				^405^
DOPE, DOTAP, MCT (NE), and DSPE-PEG2000	MPS I	pDNA	IDUA	Nasal injection				^406^
DOTAP, MCT	GBM	siRNA	CD37					^408^
DPPC, DPTAP, and DSPE-PEG2000	Neurological disorders	pDNA	Prestin	FUS-MBs				^415,416^
DSPE-PEG2000, DPPC	GBM	siRNA	IDH1	FUS-MBs				^419^
DOTAP and DOPE	Neuroinflammation	siRNA	SOD1	TfRscFv mediated	TfRscFv mediated			^420^
DOTAP, DOPC, DSPE-PEG2000, and cholesterol	GBM	siRNA	GOLPH3	Ang mediated	Ang mediated			^421^
DOTAP, DSPC, C_16_-PEG2000-Ceramide, cholesterol	GBM	ASO siRNA	anti-miR-21 Survivin	CTX mediated	CTX mediated	Proton sponge effect		^422^
DOPG, DOPE, DPPE-PEG2000/DOPE-PEG2000	AD	siRNA	BACE1		K16 mediated			^423^
POPC, POPG, DSPE-PEG, cholesterol	GBM	siRNA	CypA	APT-EDB mediated				^424–428^
Lecithin and cholesterol	PD	pDNA	α-syn	DHA mediated	DHA mediated			^433^
ATX, DSPC, and DMG-PEG2000	GBM	miRNA	miR-124					^436^
Dlin-MC3-DMA, DSPC, DMG-PEG- cholesterol	GBM	siRNA	PLK1	In situ injection				^441^
DODAP, DSPC, C_16_-PEG2000- Ceramide, cholesterol	MJD	siRNA	Ataxin-3	RVG29 mediated				^264^
DSPE-PEG-2000, MDH, cholesterol	GBM	siRNA	PLK1			Proton sponge effect	pH/hypoxia	^446^
X-ULFA, DSPC, DMG-PEG, DSPE-PEG	GBM	Cas9 mRNA, sgRNA	PLK1	In situ injection				^449^
X-O14B, DOPE, C_16_-PEG2000-ceramide, cholesterol		Cas9/sgRNA complexes		In situ injection			GSH SS- cleaved	^450^
X-OCholB		mRNA		In situ injection				^451^
DMG-PEG2000, DOPE, ssPalm, cholesterol		mRNA					GSH SS- cleaved	^452^
ssPalmO-X		mRNA		FUS-MBs and in situ injection			GSH SS- cleaved	^453^

### EVs

EVs refer to a heterogeneous population of vesicular bodies secreted by cells, including ectosomes (50 nm~1 μm in diameter) formed by outward budding from the surface of the plasma membrane and exosomes (40 nm to 160 nm in diameter) derived from endosomes.^455^ This Review will emphasize the application of exosomes as nucleic acid drug vectors in brain disease treatment. Exosomes are natural vehicles for cell-to-cell communication and contain bilayers and cavities similar to liposomes.^456,457^ Exosomes exhibit lower clearance and lower toxicity than lipid-based nanoparticles in circulation.^458–461^ In addition, exosomes have natural ligands distributed on their surface that tend to be taken up by specific cells.^456,462,463^ Moreover, exosomes can also be artificially modified with exogenous ligands on their surface to enable them with the desired tissue targeting.^464,465^ Furthermore, exosomes naturally carry various bioactive molecules such as RNA, DNA, proteins, and lipids.^466–468^ These properties potentially allow exosomes to deliver nucleic acid drugs for brain disease treatment.

The heterogeneity of exosomes, such as size, charge, content, and surface ligands, allows different exosome subtypes to perform distinct drug delivery behaviors. Cellular uptake of exosomes is determined by their surface-specific components, such as saccharides, lipids, and proteins, as well as surface charge and size, which not only determines the ability of exosomes to cross tissue and cellular barriers but also causes variation in the delivery efficiency of exosomes among different cells.^462,463,469^ In addition, exosomes derived from different cells and tissues perform different biological functions.^466^ Exosomes from plasma can promote cardiac cell repair.^470^ MSC-derived exosomes are non-inflammatory and exhibit neuroprotective effects.^471–474^ Dendritic cell-derived exosomes can present antigens and activate T-lymphocytes.^475,476^ Exosomes derived from red blood cells of type O donors are more prone to drug loading.^477^ Astrocyte-derived exosomes promote neurodegeneration.^478^ Tumor cell-derived exosomes may stimulate tumor immune responses by presenting tumor-associated antigens but probably facilitate cancer progression and metastasis.^479–481^ Therefore, selecting appropriate cell-derived exosomes adapted to nucleic acid drugs, diseases, and clinical applications is the primary consideration.

The isolation and extraction of exosomes remain challenging currently. High yield and homogeneity of exosomes production are necessary for their clinical translation. One strategy to increase production is to promote the cellular secretion of exosomes.^466^ The six-transmembrane epithelial antigen of the prostate family member 3 (STEAP3) is involved in the biogenesis of exosomes, thus promoting the secretion of EVs.^482^ Heparanase can also increase the production of EVs by regulating the secretion, composition, and engineering of exosomes.^483^ Hypoxia,^484^ oxidative stress,^485^ ionizing radiation,^486^ and acidic environments^487^ also promote EV secretion. The commonly used methods for exosome extraction are ultracentrifugation, magnetic-activated cell sorting, and chemical precipitation.^488^ Ultracentrifugation, including differential centrifugation, is still the preferred method to obtain exosomes.^489^ Monastyrskaya et al. developed an optimized differential ultracentrifugation and size exclusion chromatography method to improve the yield and homogeneity of urine-derived exosomes.^490^ Simpson et al. compared LIM1863 cell-derived exosomes extracted by centrifugation, density-based separation, and EpCAM immunoaffinity capture. The enrichment of exosome markers and associated proteins was over twice as high as that of the centrifugation and density-based separation extracts. Therefore, immunoaffinity capture was the best exosome extraction method.^491^ Lee et al. constructed an acoustic nanofilter system for separating microvesicles of specific sizes in a continuous and contact-free manner. This system uses ultrasound standing waves to apply different acoustic forces to microvesicles with different sizes and densities and achieves more than 90% separation yields with an in situ controlled particle size cutoff.^492^ Stolovitzky et al. established nanoscale deterministic lateral displacement arrays by manufacturable silicon processes. The arrays separated exosomes between 20 and 110 nm in diameter with sharp resolution, which paved the way for the sorting and quantifying of exosomes.^493^ Lyden et al. identified two exosome subpopulations with different sizes by asymmetric flow field-flow fractionation. This method can isolate EVs and provide a strategy to address the challenge of exosome heterogeneity.^494^

Modifying functional molecules, such as ligands, on exosome membranes can endow exosomes with desired functions. The exosome membrane mainly consists of a bilayer formed by phospholipid molecules in a tail-to-tail pattern through their hydrophobic tails. The exosome surface is typically modified by coupling a hydrophobic motif to the functional molecule and then inserting this hydrophobic motif into the hydrophobic tails of the exosome phospholipid bilayer.^495,496^ In addition, functional molecules can be directly modified on the exosome surface by condensation reactions or click chemistry.^465,497–499^ Remarkably, the reaction conditions, such as temperature, pH, and osmolarity, must be gentle, and the reaction substrates must be minimal enough so that the modified exosomes remain active. Multivalent electrostatic^500,501^ and ligand-receptor^502,503^ interactions have also been applied for surface modification of exosomes. Interestingly, Kim et al. creatively transfected genes encoding functional biomolecules into origin cells and then extracted exosomes, thereby endogenously generating functional molecules on exosomal membranes.^504^ Loading nucleic acid drugs into the exosome’s lumen involves crossing the exosome’s phospholipid bilayer. There are four main approaches to drug loading: electroporation,^505^ ultrasound,^506^ gene transfection,^507^ and simple incubation.^508^ The main potential disadvantages of the electroporation method for encapsulating nucleic acid drugs are size-dependent loading efficiency,^509^ precipitation and degradation of nucleic acid drugs,^510^ and exosome destabilization.^511^ The ultrasound is removed when the drug is sufficiently mixed with the exosomal components. The exosomal components and drugs are reassembled, thus encapsulating the drug in the exosome. This approach may affect the size, morphology, and homogeneity of the exosomes and also exposes the risk of losing some key components of the exosomes. Although transfection and simple incubation can improve the stability and integrity of exosomes and drugs, they sacrifice the amount of drug loading.

Exosomes contain a diverse range of endogenous nucleic acid molecules, such as DNA, mRNA, ribosomal RNA, miRNA, transfer RNA, mitochondrial RNAs, Dicer, and Argonaute.^459,466,467,512–517^ miRNAs are the most extensively studied endogenous nucleic acid molecules in exosomes and can treat a wide range of diseases with a wide range of downstream effects. Adipose-derived stem cell (ADSC)-derived exosomes contain miR-188-3p and inhibit neuronal autophagy and pyroptosis by downregulating NAcht Leucine-rich repeat protein 3 (NLRP3) and cell division protein kinase 5 (CDK5), contributing to the PD treatment.^518^ M2 microglia-derived exosomes containing miR-124 can mediate neuroprotective effects and contribute to treating ischemic cerebral injury.^519^ Exosomes loaded with exogenous miR-124 by electroporation or transfection also play a therapeutic role in CNS diseases such as ischemic cerebral injury,^520^ traumatic cerebral injury,^521^ and HD.^522^ Typically, Hu et al. co-transfected dendritic cells with Dicer siRNA and RVG-Lamp2b plasmids. Dicer siRNA depleted endogenous miRNAs, thereby avoiding interference from heterogeneous miRNAs. Lamp2b was an endosomal membrane protein and promoted the integration of RVG-Lamp2b into exosomal membranes after transfection. miR-124 was encapsulated in dendritic cell-derived exosomes via the ExoFect Exosome Transfection Reagent to treat cocaine-mediated neuroinflammation.^523^ MSC-derived exosomes loaded with endogenous miR-133b can attenuate the development of GBM through disruption of the Wnt/β-catenin signaling pathway by inhibiting the Enhancer of Zeste 2 (EZH2).^524^ miR-133b-loaded exosomes by transfection can also inhibit neuronal apoptosis by regulating extracellular signal regulating kinase (ERK1/2)/ cAMP response element-binding protein (CREB) and RhoA, thus exerting a neuroprotective effect, which contributes to the treatment of intracerebral hemorrhage^525^ and ischemic stroke.^526^ miR-210 is the main hypoxia-induced miRNA that promotes angiogenesis mediated through the HIF-1α/VEGF/Notch 1 signaling pathway, thereby upregulating focal angiogenesis and neuroprotective effects after CI.^527–531^ Peng et al. found that middle cerebral artery occlusion (MCAO) rats had elevated levels of miR-210 in serum-derived exosomes after electro-acupuncture treatment, which contributed to ischemic stroke treatment.^532^ Gao et al. then loaded cholesterol-modified miR-210 into MSC-derived exosomes via hydrophobic interaction. In addition, the authors modified c(RGDyK) directly on the exosome surface by the click chemistry to treat CI.^533^

Excessively high or low levels of endogenous miRNAs in the body may induce disease. Therefore, miRNAs or miRNA inhibitors contribute to treating various diseases. The miR-302~367 cluster enables glioma stem-like cells (GSCs) to enter an irreversible differentiation state and blocks the ability of GSCs to initiate and develop tumors in vivo.^534^ Virolle et al. found that exosomes extracted after transfecting patients’ GSCs with miR-302~367 cluster had GBM therapeutic effects.^535^ MSC-derived exosomes can reduce the viability and clonogenicity of GCSs after loading miR-124a by transfection, contributing to GBM treatment.^536^ You et al. found that exosomes from TMZ-resistant GBM samples lacked miR-151a. After miR-151a was restored by transfection, these exosomes were able to significantly reduce the level of XRCC4, inducing a delay in the clearance of DNA double-strand breaks and prompting GBM to be TMZ-sensitive.^537^ Brain endothelial cell-derived exosomes loaded with miR-126 can increase vascular density and arterial diameter, enhance axone and myelin density in the ischemic boundary zone, and induce M2 macrophage polarization, contributing to improved neurological function and cognition in type 2 diabetes mellitus-stroke mice.^538^ MSC-derived exosomes loaded with miR-193b-3p acetylated NF-κB p65 by inhibiting HDAC3 expression and activity, thereby reducing the inflammatory response. These effects attenuated neurobehavioral deficits and neuroinflammation after subarachnoid hemorrhage.^539^ MSC-derived exosomes loaded with miR-17~92 cluster play an essential role in mediating the function of neural progenitor cells by locally regulating PTEN protein levels, promoting axonal outgrowth of embryonic cortical neurons, and increasing cell proliferation and inhibiting cell death.^540^ As mentioned previously, miR-21 is one of the most highly expressed miRNAs in tumors and exerts oncogenic effects by inhibiting PDCD4 and PTEN. Lee et al. loaded anti-miR-21 ASOs in HEK293 cell-derived exosomes by electroporation to treat GBM.^541^ miR-181a is overexpressed in ischemic brain tissue, downregulates the Bcl-2 anti-apoptotic protein, and induces apoptosis.^542–544^ Lee et al. further encapsulated anti-miR-181 ASO with cholesterol modification into HEK293 cell-derived exosomes by hydrophobic interaction to attenuate ischemic neuronal injury by increasing Bcl-2 expression.^545^

In 2011, Wood et al. first applied engineered exosomes as siRNA vectors for brain disease treatment. They transfected RVG29-Lamp2b into dendritic cells. The affinity interaction of Lamp2b with endosomes allowed the expressed RVG29-Lamp2b to migrate toward endosomes and secrete exosomes enriched with the ligand RVG29. siBACE1 was inserted into the exosomes by electroporation for AD treatment.^546^ Their research provided a paradigm for developing engineered exosomes as nucleic acid drug vectors. Subsequently, they discovered that glyceraldehyde-3-phosphate dehydrogenase (GAPDH) binds to EVs via a phosphatidylserine binding motif (G58). They fused the GAPDH-derived G58 peptide to dsRNA-binding motifs, thereby substantially increasing the loading efficiency of siRNAs in EVs.^547^ Hydrophobic modifications on siRNA can also promote the exosome loading of siRNA through hydrophobic effects.^548^ Amplifying the CAG repeat sequence of the huntingtin gene (HTT) leads to HD.^549^ Silencing wild-type and mutant HTT alleles in the striatum and cortex is a promising strategy for treating HD.^550–553^ Delivery of siHTT via exosomes has facilitated the treatment of HD.^547,548,554^ PTEN is expressed in neurons and regenerating axons and inhibits axonal growth by downregulating mTOR activity.^555–558^ MSC-derived exosomes loaded with siPTEN could be enriched in the brain by intranasal delivery. siPTEN was delivered to the injury area in complete spinal cord injury rats benefiting from the tendency of MSC-derived exosomes to the injury site, which promoted axonal regeneration.^559^ Exosomes loaded with siRNA have been developed for the immunotherapy of GBM. Radiotherapy promotes the expression of programmed cell death-ligand 1 (PD-L1), which impairs anti-tumor immunity.^560^ ReNcell VM (ReNcell)-derived exosome surface was modified with the ligand molecule c(RGDyK) by click chemistry. Cholesterol-modified siPD-L1 was then encapsulated in exosomes by hydrophobic interaction. Under the guidance of c(RGDyK), exosomes delivered siPD-L1 to GBM, downregulated radiation-induced PD-L1 expression, and activated anti-tumor immunity.^561^ RVG29-modified exosomes inhibited Zika virus (ZIKV) infection in the fetal brain after encapsulating ZIKV-specific siRNA.^562^ In addition, RVG29-modified exosomes encapsulated with anti-SNCA siRNA^563^ or shRNA^564^ have also exhibited excellent PD therapeutic effects. Liang et al. combined exosomes with PNPs.^565^ They encapsulated siRNA in DSPE-PEG-PEI to form PNPs. DSPE-PEG-cRGD was inserted into the exosome membrane by hydrophobic interaction. The siRNA-loaded PNPs were then encapsulated into the exosomes by the liposome extruder. The protein phosphatase Mg^2+^/Mn^2+^ dependent 1D (PPM1D) gene has been selected as a therapeutic target. It promotes dephosphorylation of p53 and γ-H2AX, leading to the inactivation of the DNA damage response.^566^ The modified exosomes delivered siPPM1D to the tumor region for diffuse intrinsic pontine glioma treatment.

Exosomes can also deliver mRNA for the treatment of brain diseases. NGF promotes nerve growth and has overall protective effects on different neurons, gliocytes, and vascular endothelial cells.^567^ HEK293 cells-derived exosomes modify RVG and are loaded with NGF mRNA by transfection. The modified exosomes contributed to the treatment of stroke after intravenous administration.^568^ Lee et al. developed a cellular nanoporation (CNP) biochip to stimulate cells to produce and secrete exosomes rich in nucleic acid drugs, such as mRNA.^507^ Briefly, a monolayer of cells was seeded on the surface of a chip containing nanochannels (500 nm in diameter), and pDNA was added to the medium on the side of the unseeded cells. The transient electrical pulse (cathode on the side where the pDNA was located and anode on the side where the monolayer cells were located) accelerated the pDNA across the nanochannel and uptake by the monolayer cells. Exosomes containing transcribed mRNA secreted on the anodic side were captured by transient electrical pulse-driven migration. This approach boosted exosome yield by 50-fold and exosomal mRNA transcripts yield by 1000-fold. PTEN mRNA-enriched exosomes obtained by the CNP chip for PTEN-deficient glioma mouse models restored tumor suppression, enhanced tumor growth inhibition, and increased survival.

The study of Wood et al. in 2011 pioneered a decade of using exosomes as nucleic acid drug vectors. Researchers began proposing new exosome applications in the third decade of the 21st century. In 2021, Chen et al. proposed a strategy to stimulate host tissue to secrete therapeutic exosomes in vivo.^554,569^ These exosomes with nucleic acid drugs and ligands directly migrate to the lesion for disease treatment. That is, there is no need to isolate, extract, purify, and modify exosomes and load drugs onto exosomes in vitro. As a paradigm, they designed a plasmid-based genetic circuit to transform the host liver into a tissue chassis that secretes exosomes expressing RVG29 and anti-mutant HTT (mHTT) siRNA.^554^ These exosomes could cross the BBB and target neurons with the assistance of RVG29 and downregulate mHTT expression by simHTT to treat HD. This genetic circuit has the potential to express a wide range of nucleic acid drugs and ligand molecules, thus possessing broad potential for the treatment of multiple diseases. This approach is expected to overcome the concerns about yield, heterogeneity, and immunogenicity faced in the clinical translation of exosomes and can also dramatically simplify the application patterns and costs of exosomes.

Overall, EVs, represented by exosomes, have been developed as nucleic acid drug vectors with unique advantages. Multiple EV extraction, modification, and drug-loading methods have been developed. The challenges for the clinical translation of EVs are mainly yield and heterogeneity. Increasing the yield and reducing the cost of EVs are the primary issues currently addressed in application-oriented EVs’ development. It is also crucial to homogenize EVs as much as possible to overcome the influence of size, surface charges, and endogenous cargoes. In addition, the application patterns of exosomes should be rethought. The researches on EVs as nucleic acid drug vectors have been summarized in Table 3.

**Table 3:** EV-based nanoparticles for nucleic acid drug delivery

Origin	Disease	Nucleic acid drug	Therapeutic target	Drug loading method	Ligand(s)	Modification method	Ref (s)
ADSC	PD	miRNA	miR-188-3p	Endogenous			^518^
M2 microglia	Ischemic cerebral injury, traumatic cerebral injury, and HD	miRNA	miR-124	Electroporation or transfection			^519–522^
Dendritic cells	Cocaine-mediated neuroinflammation	miRNA	miR-124	Transfection	RVG-Lamp2b	Transfection	^523^
MSC	GBM	miRNA	miR-133b	Endogenous			^524^
MSC	Intracerebral hemorrhage, ischemic stroke	miRNA	miR-133b	Transfection			^525,526^
Serum of MCAO rats after electroacupuncture treatment	Ischemic stroke	miRNA	miR-210	Endogenous			^532^
MSC	CI	miRNA	miR-210	Hydrophobic interaction	c(RGDyK)	Click chemistry	^533^
GSCs of GBM patients	GBM	miRNA	miR-302~367	Endogenous			^535^
TMZ-resistant GBM samples	GBM	miRNA	miR-151a	Transfection			^537^
MSC	GBM	miRNA	miR-124a	Transfection			^536^
Brain endothelial cells	Type 2 diabetes mellitus-stroke	miRNA	miR-126	Endogenous			^538^
MSC	Neuroinflammation	miRNA	miR-193b-3p	Endogenous			^539^
MSC	Stroke	miRNA	miR-17~92	Transfection			^540^
HEK293 cell	GBM	ASO	anti-miR-21	Electroporation	T7	Transfection	^541^
HEK293 cell	Ischemic neuronal injury	ASO	anti-miR-181	Hydrophobic interaction			^545^
Dendritic cells	AD	siRNA	BACE1	Electroporation	RVG29-Lamp2b	Transfection	^546^
MSC	Spinal cord injury	siRNA	PTEN	Incubation			^559^
ReN cell	GBM	siRNA	PD-L1	Hydrophobic interaction	c(RGDyK)	Click chemistry	^561^
HEK293T cell	ZIKV infection in the fetal brain	siRNA	ZIKV	Electroporation	RVG29	Transfection	^562^
Dendritic cell	PD	siRNA shRNA	SNCA	Electroporation	RVG29	Transfection	^563,564^
RAW264.7 cell	Diffuse intrinsic pontine gliomas	siRNA	PPM1D	Extruder	cRGD	Hydrophobic interaction	^565^
HEK293 cell	Stroke	mRNA	NGF	Transfection	RVG29		^568^
HEK-293T cell, MSC, dendritic cell	GBM	mRNA	PTEN	Transfection	CDX	Transfection	^507^
Hepatocyte	HD	siRNA	HTT	Transfection	RVG29	Transfection	^554^

### Inorganic nanoparticles or organic-inorganic hybrid nanoparticles

Inorganic nanoparticle-based vectors are mainly crystal structures and can be precisely designed and prepared into various desired sizes, shapes, topographies, and surface potentials. In addition, most inorganic nanoparticles are simple to prepare, have a slight variation between batches, have excellent dispersion and homogeneity, and can be stored stably. Moreover, some inorganic nanoparticles possess optical, magnetic, radioactive, and plasmonic properties that can be used as adjunctive therapies or diagnostics for diseases. These unique advantages promote the application of inorganic nanoparticles in vector development.

AuNPs possess highly tunable physicochemical properties, such as size, shape, surface charge distribution, topography, hydrophilic/hydrophobic properties, and well-established and simple preparation methods.^570–572^ In addition, localized surface plasmon resonance (LSPR) enables AuNPs to be applied in photothermal therapy,^573^ photodynamic therapy,^574^ photoacoustic imaging,^575^ computerized tomography (CT),^576^ and fluorescence quenching/activation.^577–579^ Furthermore, AuNPs have a large specific surface area and can easily modify organic molecules. Therefore, AuNPs are widely applied in biomedical fields.^580–582^ Kogan et al. prepared amphipathic peptide (CLPFFD)-modified gold nanorods loaded with siRNA against poly(ADP-ribose) polymerase 1 (PARP-1).^583^ CLPFFD is a β-amyloid-derived peptide that facilitates vector delivery across the BBB to the brain.^584,585^ Perinatal asphyxia (PA) activates PARP-1,^586^ which may impair stressed cells.^587^ CLPFFD-modified gold nanorods deliver siPARP-1 to the brain and downregulate PARP-1 expression in the mesencephalon and hippocampus of asphyxia-exposed rats. Fragile X syndrome (FXS), a single mutant type of autism-related disorder, is caused by a repeat expansion mutation on the fragile X mental retardation 1 (FMR1) gene.^588^ Therefore, CRISPR-based editing technology is particularly suitable for the treatment of FXS. Metabotropic glutamate receptor 5 (mGluR5) gene (Grm5) is a potential target for CRISPR-based FXS therapy.^589,590^ AuNPs loaded the RNA-guided endonucleases Cas9 and Cpf1 and delivered them to the brain via stereotaxic-guided local delivery. A single dose could inhibit 40~50% of the mGluR5 gene in the striatum.^591^ Bcl2Like12 (Bcl2L12) is an oncogene consistently highly expressed in GBM^592,593^ and inhibits caspase-3 and caspase-7 activation by directly interacting with pro-caspase-7^594^ and upregulating small heat shock protein and procaspase-3-specific inhibitor.^595^ Stegh et al. conducted a first-in-human early phase 1 clinical trial involving the systemic administration of siBcl2L12-loaded AuNPs (NU-0129) in recurrent GBM patients (NCT03020017).^596^

Silicon-based inorganic nanoparticles are also widely applied in biomedicine and drug delivery. Ruoslahti et al. isolated and identified a peptide (CAQK) that specifically targeted brain injury sites. siRNA was loaded on the CAQK-modified porous silicon NPs (pSiNPs). After intravenous injection, the vectors specifically accumulated at the brain injury site of penetrating brain-injured mice and downregulated the expression of the target gene, providing a strategy for treating traumatic brain injury (TBI).^597^ Sailor et al. added siRNA and Ca^2+^ to pSiNPs. Ca^2+^ reacted with soluble silicic acid released from the porous structure of pSiNPs to form insoluble calcium silicate shells. siRNA was encapsulated in the porous structure of Ca-pSiNPs during calcium silicate formation (Ca-pSiNP-siRNA), thereby slowing its release rate. Ca-pSiNP-siRNA modified with RVG29 and Mtp (myr-GWTLNSAGYLLGKINLKALAALAKKIL) delivered siRNA to the brain injury site in mice and downregulated the expression of peptidylprolyl isomerase B (PPIB) after stereotactic local administration.^598^ Consistent results were observed by replacing calcium silicate with graphene oxide nanosheets to encapsulate siRNA.^599^ PEI can also encapsulate pSiNPs-loaded siRNA, which protects the siRNA and avoids the cellular uptake concerns associated with the negative charge of nanoparticles. Multidrug resistance-associated protein 1 (MRP1) is overexpressed in GBM^600^ and actively removes drugs in the cytoplasm by hydrolyzing ATP,^601,602^ causing the multidrug-resistant phenotype of GBM. PEI-coated pSiNPs delivered siMRP1 to GBM and downregulated MRP1 expression, thereby enhancing the sensitivity of GBM to chemotherapy.^603^

Inorganic nanoparticles containing Mn, Co, and/or Fe elements commonly exhibit magnetic properties^604–606^ and have been developed as drug vectors.^607–609^ Magnetic iron oxide nanoparticles (MIONs) have been applied for MRI and magnetic field-guided drug delivery due to their excellent magnetic properties.^610–612^ MIONs and cationic polymer-hybridized nanoparticles have been prepared to load and deliver nucleic acid drugs. Zhang et al. compared MIONs coated with polyarginine (PArg), PLys, and PEI. PArg-coated MIONs exhibited the lowest toxicity and highest transfection efficiency, possibly due to the biodegradable polymer’s high positive charge density.^613^ Layered double hydroxide (LDH) nanoparticles are inorganic nanoparticles with positively charged surfaces that have low toxicity, high cellular uptake efficiency, and a weak immune response and are applied for nucleic acid drug delivery.^614–616^ Xu et al. prepared pH-sensitive Mn-based LDH (Mn-LDH) nanoparticles to efficiently deliver cell-death siRNA into mouse neuroblastoma N2a cells and inhibit the proliferation of tumor cells.^617^ Magnetoelectric nanoparticles (MENPs) are multiferroic materials with significant magnetic and electric field coupling capabilities.^618^ Nair et al. applied MENPs for loading and delivering siBeclin1.^619^ Autophagy is an essential target in the HIV cycle. Beclin1 is a critical protein that regulates the autophagic pathway and promotes inflammation and viral replication in HIV-1-infected microglia.^620,621^ The MENPs’ movement was controlled by applying a weak current of magnetic force, thereby crossing the in vitro BBB model and attenuating viral replication and virus-induced inflammation, a promising treatment for HIV-associated neurocognitive disorders (HAND). The researches on inorganic nanoparticle-based nucleic acid drug vectors have been listed in Table 4.

**Table 4:** Inorganic nanoparticle-based vectors for nucleic acid drug delivery

Inorganic nanoparticle	Organic modification	Disease	Nucleic acid drug	Therapeutic target	Ref(s)
Gold nanorods	CLPFFD peptide	Perinatal asphyxia	siRNA	PARP-1	^583^
AuNPs	DNA-SH	FXS	CRISPR RNPs	Grm5	^591^
AuNPs		GBM	siRNA	Bcl2L12	^596^
pSiNPs	CAQK peptide	TBI	siRNA		^597^
Ca-pSiNP	RVG29 and Mtp peptide	Brain injury	siRNA	PPIB	^598^
Graphene oxide nanosheets	RVG29 peptide	Brain injury	siRNA	PPIB	^599^
pSiNPs	PEI	GBM	siRNA	MRP1	^603^
MIONs	PArg, PLys, and PEI				^613^
Mn-LDH			siRNA		^617^
MENPs		HIV-1 replication and viral-induced inflammation	siRNA	Beclin1	^619^

### Nucleic acid drug conjugates

Nucleic acid drug conjugates offer unique advantages for clinical translation. Nucleic acid drug conjugates are prepared by chemical synthesis, have a simple and well-defined molecular structure and a single component, and therefore have almost absolute homogeneity and almost no batch-to-batch variation. These strengths are highly supportive of the clinical translation of nucleic acid drugs. In addition, the stability of nucleic acid drug conjugates facilitates their storage and transport compared to other vectors. Moreover, nucleic acid drug conjugates do not have the concern of premature drug leakage during in vivo delivery of traditional drug vectors. Furthermore, the amount of additives in nucleic acid drug conjugates is significantly less than that of other vectors, thus diminishing the potential side effects of additives. Finally, nucleic acid drug conjugates are small enough to allow systemic delivery of drugs in vivo by non-intravenous means, such as subcutaneous administration, which is more clinically acceptable. However, the preparation of nucleic acid drug conjugates relies on chemical synthesis. Only synthetically available nucleic acid drugs, such as siRNA and ASOs, are capable of conjugating functional molecules. In addition, small-size and nucleic acid drug conjugates must also account for their rapid renal clearance. Moreover, endosomal/lysosomal escape of nucleic acid drug conjugates is a challenge to overcome urgently.

Cellular uptake is the primary challenge for nucleic acid drug conjugates due to their negative charges and strong polarity. Cholesterol has a lipophilic cyclic structural framework and is more efficient than straight-chain fatty acids. Burgess et al. conjugated cholesterol on siHTT and temporarily opened the BBB by MRgFUS, which improved the BBB permeation efficiency of cholesterol-siHTT. Cholesterol-siHTT efficiently silenced HTT and contributed to the treatment of HD.^622^ α-Tocopherol also has a lipophilic cyclic structural framework. Yokota et al. conjugated cholesterol or α-tocopherol to DNA/RNA heteroduplex oligonucleotide (HDO).^623^ They selected DMPK as the target gene. Amplification of the CAG repeat in the DMPK gene causes myotonic dystrophy type 1 (DM1). Silencing DMPK contributes to DM1 treatment.^624,625^ Cholesterol-HDO and α-tocopherol-HDO significantly promoted HDOs across the BBB and silenced DMPK genes in the CNS. In contrast, neither cholesterol-ASO nor cholesterol-siRNA exhibited a decrease in DMPK expression in the CNS, which suggested that HDOs were also necessary for conjugates to enter the brain from the periphery.

Ligand modifications can promote drug crossing of the BBB or cellular uptake via RMT or RME. Folic acid is a typical small molecule ligand. Folic acid-ASO against miRNA-21 promoted drug uptake by tumor cells and contributed to the treatment of GBM.^626^ Bortolozzi et al. then conjugated sertraline on siRNA for the treatment of depression.^233^ SERT affects the depressive process by controlling the transport and reuptake of serotonin and is the target of most prescribed antidepressants, such as selective serotonin reuptake inhibitors (SSRI) and selective serotonin and norepinephrine reuptake inhibitors (SNRI).^627,628^ Sertraline is an SSRI that specifically targets SERT on the surface of diseased neurons. Serotonin-siSERT was efficiently delivered to the brain after intranasal administration and was internalized by serotonergic neurons specifically. Serotonin-siSERT inhibited serotonin reuptake by downregulating SERT for treating major depressive disorder. DHA was also employed to prepare siRNA conjugates, facilitating the crossing of the BBB and targeting neurons, and enhancing the hydrophobicity of siRNA, thus facilitating the cellular uptake of DHA-siRNA.^629,630^ Peptides are also common ligand molecules. Ji et al. conjugated (cyclo(RGD-D-FK)-Ahx)_2_-E (biRGD)-PEG to siRNA.^631^ The PEG modification improved the resistance of siRNA to protein adsorption in circulation. biRGD modification promoted the targeted uptake of siRNA by GBM cells. Phosphatidylinositol-4,5-biphosphate 3-kinase catalytic subunit b (PIK3CB), the catalytic subunit p110 b of the PI3K gene family, was associated with high risk and low survival of GBM.^632–635^ biRGD-PEG-siPIK3CB significantly downregulated PIK3CB expression, which contributed to the treatment of GBM. Monoclonal antibodies (mAbs) are also used as ligand molecules for conjugation with nucleic acid drugs. DRR, the established genetic driver of GSC migration, can promote GBM migration by regulating the focal adhesion kinetics of migrating cells at the leading edge and activating the AKT signaling pathway.^636–639^ CD44 mAb conjugated double-stranded ASO (dsASO) against downregulated in renal cell carcinoma (DRR) could be efficiently accumulated in GBM and treated GBM by downregulating DRR.^640^

Polymers can modify multiple molecules while conjugating nucleic acid drugs due to their large number of repeating side chains, giving nucleic acid drug conjugates versatility. Holler et al. employed poly(β-L-malic acid) (PBMA) as a substrate to conjugate peptides (LLL) for endosomal/lysosomal escape, TfR monoclonal antibodies for targeting BBB and GBM, and ASO against laminin-411 by disulfide bonds for GBM treatment.^641^ These conjugates thus possessed the functions of crossing the BBB, efficient accumulation in GBM, endosomal/lysosomal escape, and GSH-mediated ASO release. Laminin-411, a tumor-specifically vascular basement membrane protein, is overexpression in GBM, promotes GBM recurrence, and shortens patients’ survival.^642,643^ These two released ASOs against laminin α4 and β1 chains blocked laminin-411 synthesis, inhibiting neovascularization and GBM proliferation.

The siRNA conjugated with hydrophobic ligands, such as cholesterol and DHA, can improve the cellular uptake of the drug. However, the conjugates are poorly diffusible in the brain and remain mainly at the injection site.^551,644^ PS backbone can promote the wide distribution of ASOs in the CNS.^645^ Inspired by this, Khvorova et al. modified PS on siRNA to facilitate drug distribution in the whole brain. However, siRNA modified with a small amount of PS resulted in limited pro-diffusion, while a large amount of PS modification reduced the gene silencing efficiency^646,647^ and increased toxicity.^648,649^ To overcome this limitation, they synthesized PS-containing divalent siRNA conjugates (di-siRNA), two PS-containing siRNAs conjugated by a linker. A single dose of di-siRNA against HTT could silence HTT mRNA and protein throughout the brain for more than six months after injection into the cerebrospinal fluid.^650^

## Nucleic acid drug-based combination therapy for brain diseases

**Fig. 6:**
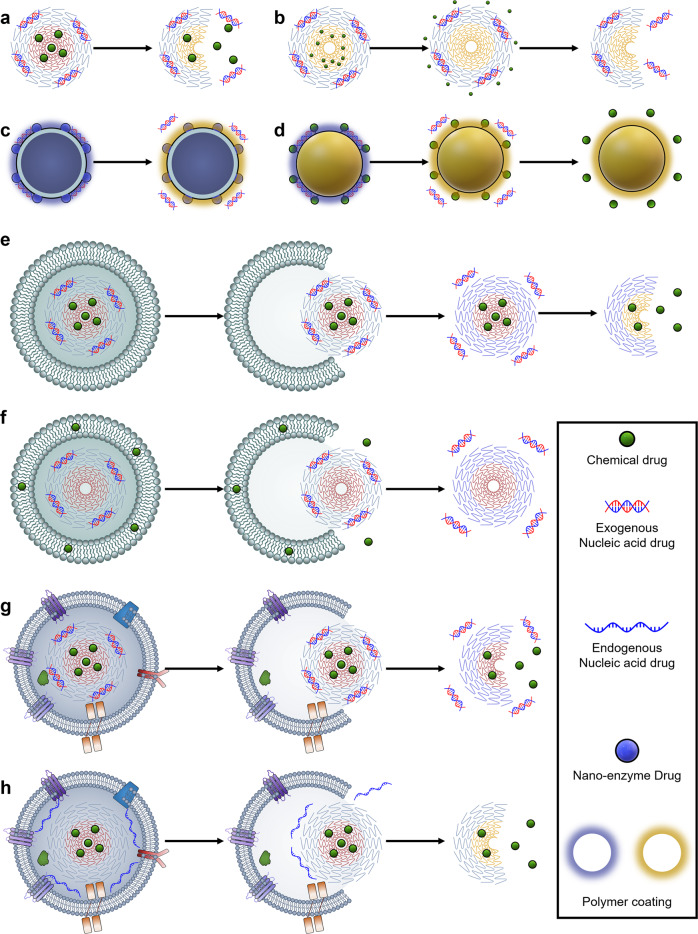
Schematic illustration of co-loading and release of multi drugs. **a** The degradation of PNPs promotes the co-release of nucleic acid drugs and chemical drugs. **b** The covalent bonds linking the chemical drugs and PNPs were cleaved to mediate the pioneering release of the chemical drugs. The chemical drug release further mediates the degradation of PNPs and the release of nucleic acid drugs. **c** The charge transition of the vector surface mediates the release of nucleic acid drugs. The nano-enzyme drugs do not need to be released. **d** The charge transition of the vector surface mediates the pioneering release of nucleic acid drugs. The covalent bonds linking the chemical drugs and vectors were cleaved to mediate the subsequent release of the chemical drugs. **e** The degradation of the lipid shells promotes the PNP release. The charge transition of the cationic outer layer of PNPs mediates the pioneering release of nucleic acid drugs. The disease microenvironment mediates the conversion of the inner core of PNPs from hydrophobic to hydrophilic, thus facilitating the degradation of PNPs and the subsequent release of the chemical agent. **f** Degradation of the lipid outer membrane mediates the pioneering release of the chemical agent. The charge transition of the cationic outer layer of PNPs mediates the subsequent release of nucleic acid drugs. **g** The exosome outer membrane fuses with the cell membrane to deliver the PNPs directly to the cytoplasm. The disease microenvironment mediates the charge transition in the outer layer and the hydrophile transition in the hydrophobic inner core of PNPs, facilitating the co-release of chemical and nucleic acid drugs. **h** The exosome outer membrane fuses with the cell membrane to deliver the endogenous nucleic acid drugs and PNPs directly to the cytoplasm. The hydrophile transition in the hydrophobic inner core of PNPs mediates the subsequent release of the chemical drugs

Brain diseases, especially GBM and NDs, are complex diseases with multiple therapeutic targets. The mono-drug may not be efficient in treating these complex diseases. In addition, different types of drugs against the same therapeutic target possess unique advantages. Multidrug-loaded vectors are expected to enhance the therapeutic efficacy of brain diseases through combination therapies. In this section, we will focus on designing and preparing vectors for the co-loading and release of multi-drugs (Fig. 6) and the mechanisms of synergistic treatment of brain diseases.

### TMZ and nucleic acid drugs for GBM combination therapy

TMZ, an alkylator-type antineoplastic agent, is currently the most widely used first-line chemotherapeutic agent for GBM and a critical component of the current standard treatment protocol for GBM.^651–653^ However, the resistance of primary and recurrent GBM to TMZ severely limits the chemotherapeutic efficacy of TMZ for GBM. Enhancing the sensitivity of GBM to TMZ by combination therapy is expected to improve the chemotherapeutic efficacy of GBM. Nucleic acid drugs currently enhance the chemotherapeutic efficacy of TMZ through three main strategies: inhibiting GBM resistance to TMZ, modulating the immune microenvironment of GBM, and blocking other therapeutic targets of GBM.

*O*^6^-methylguanine-DNA-methyltransferase (MGMT) is a primary factor in TMZ resistance.^654,655^ MGMT can repair damaged DNA by demethylation, thereby diminishing the anti-tumor effects of TMZ.^656^ The recurrent GBM is more insensitive to TMZ than the primary GBM due to higher MGMT expression.^657^ siMGMT can attenuate GBM resistance to TMZ by directly downregulating MGMT expression. Stegh et al. loaded siMGMT on AuNPs and combined siMGMT-loaded AuNPs with TMZ to treat GBM. siMGMT silenced MGMT expression in GBM, thereby enhancing GBM sensitivity to TMZ.^658^ In addition, STAT3, a major gene responsible for tumor cell proliferation and resistance to apoptosis,^659^ can enhance GBM resistance to TMZ by upregulating MGMT expression.^660^ Therefore, downregulating STAT3 expression can also indirectly improve the sensitivity of GBM to TMZ. Shi et al. coupled hydrophobic poly(N-isopropylacrylamide) (PNIPAM) to siSTAT3 via disulfide bonds.^661^ PNIPAM-SS-siRNA self-assembled into spherical nucleic acids (SNAs) and encapsulated hydrophobic TMZ in its hydrophobic core. The SNAs can cross the BBB and promote cellular uptake of GBM cells via cell-membrane-bound scavenger receptor-based RMT and RME, respectively.^662,663^ GSH in the GBM cytoplasm cleaved the disulfide bonds, facilitating the co-release of siRNA and TMZ, thereby synergistically treating GBM. Shi et al. also co-loaded siSTAT3 and TMZ via exosomes.^664^ Heme oxygenase 1 (HMOX1) is highly expressed on GBM cell membranes.^665^ Chemotherapy further promotes the expression of HMOX1 and may promote GBM resistance to chemotherapy.^665–668^ Therefore, the HMOX1 targeting peptide (HSSP, VQDSAPVETPR) could be employed as a ligand to target GBM. TMZ and siSTAT3 were encapsulated into MSC-derived exosomes by sonication. Cysteine-modified HSSP was coupled to DSPE-PEG via a Michael addition reaction (DSPE-PEG-HSSP), which facilitated the insertion of HSSP onto the exosome surface via hydrophobic interaction. MSC-derived exosomes and HSSP modification facilitated the crossing of the BBB and the targeting of the GBM, respectively.

The immunosuppressive microenvironment is also an essential factor in the poor therapeutic outcome of GBM.^669^ Infiltrating immune cells, such as microglia, myeloid suppressor cells, granulocytes, macrophages, and T lymphocytes, maintain immune homeostasis in the brain.^670,671^ These immune cells can recognize and eliminate tumor cells, thereby inhibiting tumor progression. However, GBM may evade the immune system’s surveillance through multiple mechanisms.^672,673^ Therefore, maintaining immune homeostasis in the brain is a strategy for synergistic treatment with anti-tumor agents.

Activation of the PD-1/PD-L1 signaling pathway induces T effector (Teff) cells apoptosis in GBM^674,675^ and is the main driver of the tumor immunosuppressive microenvironment.^676–678^ In addition, PD-L1 mediates resistance to TMZ in GBM via the AMPK/ULK1 pathway.^679^ The expression levels of PD-1 and PD-L1 were higher in recurrent GBM than in primary GBM.^680^ Zhou et al. co-loaded siPD-L1 and TMZ via LPNPs.^681^ PAsp-g-PEI/dsb was synthesized by grafting PEI onto PAsp via disulfide bonding. PAsp-g-PEI/dsb formed complexes with siPD-L1 via electrostatic interaction. Subsequently, this complex and TMZ are encapsulated by DOPE, cholesterol, and the ligand-modified lipid molecule DSPE-PEG-Glu to form LPNPs, thus co-loading siPD-L1 and TMZ. Glu and disulfide bonds impart to these LPNPs the ability to target GBM and GSH-stimulate drug release, respectively. Interestingly, disulfide bond breakage depletes GSH, thereby elevating the amount of ROS in GBM.^682,683^ Upregulation of ROS can successively downregulate β-catenin and MGMT,^684,685^ thus enhancing the sensitivity of GBM to TMZ. In addition, CD47, an immune checkpoint protein highly expressed in GBM cells,^686,687^ can inhibit macrophage phagocytosis of GBM cells by binding to the signal-regulatory protein α (SIRPα) receptor of macrophages.^688,689^ Downregulation of CD47 promotes immune clearance of GBM cells by blocking the interaction between CD47 and the SIRPα receptor. Liu et al. designed a functional group N^1^, N^3^-dicarbamimidoylisophthal amide (BGG) capable of high affinity for nucleic acid molecules through multiple forces according to computer molecular docking experiments. Generation 5 BGG (G5-BGG) is a BGG-modified dendrimer. G5-BGG first forms a complex with the shCD47-encoded pDNA and subsequently forms hydrogels with poly(lactic-co-glycolic acid) (PLGA)-PEG-PLGA. Ester bonds could degrade the hydrogels after delivery to GBM via stereotaxic-guided local administration, and the released pDNA/G5-BGG was taken up by GBM cells. pDNA improved the immune microenvironment of GBM by downregulating CD47, which facilitated GBM treatment with TMZ.^690^ Moreover, TGF-β inhibits the proliferation of T and B cells and promotes the proliferation of T regulatory cells,^691,692^ affecting the immune homeostasis in the brain. Downregulation of TGF-β expression can modulate the GBM immune microenvironment by regulating naive T cells.^693,694^ Our laboratory prepared LPNPs to co-encapsulate TMZ and siTGF-β.^183^ First, cationic poly((2-acryloyl)ethyl(p-boronic acid benzyl)diethylammonium bromide) (BA-PDMAEA) and siTGF-β formed PNPs by electrostatic and hydrophobic interaction. Subsequently, the PNPs were encapsulated in the core of liposomes composed of DSPE-PCB-Ang and cholesterol, while the TMZ was encapsulated in a hydrophobic bilayer of liposomes. Ang facilitated vectors crossing the BBB and accumulating in GBM cells. PCB ensured anti-protein adsorption, avoided ABC in circulation, and facilitated vectors escaping from endosomes/lysosomes and releasing TMZ. High levels of ROS in GBM cells mediated the charge conversion of BA-PDMAEA and promoted the release of siRNA. TMZ and siTGF-β synergistically treated GBM.

MALAT1, a cancer-promoting lncRNA that promotes proliferation and metastasis of tumor cells,^695^ is highly expressed in GBM.^696^ Chang et al. encapsulated siMALAT1 in TfRscFv-modified liposomes to promote siRNA crossing the BBB and accumulating in GBM. TMZ was also employed for GBM combination therapy by intravenous injection.^697^ In addition, p53 is a tumor suppressor that can prevent the development and progression of GBM.^698^ p53-related miRNA dysregulation impairs the tumor suppressive effect of p53 and increases the resistance of GBM to chemotherapy. miR-21 high expression in GBM^699–701^ and miR-100 low expression in GBM^702,703^ promote GBM proliferation by interfering with the anti-tumor effect of p53. Therefore, inhibiting miR-21 and increasing the expression of miR-100 can improve the sensitivity of GBM to chemotherapeutic drugs. Paulmurugan et al. modified chitosan-cyclodextrin on the surface of gold-iron oxide nanoparticles, thereby adsorbing anti-miR-21 and miR-100 via electrostatic interactions. The T7 CPP modification promoted vector accumulation in the brain after intranasal administration.^704^ Paulmurugan et al. also co-loaded anti-miR-21 and miR-100 via exosomes.^705^ They encapsulated anti-miR-21 and miR-100 into NSC-derived EVs and modified the EVs’ surface with C-X-C chemokine receptor type 4 (CXCR4). CXCR4 was able to be specifically taken up by GBM through one-to-one receptor-ligand stoichiometry with stromal-derived-factor-1 (SDF-1),^706^ which was highly expressed in GBM cells.^707^ After intranasal administration, EVs efficiently deliver anti-miR-21 and miR-100 into GBM cells with a combination of NSC-derived EVs’ potential tropism for GBM^708^ and CXCR4-based RME, improving the therapeutic effect of TMZ.

### DOX and nucleic acid drugs for GBM combination therapy

DOX is a broad-spectrum anti-tumor agent widely applied in clinics and can be employed in chemotherapy for diverse tumors. However, DOX has the problem of GBM resistance. Nucleic acid drugs can downregulate the therapeutic targets of tumors at the genetic level and overcome the challenge of GBM resistance. In addition, since both DOX and nucleic acid drugs possess broad-spectrum advantages, these combination therapy strategies are expected to be applied to other tumors.

Activation of anti-apoptotic proteins can reduce tumor sensitivity to chemotherapy.^709^ Bcl-2 is the major anti-apoptotic protein that inhibits tumor cell apoptosis by inhibiting the release of apoptosis-inducing factors and cytochrome c from mitochondria.^710,711^ Shuai et al. loaded siRNA and DOX through PEI-based PNPs with folic acid modification for GBM combination therapy.^712^ As mentioned previously, the high expression of miR-21 contributes to drug resistance and the proliferation of GBM. Kang et al. synthesized PDMAEMA-based amphiphilic hyper-branched star-copolymers to load DOX and anti-miR-21 through hydrophobic and electrostatic interactions, respectively. PNPs co-loaded with DOX and miRNA were applied for the combination therapy of GBM after intravenous administration.^713^ Organic-inorganic hybrid nanoparticles can also co-load nucleic acid drugs and DOX after functionalized modifications. Chen et al. loaded DOX through Se nanoparticles (SeNPs). Subsequently, ligand-attached cationic polymers were modified on the surface of the SeNPs to adsorb sic-myc electrostatically.^714^ In addition, they established approaches to modulate the morphology and size of SeNPs through biomolecules,^715,716^ which could contribute to the application of such organic/inorganic hybrid nanoparticles for combination therapy to other tumors.

CRISPR/Cas13a has been developed for GBM combination therapy. CRISPR technology has powerful gene editing and silencing efficacy, but its safety has been a concern. Cheng et al. designed hierarchical self-uncloaking CRISPR-Cas13a-customized RNA nanococoons to load, deliver, and programmatically release Cas13a RNP and DOX for GBM combination therapy.^717^ They first prepared RNA nanosponges by rolling circle transcription. These RNA nanosponges consisted of three functional motifs: a multivalent aptamer targeting GBM, cholesterol-stabilizing nanosponges and promoting cellular uptake, and a trans-cleavage substrate for Cas13a-mediated degradation. Cas13a RNP was encapsulated in cationic pH-responsive nanocapsules to limit the activity of Cas13a before accumulating in the GBM. Subsequently, DOX and the nanocapsules were loaded on the interior and surface of the RNA nanosponges, respectively, forming RNA nanococoons. The acidic environment of the endosome/lysosome promoted the degradation of the nanocapsules and the release of Cas13a RNP, thus achieving the first self-uncloaking of the vectors. The released Cas13a recognized and cleaved the trans-cleavage substrate in the RNA nanosponges to release DOX, enabling the second self-uncloaking of the vectors. EGFR variant III (EGFRvIII) is highly expressed in GBM and leads to chemoresistance by promoting DNA repair.^718,719^ Prior-released Cas13a RNP enhanced the sensitivity of GBM to the subsequent release of DOX by silencing EGFRvIII.

### Other chemical drugs and nucleic acid drugs for GBM combination therapy

PTX inhibits tumor cell mitosis by mediating abnormal microtubule function, thereby promoting apoptosis.^720,721^ Matrix metallopeptidase-2 (MMP-2) is highly expressed in GBM.^722^ Activation of MMP-2 contributes to GBM invasion by promoting tumor neovascularization.^723,724^ Wang et al. encapsulated the PTX and complexes formed by RNAi pDNA against MMP-2 with PEI in PLGA microfibers.^725^ The microfibers synergistically treated GBM through stereotactic local administration. Stathmin, a microscopic regulatory protein, mediates the resistance of GBM to nitrosourea anti-tumor agents.^726^ Park et al. prepared a pH-sensitive polymer to conjugate sistathmin via disulfide bonds. The sistathmin conjugates were electronegative by deprotonation at physiological pH, while in the acidic endosomal/lysosomal microenvironment, they were converted to a compact lipid membrane disrupted state by protonation, contributing to endosome/lysosome escape.^727^

Suicide gene therapy, a typical oncology combination therapy strategy, involves Tf genes encoding bacterial or viral enzymes to tumor cells to convert inert prodrugs into anti-tumor agents. For example, Herpes simplex virus-thymidine kinase (HSV-TK) can phosphorylate inert ganciclovir (GCV) into GCV triphosphate with anti-tumor activity. In addition, HSV-TK/GCV has a bystander effect, transporting the anti-tumor active HSV-TK triphosphate to adjacent tumor cells, thereby enhancing the therapeutic efficacy of GBM.^728^ Chen et al. used PEI-based PNPs with Ang modification for targeted delivery and combination therapy of HSV-TK pDNA and GCV to GBM.^729^ The tumor necrosis factor-related apoptosis-inducing ligand (TRAIL) can enhance the anti-tumor activity of HSV-TK/GCV.^730^ Chen et al. then transfected MSCs with TRAIL and HSV-TK genes via cationic PNPs for the combination therapy of GBM.^731^

### Other nucleic acid drug-based GBM combination therapy

Different nucleic acid drugs have been applied in the combination therapy of GBM. Oct4 and SOX2 can induce a GSC state in GBM cells through the downregulation of the miRNA network. miR-148a and miR-296-5p inhibition is required for the ability of Oct4/SOX2 to induce GBM tumor proliferation.^732,733^ Green et al. prepared PBAE-based biodegradable PNPs to load miR-148a and miR-296-5p. The miRNA-loaded PNPs inhibited GBM proliferation and synergistically enhanced the response to standard-of-care γ radiation after local administration.^734^ Roundabout homolog 1 (Robo1),^735^ yes-associated protein 1 (YAP1),^736^ sodium-potassium-chloride cotransporter (NKCC1),^737^ survivin,^738^ and EGFR^739^ are all tumor therapeutic targets. Green et al. also encapsulated siRNAs against these anti-GBM genes in the same PBAE-based biodegradable PNPs for GBM combination therapy.^740^ Nucleic acid drugs can treat GBM through targeted therapy, such as siPLK1 and siEGFR, and immunotherapy, such as siPD-L1 and siCD47, which means vectors loaded with multiple nucleic acid drugs can be applied to treat GBM by combining targeted therapy and immunotherapy. Since PD-L1 upregulation and EGFR activation correlate,^741,742^ Tannous et al. loaded siPD-L1 and siEGFR in iRGD-modified solid lipid nanoparticles to perform combined targeted and immunotherapy against GBM.^743^ Interestingly, short bursts of radiation therapy were introduced to activate neutrophil infiltration and thus mediate vector crossing of the tumor vascular barrier.^744^ In addition, short bursts of radiation facilitated vectors accumulating in GBM via a tumor-associated macrophage-dependent manner.^745,746^ The researches of multiple drugs containing nucleic acid drugs for the synergistic treatment of brain tumors have been summarized in Table 5.

**Table 5:** Multi-drugs containing nucleic acid drugs synergistically loaded nanoparticles for brain tumor therapy

Vectors	Nucleic acid drug	Therapeutic target	Nucleic acid drug release	Other drug (s)	release	Ref(s)
Inorganic nanoparticles	siRNA	MGMT		TMZ		^658^
PNPs	siRNA	STAT3	GSH cleavage	TMZ	GSH cleavage	^661^
Exosome	siRNA	STAT3		TMZ		^664^
LPNPs	siRNA	PD-L1	GSH cleavage	TMZ	GSH cleavage	^681^
Dendrimer	shRNA	CD47	Ester bonds degrade	TMZ		^690^
LPNPs	siRNA	TGF-β	ROS mediated charge conversion	TMZ	ROS	^183^
Liposomes	siRNA	MALAT1		TMZ		^697^
Organic-inorganic hybrid nanoparticles	miRNA	anti-miR-21 and miR-100		TMZ		^704^
Exosomes	miRNA	anti-miR-21 and miR-100		TMZ		^705^
PNPs	siRNA	BCL-2		DOX		^712^
PNPs	miRNA	anti-miR-21		DOX		^713^
Organic-inorganic hybrid nanoparticles	siRNA	c-myc		DOX		^714^
Nanococoons	CRISPR-Cas13a RNP	EGFRvIII	pH-responsive	DOX		^717^
PNPs	pDNA	MMP-2		PTX		^725^
Conjugates	siRNA	stathmin	pH-sensitive	Carmustine	GSH cleavage	^727^
PNPs	pDNA	HSV-TK		GCV		^729^
PNPs	pDNA	TRAIL and HSV-TK		GCV		^731^
PNPs	miRNA	miR-148a and miR-296-5p	GSH cleavage			^734^
PNPs	siRNA	Robo1, YAP1, NKCC1, EGFR, and survivin				^740^
Solid lipid nanoparticles	siRNA	PD-L1, EGFR				^743^

### Nucleic acid drug-based AD combination therapy

AD is the most prevalent ND with cognitive and memory decline clinical features. Abnormal aggregation of extracellular Aβ^311,747^ and intracellular accumulation of hyperphosphorylated tau protein^748,749^ are the key pathological features of AD and lead to neuronal loss. In addition, abnormal Aβ accumulation leads to abnormal microglia function to induce the production of multiple pro-inflammatory mediators such as interleukin 1β (IL-1β), tumor necrosis factor α (TNF-α), and ROS,^750^ which in turn promote the production and aggregation of Aβ,^751^ resulting in a vicious circle. Therefore, AD is a complex disease with multiple therapeutic targets and is well suited for nucleic acid drug-based combination therapy.

As mentioned previously, BACE1 is the initiating and rate-limiting enzyme of Aβ biosynthesis and is a widely applied target gene for inhibiting Aβ regeneration. Aβ regeneration inhibition by nucleic acid drugs and the removal of existing Aβ in AD lesions by other drugs simultaneously is an ideal strategy for AD combination therapy by targeting Aβ. Rapamycin can clear Aβ by promoting microglia-mediated autophagy activation.^752^ Curcumin can reduce the production of pro-inflammatory cytokines by inhibiting the NF-κB pathway, thereby inhibiting the production and accumulation of Aβ.^753^ Fluvastatin can also promote Aβ clearance.^754^ Gao et al. co-loaded siBACE1 and rapamycin through functionalized cationic dendrigraft PLys (DGL).^155^ DGL that was modified with Aleuria aurantia lectin (AAL) and KLVFF peptide facilitated the vectors entering the brain through the nasal cavity and targeting Aβ, respectively. In addition, KLVFF could also inhibit Aβ aggregation and accelerate Aβ clearance.^755,756^ siBACE1 and rapamycin synergistically treated AD by inhibiting Aβ regeneration and promoting microglia-mediated Aβ clearance, respectively. Ding et al. then prepared lipid-based nanoparticles co-loaded with siBACE1 and curcumin.^757^ Interestingly, phosphatidic acid-functionalized high-density lipoprotein (pHDL) was added to the nanoparticles to improve the permeation efficiency across the BBB and the interaction with Aβ.^758^ In addition, siRNA was modified with cholesterol for anchoring pHDL on the vectors.^759^ Curcumin and siRNA were co-delivered to the brain to synergistically treat AD by mediating microglia normalization, accelerating Aβ clearance, and reducing Aβ production. Our laboratory downregulated BACE1 by CRISPR/Cas9 technology and cleared Aβ by fluvastatin.^192^ Fluvastatin and Cas9-sgBACE1 pDNA were loaded in PLys-modified nano-biohybrid complexes via ester bond and electrostatic interactions, respectively. The RVG29 modification promoted vectors to cross the BBB and target neurons. Protease and esterase-mediated release of pCas9-sgBACE1 pDNA and fluvastatin were used to synergistically treat AD by knocking down the BACE1 gene and scavenging Aβ, respectively. In addition, CRISPR/Cas-based gene editing technology was able to downregulate BACE1 expression and Aβ regeneration in a long-lasting manner.

Microglia are the resident immune cells in the CNS that continuously monitor and clear pathogens and cellular debris in the brain.^760^ Microglia can clear Aβ in AD patients’ brains,^761^ but the abnormal aggregation of Aβ can interfere with the normal function of microglia.^750,762^ Therefore, modulating dysfunctional microglia is also an ideal strategy for the combined treatment of AD. Our laboratory developed PNPs co-loaded with siSTAT3, fingolimod, and ZnO nanoparticles.^235^ Mannose analog modifications were able to promote PNPs to cross the BBB and target microglia.^763^ Fingolimod reduced inflammatory mediators in microglia.^764^ siSTAT3 downregulated the high expression of STAT3 in abnormally functioning microglia and promoted normal microglial function. ZnO nanoparticles promoted the normalization of microglia polarization by enhancing the TH2 response.^765^ The chemical drug, nucleic acid drug, and inorganic nanoparticle drug could synergistically promote the normalization of microglia and contribute to the combination therapy of AD.

Intracellular phosphorylated tau protein is also a key target for AD therapy. Our laboratory proposed a strategy for AD combination treatment by targeting extracellular Aβ, intracellular phosphorylated tau protein, and the high ROS microenvironment.^238^ “Cascaded rocket” PNPs with spatiotemporal separation were prepared for co-delivery of PHis, salsalate, and siNF-κB. Modifications of DAG and Tet-1 peptides conferred the ability of PNPs to cross the BBB and target neurons, respectively. Transition-metal ions in AD lesions were chelated by PHis, enabling the first separation stage by shedding PHis from PNPs.^766,767^ PHis inhibited the production of toxic Aβ aggregates by competitive chelation with transition-metal ions.^768^ After neuron uptake, cathepsin B mediated the release of salsalate for secondary segregation. The released salsalate degraded phosphorylated tau by decreasing acetyltransferase p300 levels^769,770^ and mediated the degradation of PNPs for tertiary segregation. The released siNF-κB reduced the level of oxidative stress in AD neurons by downregulating NF-κB.^771,772^ The polymeric, small molecule, and nucleic acid drugs synergistically treated AD via Aβ aggregates, phosphorylated tau protein, and ROS, respectively.

Neuronal loss is the direct pathogenesis of AD.^773^ Replenishing healthy neurons or promoting neuronal self-restoration is promising for treating AD. NSC transplantation is an ideal strategy for treating AD by replenishing neurons due to their non-immunogenicity,^774^ high differentiation potential,^775,776^ and ability to target migration to the disease site.^777^ However, the multi-differentiation orientation of NSCs makes it challenging to differentiate enough NSCs. Retinoic acid can induce directed differentiation of NSCs into neurons,^778^ but SOX9 protein counteracts the effect of retinoic acid.^779^ Therefore, prior inhibition of SOX9 expression is critical for retinoic acid to exert its directional differentiation role. Our laboratory prepared PCB-based PNPs to load retinoic acid and siSOX9 via ester bonding and electrostatic interactions, respectively.^228^ CPP modification facilitated the uptake of PNPs by NSCs. pH-sensitive PCB mediated the rapid release of siSOX9 for premature downregulation of SOX9. Esterase-mediated retinoic acid’s slow and sustained release promoted the directed differentiation of NSCs into neurons. PNPs-treated NSCs were injected into the brain under the guidance of stereotaxic localization and achieved effective AD treatment through directed differentiation to neurons.

The neurogenesis and directed differentiation of endogenous NSCs also contribute to self-repair after neuronal loss.^780,781^ BDNF can promote the proliferation of NSCs to enhance neural repair.^782^ Our laboratory proposed a strategy to increase the concentration of BDNF in AD lesions by transplanting exogenous NSCs, thus promoting the proliferation of endogenous NSCs.^296^ The key to determining the therapeutic effect is inhibiting NSC differentiation and enhancing BDNF secretion from transplanted NSCs. For this purpose, PCB-based PNPs were prepared to load ASO against lethal-7b (Let-7b) and simvastatin via electrostatic adsorption and diselenide bonds, respectively. A high level of ROS in NSCs^783^ mediated the breakage of diselenide bonds to release simvastatin,^784^ which promoted NSCs to secrete BDNF.^785^ The ASO against Let-7b downregulated Let-7b miRNA, thereby inhibiting the differentiation of NSCs through the upregulation of TLX.^776^ PNPs-treated NSCs were injected into the brain under the guidance of a stereotaxic instrument to promote BDNF secretion by exogenous NSCs in AD lesions, thereby facilitating the self-repair of neurons by promoting the proliferation of endogenous NSCs.

### Nucleic acid drug-based PD combination therapy

PD is the second most common ND characterized pathologically by the degeneration of discrete groups of neurons in the CNS, autonomic nervous system, and enteric nervous system.^786^ SNCA is the essential gene responsible for neurodegeneration and can induce neuronal degeneration by promoting the production of toxic α-synuclein aggregates.^787–791^ The degeneration of dopaminergic neurons in the SN is the primary pathogenesis of PD.^792,793^ In addition, α-synuclein aggregates promote the production of ROS,^794^ which in turn damages mitochondria^795^ and leads to α-synuclein aggregation,^794,796^ thereby resulting in a vicious cycle. Therefore, PD is also a complex disease with multiple therapeutic targets, which makes it suitable for nucleic acid drug-based combination therapy.

SNCA is the primary therapeutic target choice for PD gene therapy. siSNCA can downregulate α-synuclein expression by silencing the SNCA. In addition, curcumin inhibits α-synuclein aggregation, thereby preventing the loss of dopaminergic neurons.^797,798^ Our laboratory established a curcumin-siSNCA vector platform for the gene-chem synergistic treatment of PD. AuNP&PCB-based inorganic-organic hybrid nanoparticles were prepared for loading siSNCA and curcumin via electrostatic interactions and β-thiother ester bonds, respectively.^799^ The B6 peptide was modified to facilitate the vectors crossing of the BBB, and mazindol was modified to promote the vectors targeting dopaminergic neurons through DAT-mediated endocytosis.^800^ pH-sensitive PCB mediated siSNCA release to downregulate α-synuclein expression. ROS in diseased dopaminergic neurons accelerated the release of curcumin by cutting off β-thiother ester bonds, inhibiting the aggregation of α-synuclein. Subsequently, we applied exosomes to the co-loading of siSNCA and curcumin.^194^ Exosome/polymer hybrid core-membrane nanoparticles with RVG29 modification were prepared. The nanoparticle’s core is a BA-PDMAEA-based ROS-sensitive cationic PNP for loading curcumin and siSNCA via hydrophobic and electrostatic interactions, respectively. The outer membrane is a dendritic cell-derived exosome with RVG29 modification to promote BBB permeation and neuronal targeting uptake. In addition, dendritic cell-derived exosomes may also reduce the inflammatory response in PD lesions through their natural immunomodulatory effects,^801^ contributing to the combination therapy of PD with siSNCA and curcumin. Given the therapeutic effect of MSC-derived exosomes on PD,^802^ our laboratory again experimented with MSC-derived exosomes to prepare hybridized core-membrane nanoparticles.^267^ The exosome membranes were modified with penetratin (RQIKIWFQNRRMKWKK) and RVG to facilitate the penetration of the vectors from the nasal cavity into the brain and target neurons, respectively. MSC-derived exosome-mediated vectors were internalized by neurons via membrane fusion bypassed the endosome/lysosome pathway, thereby delivering endogenous miR-133b^803^ and PNPs cores directly to the cytoplasm. miR-133b exerted neuroprotective effects by promoting neuronal axon growth.^804,805^ ROS degraded PNPs in diseased neurons to release curcumin, thereby treating PD by inhibiting the aggregation of α-synuclein.

The microenvironment of PD lesions, such as ROS that promote α-synuclein aggregation and exosomes that propagate toxic α-synuclein aggregates, further exacerbate PD symptoms. Our laboratory applied ceria nano-enzymes to construct vectors for the direct removal of ROS in the focal microenvironment.^249^ Self-catalytic organic-inorganic hybrid nanoparticles were prepared for loading siSNCA. SiO_2_-coated SPIONs (SPIONs@SiO_2_) were synthesized as the core of the vectors to be modified with cerium oxide and functional polymers. Cerium oxide modification enabled the topography of the vectors to resemble neurotropic viruses such as the Japanese encephalitis virus, West Nile virus, and measles virus.^245–248^ Acetylcholine-like monomers were polymerized and then modified on SPIONs@SiO_2_ to mimic the ligands on the viral surface.^806,807^ These polymers were able to bind specifically to choline receptors on the surface of BBB endothelial cells and neurons.^808,809^ ATP-responsive cationic polymers were modified on the SPIONs@SiO_2_ surface for siSNCA loading. With the assistance of mimic neurotropic virus topography and ligands, the vector crossed the BBB and was targeted for neuron uptake. Cerium oxide downregulated ROS by scavenging free radicals in diseased neurons, thereby alleviating mitochondrial damage and inhibiting α-synuclein aggregation. Intracellular ATP mediated the release of siSNCA, thereby downregulating α-synuclein expression. In addition, inhibition of α-synuclein aggregates also reduced the level of inflammation in PD lesions, thereby synergistically treating PD.

Autophagy is an essential pathway to scavenge toxic α-synuclein aggregates.^810,811^ Downregulation of neutral sphingomyelinase-2 (nSMase2) can inhibit exosome production and thus block the transmission of toxic α-synuclein via exosomes.^812,813^ Thus, simultaneous enhancement of autophagy and inhibition of exosome generation hold promise for the combined treatment of PD. Our laboratory prepared synaptic vesicle-inspired LPNPs to load rapamycin to enhance autophagy and sinSMase2 to inhibit exosome synthesis.^272^ Transmembrane segment syb, derived from SNARE syb proteins of synaptic vesicles, was modified on the lipid membranes to enable synaptic vesicle-inspired membrane fusion.^270,271,814^ Rapamycin was delivered directly to the cytoplasm, which then promoted lysosome-mediated autophagy to eliminate α-synuclein aggregates.^815,816^ ROS mediated the release of sinSMase2 by degrading PNPs. sinSMase2 inhibited exosome production by downregulating nSMase2 expression, thereby blocking the spread of toxic α-synuclein aggregates. The researches of multiple drugs containing nucleic acid drugs for the synergistic treatment of NDs have been summarized in Table 6.

**Table 6:** Multi-drugs containing nucleic acid drugs synergistically loaded nanoparticles for NDs therapy

Vectors	Disease	Nucleic acid drug	Therapeutic target	Nucleic acid drug release	Other drug (s)	release	Ref(s)
PNPs	AD	siRNA	BACE1		Rapamycin		^155^
Lipid-based nanoparticles	AD	siRNA	BACE1		Curcumin		^757^
Organic-inorganic hybrid nanoparticles	AD	CRISPR/Cas9 plasmid	BACE1		Fluvastatin	Esterase response	^192^
PNPs	AD	siRNA	STAT3	ROS response	Fingolimod, and ZnO	ROS response	^235^
PNPs	AD	siRNA	NF-κB	ROS response	PHis, salsalate	Transition-metal ions response, cathepsin B response	^238^
PNPs	AD	siRNA	SOX9	pH response	Retinoic acid	Esterase response	^228^
PNPs	AD	ASO	Let-7b	ROS response	Simvastatin	ROS response	^296^
Organic-inorganic hybrid nanoparticles	PD	siRNA	SNCA	pH response	Curcumin	ROS response	^799^
Exosomes	PD	siRNA	SNCA	ROS response	Curcumin	ROS response	^194^
Exosomes	PD	miRNA	miR-133b		Curcumin	ROS response	^267^
Organic-inorganic hybrid nanoparticles	PD	siRNA	SNCA	ATP response	Ceria nano-enzymes		^249^
LPNPs	PD	siRNA	nSMase2	ROS response	Rapamycin	ROS response	^272^

## Nucleic acid drug-based brain disease nanotheranostics

**Fig. 7:**
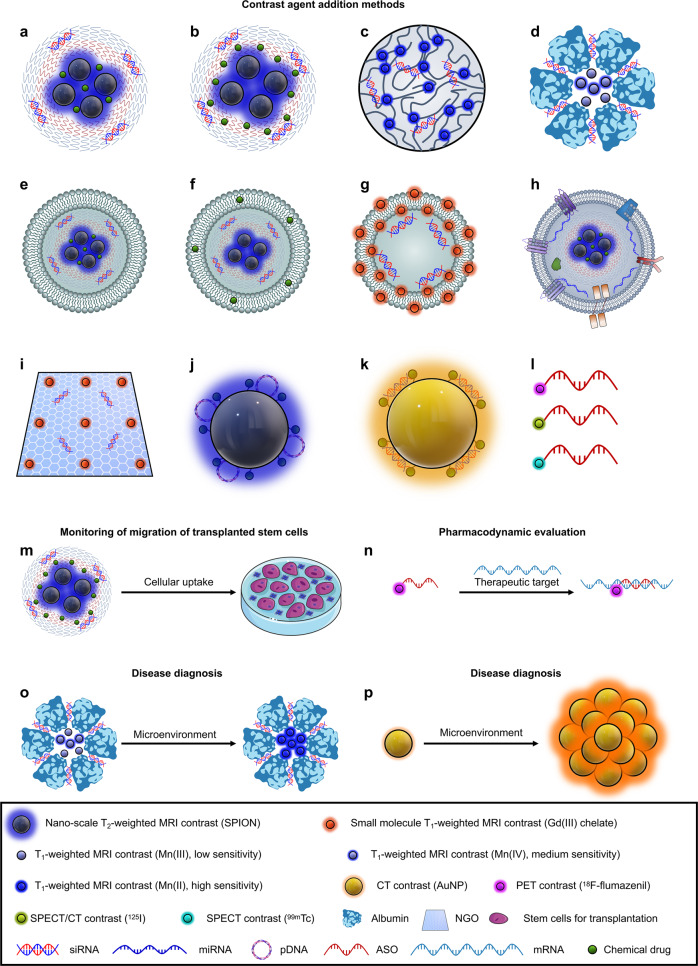
Schematic illustration of contrast addition methods and diagnostic applications. Contrast-labeled nucleic acid drug vectors are theoretically equipped for real-time monitoring of drug accumulation in the lesion. **a, b** T_2_-weighted MRI contrast agents (SPIONs) are encapsulated in the cores of multidrug-loaded PNPs by hydrophobic interactions. **c** T_1_-weighted MRI contrast agents (Mn(II) chelates) are used to cross-link cationic polymers into PNPs, thereby loading nucleic acid drugs. **d** The unactivated T_1_-weighted MRI contrast agents (Mn(III)) and the less sensitive T_1_-weighted MRI contrast agents (Mn(IV)) are adsorbed on albumin to form nanoparticles, thereby loading nucleic acid drugs. **e** Chemical drugs, nucleic acid drugs, and T_2_-weighted MRI contrast agents (SPIONs) are co-loaded in LPNPs. The contrast agents are encapsulated in the cores of PNPs by hydrophobic interactions. **f** Chemical drugs, nucleic acid drugs, and T_2_-weighted MRI contrast agents (SPIONs) are co-loaded in LPNPs. The contrast agents are encapsulated in the cores of PNPs by hydrophobic interactions. **g** T_1_-weighted MRI contrasts (Gd(III) chelates)-modified lipids are synthesized to construct cationic lipid-based nanoparticles to load nucleic acid drugs. **h** Chemical drugs, endogenous nucleic acid drugs, and T_2_-weighted MRI contrast agents (SPIONs) are co-loaded in exosome/polymer hybrid nanoparticles. The contrast agents are encapsulated in the cores of PNPs by hydrophobic interactions. **i** Nucleic acid drugs and T_1_-weighted MRI contrasts (Gd(III) chelates) are co-loaded on organic-inorganic hybrid nanoparticles via electrostatic interactions and covalent bonds, respectively. **j** T_2_-weighted MRI contrast agents (SPIONs) are developed as vectors based on organic-inorganic hybrid nanoparticles, thus loading chemical drugs and nucleic acid drugs via covalent bonds and electrostatic interactions, respectively. **k** CT contrast agents (AuNPs) are developed as vectors based on organic-inorganic hybrid nanoparticles, thus loading chemical drugs and nucleic acid drugs via covalent bonds and electrostatic interactions, respectively. **l** Radionuclides (^18^F-flumazenil, ^125^I or ^99m^Tc) are directly conjugated to nucleic acid drugs for nuclear medicine imaging (PET, SPECT/CT, or SPECT). **m-p** Diagnostic applications of the contrast-labeled nucleic acid drug vectors. **m** Monitoring of the migration of transplanted stem cells in vivo through the uptake of contrast-labeled nucleic acid drug vectors. **n** Pharmacodynamic evaluation by specific binding of contrast-labeled nucleic acid drug vectors to therapeutic targets. **o** The migration of transplanted stem cells in vivo can be monitored in real-time after the uptake of contrast-labeled nucleic acid drug vectors. **o** and **p** Disease diagnosis via disease microenvironment-activated signals from contrast agents in nucleic acid drug vectors

The efficient and precise delivery of nucleic acid drugs to their active sites is essential for maximizing the advantages of their high specificity for the therapeutic target. As previously mentioned, the delivery of nucleic acid drugs to the active site of diseased cells needs to overcome multiple physiological barriers such as non-specific proteins and immune surveillance in the circulation, the BBB, cellular uptake, endosomal/lysosomal escape, and drug release. However, individual differences between patients cause heterogeneity in the delivery performance of the same vector between different patients. In addition, the delivery performance of the same vector may also be heterogeneous across different disease courses in the same patient, which leads to severe uncertainties in the therapeutic efficacy of the same nucleic acid drug delivery system across patients. Many brain diseases are acute, hazardous, and/or even fatal, so feedback on the suitability of the nucleic acid drug delivery system for patients through therapeutic effects will delay the treatment. Therefore, real-time monitoring of the delivery of nucleic acid drugs to diagnose the patient’s suitability for the vectors is valuable for brain disease treatment. In addition, real-time monitoring of drug delivery in vivo in preclinical studies can also help guide the design, preparation, and screening of vectors in preclinical studies. The contrast addition methods and diagnostic applications were shown in Fig. 7.

### MRI contrast-based brain disease nanotheranostics

In brief, the atomic nucleus with an odd number of proton(s) (usually hydrogen proton) is excited in a magnetic field by a radio frequency (RF) pulse with a corresponding frequency to cause a resonance, i.e., nuclear magnetic resonance (NMR), resulting in a change in its energy level and phase. After the removal of the RF pulse, the energy level and phase return to the pre-excitation state, resulting in relaxation. The relaxation time associated with the energy level is called the longitudinal relaxation time or spin-lattice relaxation time (T_1_). The relaxation time associated with the phase is called the transverse relaxation time or spin-spin relaxation time (T_2_). The gradient magnetic field and the corresponding frequency of the RF pulse provide spatial localization for the NMR signal of these hydrogen protons. These space-localized MRI signals are acquired, processed, and image-reconstructed to produce MRI. Differences in signal intensities among different tissues and molecules contribute to the contrast in MRI. These signal intensities are mainly influenced by T_1_, T_2_, and proton density. While applying different RF pulse sequences results in MRI weighted by different influencing factors, such as proton density-weighted MRI, T_1_-weighted MRI, and T_2_-weighted MRI. MRI provides many advantages applicable to brain disease diagnosis, such as high contrast and high spatial resolution that can distinguish different brain regions, non-invasive and safe imaging, and multi-parametric&sequence imaging that can provide more diagnostic information, thus becoming the preferred medical imaging tool for the clinical diagnosis of brain diseases.

In addition to designing different sequences of RF pulses, the administration of contrast agents can also enhance the contrast between different tissues, especially between diseased and normal tissues. Contrast agents improve the contrast of MRI mainly by influencing the relaxation time of surrounding protons. Therefore, MRI contrast agents are mainly used for T_1_-weighted and T_2_-weighted imaging. Gd-containing metal-organic chelates, such as Gd-DOTA, are typical T_1_ contrast agents.^817^ While MIONs, such as SPIONs, are typical T_2_ contrast agents.^818^ MRI contrast agents can be added as probes in nucleic acid drug vectors to trace the accumulation of the vector in brain lesions in vivo by MRI in real-time.

Gd-containing chelates were the first MRI contrast agents applied to nucleic acid drug vectors. Gd-containing chelates can shorten the T_1_ of surrounding hydrogen protons, thus brightening this region. Hart et al. applied Gd chelate-modified lipid (Gd-DTPA-DSOA) to the assembly of cationic liposomes. Luciferase pDNA was transfected through these liposomes into U87-MG cells. These transfected cells were then injected into the brain under the guidance of stereotaxic localization. T_1_-weighted MRI could monitor the migration of these transfected cells in the brain.^819^ Ma et al. prepared nanographene oxide modified by dendrimer and Gd chelate (Gd-DPTA) to load with Let-7g miRNA and epirubicin for the GBM diagnosis and treatment.^820^ Let-7g miRNA was able to exert anti-tumor effects through the expression of the Ras oncogene family.^821–824^ FUS was implemented to open the BBB temporarily. Gd-functionalized nanoscale graphene oxide (Gd-NGO) was first injected to verify the opening of the BBB by T_1_-weighted MRI. Subsequently, the vectors were injected via the tail vein for GBM combination therapy. T_1_-weighted MRI monitored the accumulation and distribution of the vectors in the brain. Despite the high T_1_-weighted MRI sensitivity of Gd chelates, their short half-life and nephrotoxicity should be considered with caution. Mn-containing nanoparticles have also been applied for T_1_-weighted MRI. Mn chelators cross-linked chitosan to form PNPs and loaded with siRNA or dsRNA.^825^ T_1_-weighted MRI monitored the PNPs’ distribution in the brain after intranasal administration. Obvious T_1_-weighted MRI signals were observed in the olfactory bulb, hippocampus, cerebral cortex, and striatum, indicating that chitosan-based PNPs could penetrate efficiently from the nasal cavity into the brain. Mn(III) and Mn(IV) ions adsorbed by bovine serum albumin (BSA) were able to be endowed with the tumor microenvironment (TME)-sensitive T1-weighted MRI signals. cRGD-modified BSA was formed into nanoparticles with Mn(III) and Mn(IV) and loaded with siRNA, and then delivered Mn(III), Mn(IV), and siRNA to GBM. The GBM acidic microenvironment could mediate the disproportionation of Mn(III) to Mn(II) and Mn(IV). Mn(IV) reacted with endogenous H_2_O_2_ in situ to generate oxygen and Mn(II), which reduced acidification and ROS of TME, contributing to GBM treatment. Mn(II) generated under TME improved the T_1_-weighted MRI signal, thus achieving TME-sensitive T_1_-weighted MRI for GBM diagnosis and treatment.^826^

MIONs are commonly applied as T_2_-weighted imaging contrast agents by significantly influencing T_2_. In particular, SPIONs exhibit more sensitive and accurate contrast-enhanced MRI due to the absence of hysteresis and higher magnetic susceptibility. MIONs can shorten T_2_ by influencing the relaxation process of surrounding hydrogen protons, thereby darkening the T_2_-weighted MRI in this region. MIONs can be prepared by aqueous-phase synthesis methods, such as micro-emulsion, gel-sol, sonochemistry, and coprecipitation methods, and non-aqueous-phase synthesis methods, such as the high-temperature thermo-decomposition method. The aqueous-phase synthesis is to obtain MIONs by hydrolyzing Fe^3+^ in the aqueous phase. These methods enable water-soluble MIONs through simple preparation processes and therefore do not require phase inversion. However, the strong coordination ability of water and OH^-^ with Fe^3+^ during the synthesis process causes complex surface structures and complicated functionalization modifications of MIONs. In addition, the aqueous-phase synthesis cannot obtain MIONs with homogeneous and small shapes and sizes, which is unsuitable for brain disease diagnosis. Furthermore, the relatively low temperature limited by the boiling point of water during crystallization results in low crystallinity and correspondingly low magnetic susceptibility of MIONs. Non-aqueous phase synthesis prepares MIONs by high-temperature decomposition of metal-organic iron compounds in non-polar or weakly polar organic solvents with high boiling points. The homogeneity, crystallinity, and magnetic susceptibility of MIONs prepared by this method are significantly better than those prepared by the aqueous phase synthesis method. In addition, the non-aqueous phase synthesis enables control of the size and shape of MIONs, thus obtaining MRI contrast agents suitable for brain disease diagnosis. However, hydrophobic ligands with long alkyl chains are introduced to improve the compatibility of MIONs with organic solvents, which prepares hydrophobic MIONs that need phase inversion for MRI application. However, due to the remarkable advantages in MRI sensitivity and the continuous development of phase inversion methods, MIONs prepared by non-aqueous phase synthesis are gradually becoming the preferred choice for T_2_ contrast agents. In addition, non-aqueous phase synthesis methods of hydrophilic MIONs have also been developed by introducing polar organic solvents and hydrophilic organic ligands.^827,828^

Amphiphilic polymers modified on the surface of MIONs by hydrophobic interactions are the most common approach for phase inversion of MIONs. MIONs-labeled nucleic acid drug vectors were first applied to trace the migration of transplanted cells in the brain in real-time. Our laboratory has established PCB-modified SPIONs as traceable gene-chem vectors. The PCB-based amphiphilic block copolymers were modified on the SPIONs to load siSOX9 and retinoic acid under acidic conditions.^228^ After the uptake of the SPION-labeled gene-chem vectors, NSCs were injected into the brain under stereotactic guidance for AD treatment. The migration of NSCs to the lesion was observed in real-time in T_2_-weighted MRI. The SPION-labeled gene-chem vectors, which replaced chemotherapy and nucleic acid drugs with simvastatin and Let-7b ASO, respectively, were also capable of tracing the migration of NSCs in the brain by T_2_*-weighted MRI in real-time,^296^ demonstrating the universality of this traceable vectors. Teng et al. modified SPIONs by PEI-based amphiphilic polymers to load siRNA through hydrophobic interaction.^829^ SPIONs-labeled vectors downregulated HIF-prolyl hydroxylase 2 (PHD2) expression after uptake by endothelial progenitor cells (EPCs), thereby enhancing the survival of EPCs in ischemic environments via the HIF-1α-dependent pathway. Transfected EPCs were injected into the mice’s left ventricle to treat ischemic stroke. Interestingly, silencing PHD2 promoted CXCR4 expression, which contributed to the homing of EPCs, while T_2_-weighted MRI observed the migration of intracardiac transplanted EPCs to the peri-infarct area in the brain. Shuai et al. also prepared PEI-based amphiphilic cationic copolymers to phase-invert SPIONs and load siRNAs.^830^ These SPION-labeled nucleic acid drug vectors were applied for stroke treatment after uptake by NSCs. T_2_-weighted MRI also observed the migration of NSCs in the brain. To enhance the safety of the vector, Shuai et al. replaced the PEI-based polymer with a biodegradable poly(amino acid)-based polymer and traced the migration of NSCs in the brain in real-time again.^831^ The introduction of SPIONs provided real-time evidence for the capability of NSCs to migrate to lesions and offered guidance for applying stem cell transplantation therapies to diagnosing and treating CNS diseases.

SPIONs have also been applied for real-time monitoring of the accumulation of nucleic acid drug delivery systems in the brain. Our laboratory has established the methods to introduce SPIONs into PNPs, LPNPs, exosome/polymer hybrid nanoparticles, and organic-inorganic hybrid nanoparticles for the combined diagnosis and treatment of GBM, AD, and PD. SPIONs can only be encapsulated in the nanoparticles’ hydrophobic domain because of their hydrophobicity. Only the outer membrane’s lipid bilayer and polymeric core are hydrophobic in LPNPs. Consequently, nanoscale SPIONs can only be encapsulated within the polymeric core of LPNPs. After surface modification of LPNP-based traceable gene-chem vectors with different ligands, such as Ang and RVG29, T_2_*-weighted MRI showed the accumulation of vectors in the GBM and SN regions of the brain, respectively.^183,272^ Amphiphilic block copolymers were modified on the surface of SPIONs to encapsulate hydrophobic SPIONs into hydrophilic exosome cavities.^267^ The hydrophilized SPIONs aggregates were encapsulated within MSC-derived exosomes under ultrasound conditions. After intranasal administration, the vector distribution in the brain was monitored by SPION-enhanced T_2_*-weighted MRI.

Amphiphilic polymers do not convert hydrophobic SPIONs to hydrophilic SPIONs, but rather assist in the dispersion of hydrophobic SPIONs in water. In addition, the addition of contrasts to vectors renders the MR signal indicative of the distribution of SPIONs in vivo rather than the distribution of the vectors, thereby increasing the risk of probe leakage artifacts. To address these issues, our laboratory modified functional polymers on the surface of SiO_2_-coated SPIONs by covalent bonding, thus obtaining SPION-based nucleic acid drug vectors for the combined diagnosis and treatment of PD.^249^ Since the vectors confer MRI sensitivity, there are no artifacts caused by probe leakage, enabling more accurate tracing of vector distribution in the brain by T_2_*-weighted MRI. However, the method to prepare such vectors is somewhat complicated, and the SiO_2_ shell may weaken the magnetic sensitivity of the vectors by blocking the proximity of hydrogen protons to SPIONs. Our laboratory developed magnetic nano-biohybrid complexes to overcome these concerns through a one-step synthesis.^192^ The dopamine-modified PLys replaced the hydrophobic ligands on SPIONs with competitive coordination bonds, synthesizing hydrophilic and monodisperse SPIONs as gene-chem drug vectors. The T_2_ relaxation rate of the hydrophilic SPIONs prepared by the one-step synthesis was increased by nearly 60% compared with that of the SPION aggregates prepared by amphiphilic polymer-mediated hydrophobic interactions, which was attributed to the replacement of the original hydrophobic ligands on the surface of SPIONs by PLys, which resulted in the adsorption of numerous hydrogen protons around the SPIONs through hydrophilic interactions and hydrogen bonds, substantially shortening the T_2_ of these hydrogen protons. In addition, the hydrophilic SPIONs produced by the one-step synthesis were monodisperse in water without aggregation due to the absence of hydrophobic ligands. Therefore, the hydrophilic SPION-based gene-chem vectors prepared by the one-step synthesis exhibited more sensitive signals in T_2_*-weighted MRI of the brain.

### Other probe-based brain disease nanotheranostics

Positron emission tomography (PET) and SPECT are typical clinical radionuclide imaging techniques applied to diagnose tumors, especially metastases, by detecting physiological and metabolic activity. Compared to PET, SPECT can spatially localize, use different nuclides, and produce rays with different energies, thus providing more diagnostic information. However, the high-energy radioactive γ-rays during PET and SPECT damage the body. By conjugating [^18^F]F-537-Tz, the biodistribution of ASOs in the brain could be monitored by PET in real-time.^832^ Radionuclide-conjugated nucleic acid drugs can also diagnose and treat diseases by imaging the distribution of therapeutic targets in the brain and downregulating the expression of therapeutic targets, respectively. HOTAIR is a long non-coding RNA that is specifically highly expressed in GBM.^833^ In addition, HOTAIR expression upregulates with increasing malignancy of GBM.^834^ The HOTAIR distribution and content in the brain could be observed by SPECT through intravenous injection of ^99m^Tc-conjugated ASO against HOTAIR, thus providing valid diagnostic information for GBM.^835^ The pharmacokinetics and pharmacodynamics of nucleic acid drugs can be accurately detected and evaluated by in vivo SPECT, PET-based multi-modal imaging. Verma et al. defined the 2-dimensional and 3-dimensional spatiotemporal pharmacokinetics of intrathecal delivered ASO penetrating the CNS and interactions between ASO and multiple molecular motion pathways by in vivo SPECT, ex vivo immunohistochemistry (IHC), and cryofluorescence tomography. In addition, they validated the pharmacodynamics of ASO by PET imaging using nucleophiles capable of binding specifically to ASO targets.^836^

CT is also a common clinical imaging technique to diagnose diseases. The unique advantage of CT is the rapid three-dimensional reconstruction of focal tissue imaging. Nevertheless, high-energy X-rays can also cause damage to the body. AuNPs are typical CT contrast agents and nucleic acid drug vectors. Our laboratory prepared polymer-modified AuNPs co-loaded with curcumin and siSNCA for diagnosing and treating PD.^799^ Considering the higher concentration of Fe(III) in PD lesions than in normal tissue,^837^ levodopa-modified AuNPs were employed as CT contrast agents due to their sensitivity to Fe(III).^838^ Curcumin and siSNCA were delivered to dopaminergic neurons in the brain under the guidance of B6 peptide and mazindol. After drug release, exposed levodopa on AuNPs mediated AuNPs aggregation through specific binding to high levels of Fe^3+^ in diseased neurons by coordination, thus improving the CT sensitivity of the contrast agent. The sensitized CT signals were observed only in the brains of PD mice, indicating that these AuNP-based gene-chem vectors were not only capable of in vivo monitoring of drug accumulation in the brain but also contributed to the PD diagnosis. The researches on nucleic acid drug vectors for simultaneous diagnosis and treatment have been summarized in Table 7.

**Table 7:** Nucleic acid drug delivery nanoparticles for simultaneous diagnosis and therapy

Vectors	Disease	Nucleic acid drug	Therapeutic target	Other drug(s)	Contrast(s)	Medical imaging	Role	Ref(s)
Lipid		pDNA			Gd-DTPA	T_1_-weighted MRI	Monitor the migration of transfected cells	^819^
Inorganic nanoparticles	GBM	miRNA	Let-7g	Epirubicin	Gd-DPTA	T_1_-weighted MRI	Diagnosis	^820^
PNPs		siRNA, dsRNA			Mn chelators	T_1_-weighted MRI	Diagnosis	^825^
Organic-inorganic hybrid nanoparticles	AD	siRNA	SOX9	Retinoic acid	SPIONs	T_2_-weighted MRI	Monitor the migration of transfected cells	^228^
Organic-inorganic hybrid nanoparticles	AD	ASO	Let-7b	Simvastatin	SPIONs	T_2_-weighted MRI	Monitor the migration of transfected cells	^296^
Organic-inorganic hybrid nanoparticles	Ischemic stroke	siRNA	PHD2		SPIONs	T_2_-weighted MRI	Monitor the migration of transfected cells	^829^
Organic-inorganic hybrid nanoparticles	Stroke	siRNA	NgR		SPIONs	T_2_-weighted MRI	Monitor the migration of transfected cells	^830^
Organic-inorganic hybrid nanoparticles	Stroke	siRNA	Pnky		SPIONs	T_2_-weighted MRI	Monitor the migration of transfected cells	^831^
Exosome/polymer hybrid nanoparticles	PD	miRNA	miR-133b	Curcumin	SPIONs	T_2_-weighted MRI	Trace the distribution of vectors	^267^
LPNP	GBM	siRNA	TGF-β	TMZ	SPIONs	T_2_-weighted MRI	Trace the distribution of vectors	^183,272^
Organic-inorganic hybrid nanoparticles	PD	siRNA	nSMase2	Rapamycin	SPIONs	T_2_-weighted MRI	Trace the distribution of vectors	^249^
Organic-inorganic hybrid nanoparticles	AD	CRISPR plasmids	BACE1	Fluvastatin	SPIONs	T_2_-weighted MRI	Trace the distribution of vectors	^192^
Conjugate		ASOs			[^18^F]F-537-Tz	PET	Diagnose	^832^
Conjugate	GBM	ASOs	HOTAIR		^99m^Tc	SPECT	Diagnose	^835^
Conjugate		ASOs	anti-GluR1 and anti-Gabra1		^125^I	SPECT	Validate the pharmacodynamics	^836^
Organic-inorganic hybrid nanoparticles	PD	siRNA	SNCA	Curcumin	AuNPs	CT	Trace the distribution of vectors	^799^

## Conclusion and future perspectives

This Review discussed multiple nucleic acid drug vectors designed and functionalized approaches for brain diseases to overcome multiple physiological barriers in vivo. The peculiarities of nucleic acid drugs inherently lead to the necessity of vector-dependent transport in vivo. The particularities of brain diseases bring additional challenges and concerns to the delivery of nucleic acid drugs and put higher requirements on the precise delivery of nucleic acid drugs. The prerequisite for developing nucleic acid drug vectors is understanding the multiple physiological barriers faced by the vectors after administration to guide the development of suitable vectors, optimization of the vectors’ performance, and multifunctional modification to overcome these physiological barriers. The essence is to control the interactions among vectors, drugs, and physiological barriers such as tissues, cells, and molecules. Therefore, vector development requires multidisciplinary integration. In addition to chemistry, pharmacology, and materials science, the support of cell biology, molecular biology, biomechanics, and other disciplines is essential. BBB, which is closely related to brain diseases, and endosome/lysosome escape, which is closely related to nucleic acid drugs, need to be emphasized in vector development. In addition, taking inspiration from nature and extracting natural components from organisms as vectors have shown prominent advantages. Synthetic biology has significant potential in the design and production of such vectors. Targeted design, reform, and re-synthesis of organisms or cells through a synthetic biology approach with an engineering design concept are expected to confer the desired properties of biologically derived vectors that incorporate the advantages of multiple bionic vectors and overcome the natural defects of biologically derived vectors. In addition, synthetic biology approaches are expected to guide organisms or cells to produce nucleic acid drugs and vectors, thus biosynthesizing one-step vectors loaded with nucleic acid drugs and promising to overcome the defects of vector heterogeneity.

Applying strategies that overcome physiological barriers to the design and preparation of vectors is a crucial issue in vector development. Firstly, it is necessary to clarify the advantages and shortcomings of different types of vectors. Secondly, it is necessary to clarify the advantages and shortcomings of the different forces, such as hydrophobic interactions, electrostatic interactions, covalent bonds, and hydrogen bonds, of each component forming the vectors and nucleic acid drug loading. Thirdly, molecules are the basis for determining vector properties and functions, such as polymers in PNPs and lipids in lipid-based nanoparticles. The methods for modifying functional molecules on vectors, such as ligand molecules and microenvironment-sensitive molecules, also need to be grasped. The biocompatibility and biodegradability of the molecules must be considered. Extraction and isolation methods, loading methods of exogenous molecules, such as nucleic acid drugs and ligand molecules, and the manners of using EVs as vectors still need continuous innovation. The distinct advantage of inorganic nanoparticle-based vectors is their readily tunable homogeneous size, shape, and topography, while functionalized modifications by organic molecules on inorganic nanoparticles are still challenging and need to be studied. As for nucleic acid drug conjugates, the design of the functional molecules for conjugation is a prerequisite, while efficient and gentle conjugation reactions are the focus. Besides, conjugates are currently only applied to synthetic nucleic acid drugs with relatively low molecular weights, such as siRNAs and ASOs. Applying conjugates to other nucleic acid drugs with a high molecular weight, such as mRNA and pDNA, is a significant and challenging coexisting aspect. Significantly, vector development cannot be limited to existing vector types. New types of vector development and hybridization of multiple types of vectors should be emphasized.

For most brain disease treatments, nucleic acid drugs are not substitutes for conventional drugs but a powerful complement. A single nucleic acid drug may not meet therapeutic expectations for brain diseases with complex pathogenesis and multiple therapeutic targets. Therefore, combining nucleic acid drugs with conventional drugs is particularly significant for these brain diseases. For example, the current drug therapy for glioma is still dominated by chemical drugs. Nucleic acid drugs can take advantage of their high specificity for therapeutic targets to enhance the efficacy of GBM chemotherapy through immunomodulation and/or reduce the resistance of GBM to chemotherapy. In addition, nucleic acid drugs can be used as activators for certain small molecule inert prodrugs to improve efficacy while reducing side effects. Furthermore, combination therapy can also be developed to treat brain diseases synergistically through a multi-pronged approach. Therefore, selecting the optimal therapeutic strategy is crucial for the precise combination therapy of brain diseases. However, combination therapy also poses new challenges for vectors. In addition, the active sites and times for different drugs may be different, resulting in poor therapeutic efficacy with a single drug release approach. Therefore, precise drug release in a programmed and spatiotemporally controlled manner is essential for the full efficacy of multidrug delivery, which requires adequate studies of the microenvironment at different focal sites, the microenvironmental sensitivity of the functional groups responsible for drug release in the vectors, and extensive validation and screening. In addition, the hybridization of multiple types of vectors may contribute to the co-loading and precise release of multiple drugs. Therefore, it is necessary to fully grasp the physicochemical properties and functionalization methods of multiple types of vectors. The construction of novel types of vectors and the hybridization of multiple types of vectors are even more significant in multidrug co-delivery. The high specificity and low off-targeting of nucleic acid drugs to therapeutic targets are prerequisites for gene-based combination therapies. However, the complexity and big data of genomics lead to substantial time consumption and high costs for traditional screening of nucleic acid drug sequences. Advances in artificial intelligence and machine learning technologies promise to enable rapid and low-cost screening of nucleic acid drugs, thereby improving their therapeutic efficacy and avoiding the risks associated with off-targeting.

Due to individual differences among patients, achieving efficient treatment of the same diseases with one vector and nucleic acid drugs against a single therapeutic target is unrealistic. Therefore, patient stratification is a prerequisite for precision medicine. Multiple vectors, multiple functionalization approaches, nucleic acid drugs targeting multiple therapeutic targets, and multiple combination therapeutic strategies have been extensively studied. However, precise and rapid vector and drug screening for individual patients in clinical therapy is complicated, necessitating real-time access to precise delivery information of vectors in patients and real-time feedback on therapeutic efficacy. Medical imaging and the corresponding contrast agents, represented by MRI and SPIONs, offer the promise of in vivo tracing of vectors and timely feedback on therapeutic effects. The combination of diagnostics and therapeutics through introducing contrast into vectors (nucleic acid drug-based nanotheranostics) is expected to enable precise and personalized treatment of brain diseases. However, most nanotheranostics are currently only able to monitor the vectors’ distribution in the brain. The release and pharmacodynamics of nucleic acid drugs are still difficult to monitor in real time. In addition, the contrast sensitivity and artifacts caused by leakage from vectors are also challenges for nanotheranostics. Improving the imaging sensitivity and precision of contrast agents is still the priority of nanotheranostics research and development, which requires understanding the principles of medical imaging and contrast-enhanced medical imaging, as well as rethinking and innovating the manners in which contrast agents are introduced into vectors. In addition, providing more diagnostic information through nanotheranostics and medical imaging, thereby providing more precise screening of vectors and drugs suitable for individual patients, promises to support precision treatment of brain diseases, which may require the assistance of multi-modal imaging and corresponding contrast agents.

In clinical trials for brain diseases, nucleic acid drugs are predominantly delivered by viral vectors and vector-free delivery. For instance, UniQure Biopharma B.V. utilizes rAAV as a miHTT vector (rAAV5-miHTT) in ongoing phase 1 and 2 clinical trials for the treatment of HD (NCT04120493 and NCT05243017). Neurologix, Inc. conducted a phase 2 clinical trial using AAV as a vector for the glutamic acid decarboxylase (GAD) gene in patients with Parkinson’s disease (NCT00643890). However, topical delivery of these viral vectors requires stereotaxic guidance, and patient compliance is poor. ASO has been administered vector-free in clinical trials for a variety of brain diseases. For instance, Novartis Pharmaceuticals conducted clinical trials with ASO, which targeted tau in numerous CNS diseases (NCT04539041, NCT05469360). Wave Life Sciences Ltd. conducted clinical trials for Amyotrophic Lateral Sclerosis and HD with WVE-003 (ASO targeting HTT) and WVE-003 (ASO targeting C9orf72), respectively (NCT05032196 and NCT04931862). Stoke Therapeutics, Inc. conducted a clinical trial of STK-001, an ASO targeting SCN1A, to treat Dravet syndrome. All these ASOs devoid of vectors were administered intrathecally. Thus, the delivery of nucleic acid drugs via viral vectors or without vectors is highly invasive. In contrast, Northwestern University conducted an early phase 1 clinical trial of gliosarcoma via intravenous administration of siBcl2L12 vectors loaded with gold nanoparticles. Therefore, the research and development of non-invasive or minimally invasive delivery vectors facilitate the clinical translation of nucleic acid drugs for brain diseases. Additionally, vectors derived from organisms, such as exosomes, have low immunogenicity, low toxicity, and provide natural delivery of nucleic acid molecules. Particularly, exosomes derived from the same patient can reduce immune rejection following drug administration in the same patient. Therefore, such vectors have a strong likelihood of clinical application. The clinical translation of such vectors is, therefore, promising. Moreover, exosome-loaded nucleic acid drugs require synthetic biology support.

Overall, the high specificity of nucleic acid drugs against therapeutic targets and the development of precisely designed and functionalized vectors hold promise for the precision treatment of brain diseases. The precise design and functionalization of vectors, the precise treatment of nucleic acid drugs against single or multiple therapeutic targets, and the precise diagnosis by medical imaging and corresponding contrast agents are the basis for precision medicine in brain diseases. Significantly, these three aspects should be integrated rather than independent.
